# Footprints of Human Migration in the Population Structure of Wild Baker's Yeast

**DOI:** 10.1111/mec.17669

**Published:** 2025-02-04

**Authors:** Jacqueline J. Peña, Eduardo F. C. Scopel, Audrey K. Ward, Douda Bensasson

**Affiliations:** ^1^ Department of Plant Biology University of Georgia Athens Georgia USA; ^2^ Institute of Bioinformatics University of Georgia Athens Georgia USA; ^3^ Center for Genomic Science Innovation University of Wisconsin‐Madison Madison Wisconsin USA; ^4^ Department of Genetics University of Georgia Athens Georgia USA

**Keywords:** ecological genomics, genetic admixture, phylogeography, *Saccharomyces cerevisiae*, wine yeast, yeast ecology

## Abstract

Humans have a long history of fermenting food and beverages that led to domestication of the baker's yeast, *Saccharomyces cerevisiae* . Despite their tight companionship with humans, yeast species that are domesticated or pathogenic can also live on trees. Here we used over 300 genomes of *S. cerevisiae* from oaks and other trees to determine whether tree‐associated populations are genetically distinct from domesticated lineages and estimate the timing of forest lineage divergence. We found populations on trees are highly structured within Europe, Japan, and North America. Approximate estimates of when forest lineages diverged out of Asia and into North America and Europe coincide with the end of the last ice age, the spread of agriculture, and the onset of fermentation by humans. It appears that migration from human‐associated environments to trees is ongoing. Indeed, patterns of ancestry in the genomes of three recent migrants from the trees of North America to Europe could be explained by the human response to the Great French Wine Blight. Our results suggest that human‐assisted migration affects forest populations, albeit rarely. Such migration events may even have shaped the global distribution of *S. cerevisiae* . Given the potential for lasting impacts due to yeast migration between human and natural environments, it seems important to understand the evolution of human commensals and pathogens in wild niches.

## 1 Introduction

Since the last ice age, humans have transitioned from a hunter‐gatherer to a sedentary lifestyle and developed new technologies for preserving food, including fermentation (McGovern 2003). The baker's yeast, *Saccharomyces cerevisiae*, is a driver of such fermentations and is used to produce beer, wine, sake, cocoa, and coffee (Marsit et al. 2017). The earliest archaeological evidence of fermented rice, honey, and fruit in China dates to 7000 BCE (McGovern et al. 2004). Fermented beer was first discovered in ancient Sumerian vessels from 6000 BCE (Michel, McGovern, and Badler 1992), and there is evidence for wine production between 6000 and 4000 BCE in Iran, the Caucasus, and Mesopotamia (Pretorius 2000; McGovern 2003). Wine production then spread throughout the Mediterranean and was prevalent across Europe and Northern Africa by 500 BCE (Pretorius 2000).

Today, the population genetics of *S. cerevisiae* shows imprints of domestication with several genetic lineages associated with distinct domestication events (Almeida et al. 2015; Duan et al. 2018; Fay et al. 2019; Fay and Benavides 2005; Gallone et al. 2016; Gayevskiy, Lee, and Goddard 2016; Legras et al. 2007, 2018; Liti et al. 2009; Peter et al. 2018; Schacherer et al. 2009). Deep sampling of wild strains from Chinese and Taiwanese forests revealed high levels of lineage diversity compared to all other lineages, and the current consensus is that East Asian forests likely harboured the ancestral source populations that gave rise to all global *S. cerevisiae* lineages (Duan et al. 2018; Lee et al. 2022; Wang et al. 2012). Even outside Asia, sampling of *S. cerevisiae* from natural environments shows a wild side to this human‐associated yeast species; there are wild genetic lineages that are distinct from known domesticated lineages (Almeida et al. 2015; Cromie et al. 2013; Fay and Benavides 2005; Han et al. 2021; Liti et al. 2009; Peter et al. 2018; Tilakaratna and Bensasson 2017).

How much has human activity affected the ecology and evolution of wild populations of human‐associated yeast species? Human pathogenic yeast such as *Candida albicans* , *Candida glabrata*, *Candida parapsilosis* , and *Candida tropicalis* can be isolated from trees and other plant habitats (Bensasson et al. 2019; Opulente et al. 2019; Robinson, Pinharanda, and Bensasson 2016), and other forest yeast species are associated with humans (Boynton and Greig 2014; Mozzachiodi et al. 2022). Here, we make use of the extensive genome data available for *S. cerevisiae* and focus on the population structure and phylogenetic relationships of wild *S. cerevisiae* from a single ecological niche. By studying strains from oaks and other trees, we characterise populations in an ancestral niche while avoiding the complications of genetic admixture more commonly seen in *S. cerevisiae* from fruit, flowers, and insects (Hyma and Fay 2013; Tilakaratna and Bensasson 2017). Specifically, we identified tree‐associated populations and estimated the timing of wild yeast migration events. We show that (i) tree‐associated *S. cerevisiae* populations are highly structured, (ii) the worldwide spread of forest populations out of Asia probably occurred since the last glacial maximum, and lastly (iii) human‐assisted migration is ongoing and may include migration from the USA to Europe since the Great French Wine Blight.

## 2 Materials and Methods

### 2.1 Yeast Strains and Genome Data

Whole‐genome sequences for strains sampled from trees were compiled from publicly available data (*N* = 295; Table S1) (Almeida et al. 2015; Barbosa et al. 2016; Bergström et al. 2014; Duan et al. 2018; Fay et al. 2019; Gayevskiy, Lee, and Goddard 2016; Han et al. 2021; Pontes et al. 2020; Skelly et al. 2013; Song et al. 2015; Strope et al. 2015; Yue et al. 2017). We defined *S. cerevisiae* tree‐sampled strains as those isolated from tree bark, exudate, and leaves from trees or litter, and we also included strains from any soil. Metadata was compiled for each genome sequence to include geographical origin, ecological substrate, and previously reported genetic clade associated with the strain (Table S1). New whole‐genome sequence data was generated for strains from trees in Indiana and Kentucky (*N* = 7; Osburn et al. 2018), North Carolina (*N* = 9; Diezmann and Dietrich 2009), and Europe (*N* = 3; Robinson, Pinharanda, and Bensasson 2016), and for new *S. cerevisiae* strains from the bark of white oak (*Quercus alba*) and live oak ( *Q. virginiana* ) from Georgia, Florida, Pennsylvania, and North Carolina (*N* = 15; Bensasson lab). DNA was extracted from single yeast colonies using the Promega Wizard Genomic DNA purification kit following the manufacturer's protocol for yeast except that only 75 units of lyticase (Sigma) were used in an overnight incubation at 37°C. For the generation of genome data from 22 strains from the Bensasson and Osburn labs, paired‐end Illumina libraries were generated by the Georgia Genomics and Bioinformatics Core using the purePlex DNA Library Preparation Kit (GGBC Project #5256) or the Nextera DNA‐Seq Library Protocol (GGBC Project #5881). Paired‐end sequencing was performed on the Illumina NextSeq2000 platform (2 × 150 bp). The remaining strains were sequenced at the University of Manchester as described in Almeida et al. (2015). Genome data is available on NCBI‐SRA under project number PRJNA1090965 and includes a further 8 strains from Pennsylvania that are monosporic derivatives of previously studied strains (Table S1; Sniegowski, Dombrowski, and Fingerman 2002).

To examine how strains from trees are related to other yeast strains, we constructed a reference panel of strains to represent published clades (1030 strains from 42 clades; Duan et al. 2018; Peter et al. 2018). These reference‐panel strains were isolated from the human body (clinical), fermentation (e.g., wine and beer), baking, bioethanol, crops (e.g., sugar cane), decaying wood, fruit, flowers, insects, mushrooms, and water (e.g., sewers and oceans).

### 2.2 Read Mapping and Base Calling

Paired‐end and single‐end genomic Illumina reads were downloaded from the European Bioinformatics Institute (https://www.ebi.ac.uk/) or generated in this study. Reads were mapped to the *S. cerevisiae* reference genome, S288c (SacCer_Apr2011/sacCer3 from UCSC), using Burrows‐Wheeler Aligner (bwa‐mem, version 0.7.17; Li and Durbin 2009). We used SAMtools to sort, index, and compress bam files and generated a consensus sequence using the mpileup function with the ‐I option to exclude indels (version 1.6; Li et al. 2009). Next, we used the BCFtools call function with the ‐c option to generate a consensus sequence (version 1.9) (Li et al. 2009) and converted from vcf format to fastq format in SAMtools using the “vcfutils.pl vcf2fq” command. Lastly, base calls with a phred‐scaled quality score of less than 40 were treated as missing data (calls were converted to “N”) using seqtk seq ‐q 40 in SAMtools. In practice, this probably leads to an overall error rate lower than 1 in 10,000 because high‐depth Illumina sequences usually yield consensus base calls that are not close to the Q40 cutoff. For example, after applying a Q40 cutoff to our past Illumina sequencing of *Candida albicans,* we observed an error rate below 1 in 100,000 (< 169 errors in 14 Mbp; Bensasson et al. 2019), and use of the Q40 threshold on Sanger sequence from *S. cerevisiae* yielded a similarly low error rate (< 1 in 180,000 bp; Bensasson 2011). None of the analyses discussed here would be affected by such a low error rate.

### 2.3 Quality Filtering Steps

For population structure analysis, we removed genome sequences if they were from tree‐sampled strains already represented in the dataset (*N* = 27), from strains with no geographical information (*N* = 1), or if they had an average read depth below 30× (*N* = 18). Additionally, genome data were visualised to check for intraspecies cross‐contamination using vcf2allelePlot.pl. (Bensasson et al. 2019; Scopel et al. 2021): a genome was removed (*N* = 18) if some reads produced unexpected SNP calls at a frequency over 1%. For the remaining strains sampled from trees, we estimated genome‐wide levels of heterozygosity using vcf2allelePlot.pl. (Bensasson et al. 2019), which estimates the number of heterozygous point substitutions divided by the length of the high‐quality genome sequence (phred‐scaled quality score over 40). Most strains from trees are homozygous (Figure S1). We removed 35 tree strains that are heterozygous (heterozygosity over 0.001) because they are difficult to represent in downstream phylogenetic analyses and may be interclade hybrids. After filtering, 236 tree‐sampled strains remained for population genetic analyses (Table S1 and Figure S2).

For the reference panel strains, we applied the same quality filtering steps and examined levels of genome‐wide heterozygosity, removing 16 out of 42 published clades because all individuals were heterozygous (Figures S1 and S2).

### 2.4 Population Structure and Genetic Admixture

Whole‐genome alignments were generated by concatenating the alignments for all 16 chromosomes into a single multiple‐alignment file. Strains from trees were compared to reference strains after random selection of three strains per clade from the 26 published clades that remained after quality filtering (Table S2). One strain (BJ6) was randomly assigned to both the CHN‐IV and Far East Asia clades (*N* = 77 strains). Ambiguity codes or lowercase base calls were converted to N's, and ends were filled to align to the same length. A neighbour‐joining tree from genetic distances estimated by pairwise comparison of all genome sequences was constructed using MEGA‐CC (version 10.0.5; Kumar et al. 2012). We used a Tamura‐Nei substitution model (Tamura and Nei 1993) with a gamma distribution and 100 bootstrap replicates. Gaps or missing data were discarded from each pairwise sequence comparison. For visualisation, the neighbour‐joining tree was rotated using ape (version 5.6.2; Paradis and Schliep 2018) and further visualised using ggtree (version 3.4.4; Xu et al. 2021; Yu et al. 2017, 2018).

**FIGURE 1:**
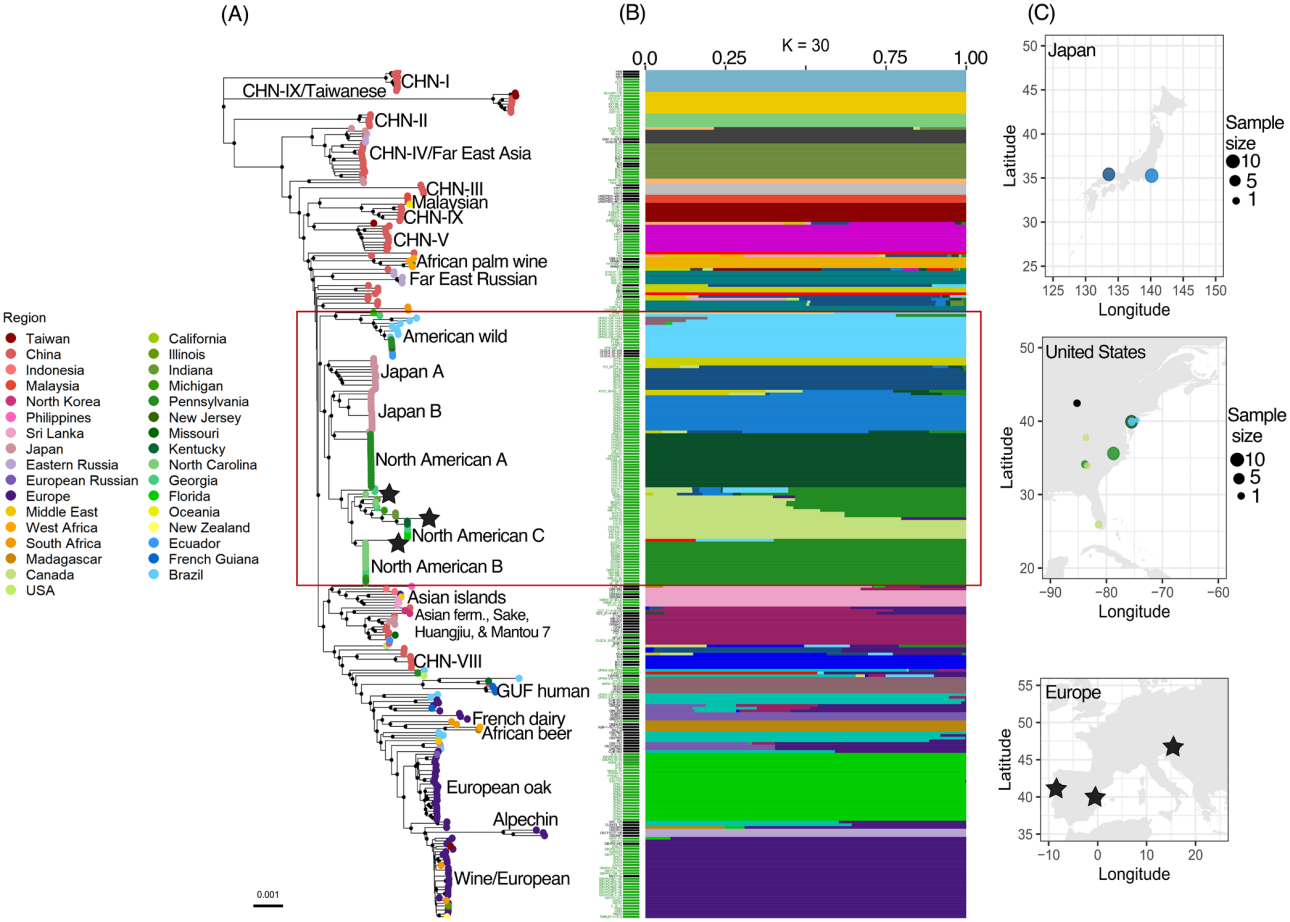
Trees harbour numerous genetically distinct *S. cerevisiae* lineages with population substructure in North America and Japan. (A) Whole‐genome neighbour‐joining tree of 313 strains after excluding heterozygous strains (Table S1). Strains isolated from trees are shown with green text and bars, and reference panel strains with black. Black circles at nodes indicate bootstrap support > 95%. Coloured circles at the tips of the tree show geographical origin. All strains within the red box were isolated from America or Japan except for three European strains (black stars). (B) ADMIXTURE plot with *K* = 30 showing the cluster ancestry proportion for each strain. (C) Maps showing the geographic source of Japanese, North American, and European (black stars in A) tree‐sampled strains from the American and Japanese lineages (red box in A). Circle sizes are based on square‐root‐transformed sample sizes. Japanese and North American strains are colour‐ coded by ancestry from ADMIXTURE plot. We removed North American and Japanese admixed strains (< 90% single‐lineage ancestry) from maps for simplicity.

Population structure and individual ancestry were estimated from SNP allele frequencies using ADMIXTURE (version 1.3.0; Alexander, Novembre, and Lange 2009). Genome data for all strains was merged into a single alignment in variant call format (vcf) using BCFtools, and mitochondrial DNA was removed. Non‐variant sites were filtered out using the min‐ac 1 function in BCFtools, which retains variants with at least one non‐reference allele. Low‐quality reads with a Phred‐scaled quality score under 40 were removed using the minQ option in VCFtools (version 0.1.16; Danecek et al. 2011). Then we converted the single alignment vcf file to text‐formatted and binary files using PLINK (version 1.9; Purcell et al. 2007) for downstream analysis with ADMIXTURE. We assigned strains to distinct populations or genetic clusters (*K*) through repeated runs of ADMIXTURE. Runs assumed different numbers of genetic clusters from 4 to 40 with five replicates per *K*. We selected the run with the highest log likelihood value for each *K* and visualised population structure across different *K*'s (Figure S3). We used CLUMPAK, specifically ‘Distruct,’ to align ancestry proportions (*Q* matrices) across different values of *K* (Kopelman et al. 2015). ADMIXTURE results were visualised as stacked bar plots using the pophelper R package (version 3.2.1; Francis 2017; Figure S4). Distinct genetic clusters were verified if they showed monophyletic clades with at least 95% bootstrap support in a neighbour‐joining tree (Figure 1A and Table S2). We selected the run with the highest number of verified clusters or clades based on monophyletic groups in the neighbour‐joining phylogeny and whether strains grouped by geography.

Phylogenomic relationships among strains were further examined using a maximum likelihood tree after excluding strains that showed possible recent genetic admixture when *K* = 30 (Figure S5 and Table S2). Admixed strains were defined as individuals whose percent ancestry from a single population is less than 90% in ADMIXTURE results for tree‐sampled and reference panel strains (Table S2). It is possible, however, that some of the mixed ancestry invoked by the ADMIXTURE software could reflect ancestral polymorphisms or poorly sampled population subdivision. We included only three reference strains from CHN‐IV and Far East Asia, which appear to be the same clade (Figure 1). We used a genome‐wide alignment of SNPs mapped to the S228c reference genome to construct a phylogenomic tree with IQtree, ultrafast bootstrapping (version 1.6.12) (Minh, Nguyen, and Haeseler 2013; Nguyen et al. 2015), and a general time reversible model with a gamma distribution to estimate site heterogeneity. The maximum likelihood tree was visualised using ape and ggtree in R (version 4.2.2).

### 2.5 Population Substructure Within Europe

Many *S. cerevisiae* strains have good quality genome data from European trees (*N* = 51 strains; Table S3) and have not been tested for population substructure. We used the same methods to analyse population substructure among European tree‐sampled strains. ADMIXTURE was run by varying K from 2 to 8, and each K was repeated five times (Figures S6 and S7). Then, we constructed a phylogenomic tree using maximum likelihood estimation to infer phylogenetic relationships after removing one strain showing mixed ancestry at *K* = 4 (ZP541).

### 2.6 *In Silico* Chromosome Painting

To identify genomic segments that could show gene flow between populations, we used a chromosome painting approach with faChrompaint.pl. and a 30 kb window size (Bensasson et al. 2019). This *in silico* chromosome painting approach compares non‐overlapping sliding windows of sequence to a panel of predefined clades. We identified 25 genetically distinct clades from population structure and phylogenomic analyses (Figures S4 and S5) and randomly selected three strains per clade to use as a “backbone” panel (*N* = 75 backbone strains; Figure S8 and Table S4). For a strain of interest, each 30 kb window was compared to a multiple sequence alignment of backbone strains, then “painted” a colour representing the clade of the most similar backbone sequence. Genomic regions that were diverged from all other backbone sequences (proportion of differing sites over 0.003) were painted white. This divergence threshold was decided based on within‐clade pairwise comparisons for North American and European clades: over 90% of 30 kb windows showed a proportion of differing sites below 0.003 (< 0.3%), and most between‐clade pairwise comparisons showed divergence over 0.003 (Figures S9 and S10). Exclusion of the most diverged regions reduces the chance that genetic similarity between lineages could be the result of incomplete lineage sorting. The genomic regions with low similarity to any other strain are the ones most likely to contain ancestral polymorphisms that have not yet become fixed. There were few such regions (Figure S8).

### 2.7 Time Divergence Analysis

Time divergence analyses were performed on a single non‐admixed locus (30–60 kb) from each backbone strain per chromosome (Figure S11). *In silico* chromosome painting analyses of backbone strains confirmed whether each backbone strain matched its primary clade assignment from allele frequency analyses using ADMIXTURE (Figure S8). For downstream time divergence analyses, we removed three backbone strains with less than 50% primary clade assignment using a chromosome painting approach, five backbone strains with over 10% secondary clade assignment, and only included a single strain from the outgroup CHN‐IX/Taiwanese clade (Table S5).

To estimate the timing of divergence events in the absence of purifying selection, we considered only nucleotide sites at which synonymous changes could occur. We extracted and concatenated 435 genes, with 11–39 genes for each 30–60 kb locus, after excluding 29 genes with introns, 42 genes that overlapped with other genes, and 4 genes with low‐quality sequence for at least 50% of the alignment. Using MEGA‐CC, we extracted 4‐fold and 2‐fold degenerate sites for each locus and concatenated them into 3507 to 19,016 bp alignments with 469–2245 variable and 211–1362 informative sites each. Numbers of informative sites were estimated using SeaView (Gouy, Guindon, and Gascuel 2010).

A neighbour‐joining tree was constructed for each locus on each chromosome using the same methods previously mentioned, and the phylogeny was rooted using the CHN‐IX/Taiwanese strain (EN14S01) as the outgroup. Neighbour‐joining trees and multiple sequence alignments were used to estimate time trees per chromosome using MEGA‐CC with the RelTime‐ML option (Tamura, Tao, and Kumar 2018) using a Tamura–Nei substitution model (Tamura and Nei 1993) with the default setting to consider all sites for branch length calculations (Figure S12).

Using neighbour‐joining distance trees and estimates of the *S. cerevisiae* mutation rate, we estimated the approximate timing at which modern tree‐associated lineages (i) migrated out of Asia; (ii) into North America; and (iii) the separation of Wine/European and European oak lineages from trees. We used genetic distance to estimate the time (T) to the most recent common ancestor (MRCA) in generations per year: *T*_MRCA_ = *k*/*μ*/generations per year, where *k* is the genetic distance to the MRCA of strains in a clade and *μ* is the point mutation rate per bp. We used the mutation rate of 1.67 × 10^−10^ point substitutions per site per generation, which was estimated from hundreds of point mutations after genome sequencing of mutation accumulation lines of diploid *S. cerevisiae* (Zhu et al. 2014). Under controlled laboratory settings at 30°C, wild diploid *S. cerevisiae* and its sister species *Saccharomyces paradoxus* have an average generation (doubling) time of 65 min in the presence of glucose and 125 min on nutrient‐poor growth media (Kaya et al. 2021). Although glucose, fructose, and sucrose are present in the bark of trees that harbour yeast (Sampaio and Gonçalves 2008), it is likely less available than in the lab. In regions where *S. cerevisiae* were easily sampled from trees, historic temperatures were usually below 30°C (Table S8). With these considerations in mind, we made a rough estimate of the number of generations per year for yeast on trees, assuming: (i) a 90 min generation time because while the tree niche is likely less nutrient‐rich than laboratory growth media, some sugars are available; (ii) 12 h of growth per day to account for no growth at lower nighttime temperatures; and (iii) no growth for six months of the year to account for low temperatures in winter. The resulting estimate is an average of 4 generations per day or 1460 generations per year. This is much lower than the number of generations possible at 30°C in nutrient‐rich media in laboratory conditions: 22 per day; 8086 generations per year. It is also lower than the estimate of 2920 generations per year used to estimate the age of the wine‐associated lineage (Fay and Benavides 2005), which may be less affected by cold winters and nutrient‐poor conditions.

## 3 Results

### 3.1 Tree Habitats Harbour Numerous Genetically Distinct Lineages

Using genome data for 236 strains from oaks and other trees, we examined population structure among wild *S. cerevisiae* in this niche. Phylogenetic analyses of tree‐sampled strains and a reference panel of 77 strains from published clades (Duan et al. 2018; Peter et al. 2018) revealed several genetically distinct lineages that only occur on trees from China, Europe, Japan, North America, Russia, and Taiwan (Figure 1, Table S2, and Figure S5). These include previously studied wild lineages such as ‘CHN‐IX,’ ‘CHN‐II,’ and ‘European oak’ (Duan et al. 2018; Peter et al. 2018), and more tree‐associated lineages (see below).

Before applying filters to an initial sample of 328 strains isolated from trees with good genome data (> 30× read depth), we also observed numerous strains from clades that are usually associated with humans (Table S1). For example, there were 29 strains from the ‘Wine/European’ lineage from 21 field sites on 4 continents; 24 from the ‘Asian fermentation’ lineage from 19 sites on 5 continents, 12 ‘South African Beer’ clade strains from 3 South African field sites (Han et al. 2021); 6 strains from the ‘Mixed Origin’ clade associated with baking and clinical strains (Peter et al. 2018); and occasional strains from ‘African honey wine,’ ‘French dairy,’ and ‘African palm wine’ (Table S1). Some of these were too heterozygous for further study (Table S1, Figure S2).

### 3.2 Population Substructure in the Forest Niches of North America and Japan

Phylogenetic analyses further revealed population substructure within North America and Japan, where each region has multiple genetically distinct populations (Figure 1A). Analysis of allele frequencies using ADMIXTURE confirmed that there are several genetically distinct populations in North America and Japan (Figure 1B). More specifically, there are at least four tree‐associated (wild) American *S. cerevisiae* lineages in the eastern United States (Figure 1C) that are well‐supported across phylogenetic and ADMIXTURE analyses (Figure 1, Figures S4 and S5). Most wild strains from Pennsylvania are from a previously described lineage (Liti et al. 2009), and we refer to it here as ‘North American A’ (Tables S1 and S2). There are two more North American wild lineages: ‘North American B’ and ‘North American C’, covering a broader geographical region than the North American A lineage (Figure 1C). North American B strains are from Georgia, North Carolina, a singleton strain from Michigan, and a singleton strain from Pennsylvania (Tables S1 and S2). The North American C lineage occurs in strains from the southeastern United States (Kentucky, Georgia, and Florida). Lastly, wild strains from Ecuador and Brazil cluster with North American strains from Pennsylvania and New Jersey. We are coining this lineage as ‘American wild’ to reflect its Pan‐American geography (Figure 1 and Figure S5) though some of these strains from trees were previously assigned to an ‘Ecuadorian’ clade (Peter et al. 2018) or a ‘Brazil 1’ clade (Barbosa et al. 2016).

Some tree strains have mixed genetic ancestry from deeply diverged American lineages (within the red box in Figure 1A,B), which is expected given their overlapping geographic distributions (Figure 1C). It is perhaps more surprising that these lineages have remained distinct despite gene flow. The strains we and others have sampled are not from primary forests, which have almost vanished from North America (Potapov et al. 2017). A possible explanation is therefore that these forest lineages came into contact only recently because they are newly arrived in their current locations.

In Japan, there are at least two wild *S. cerevisiae* populations: one from Hiruzen Highland, ‘Japan A,’ and one from Chiba Prefecture, ‘Japan B’ (Figure 1 and Figure S5). Japan A is diverged from all North American lineages and Japan B (Figure 1 and Figure S5). Japan B is most like the North American A lineage (Figure 1 and Figure S5).

### 3.3 Fine‐Scale Population Structure of Wild *S. cerevisiae* From Europe

**FIGURE 2:**
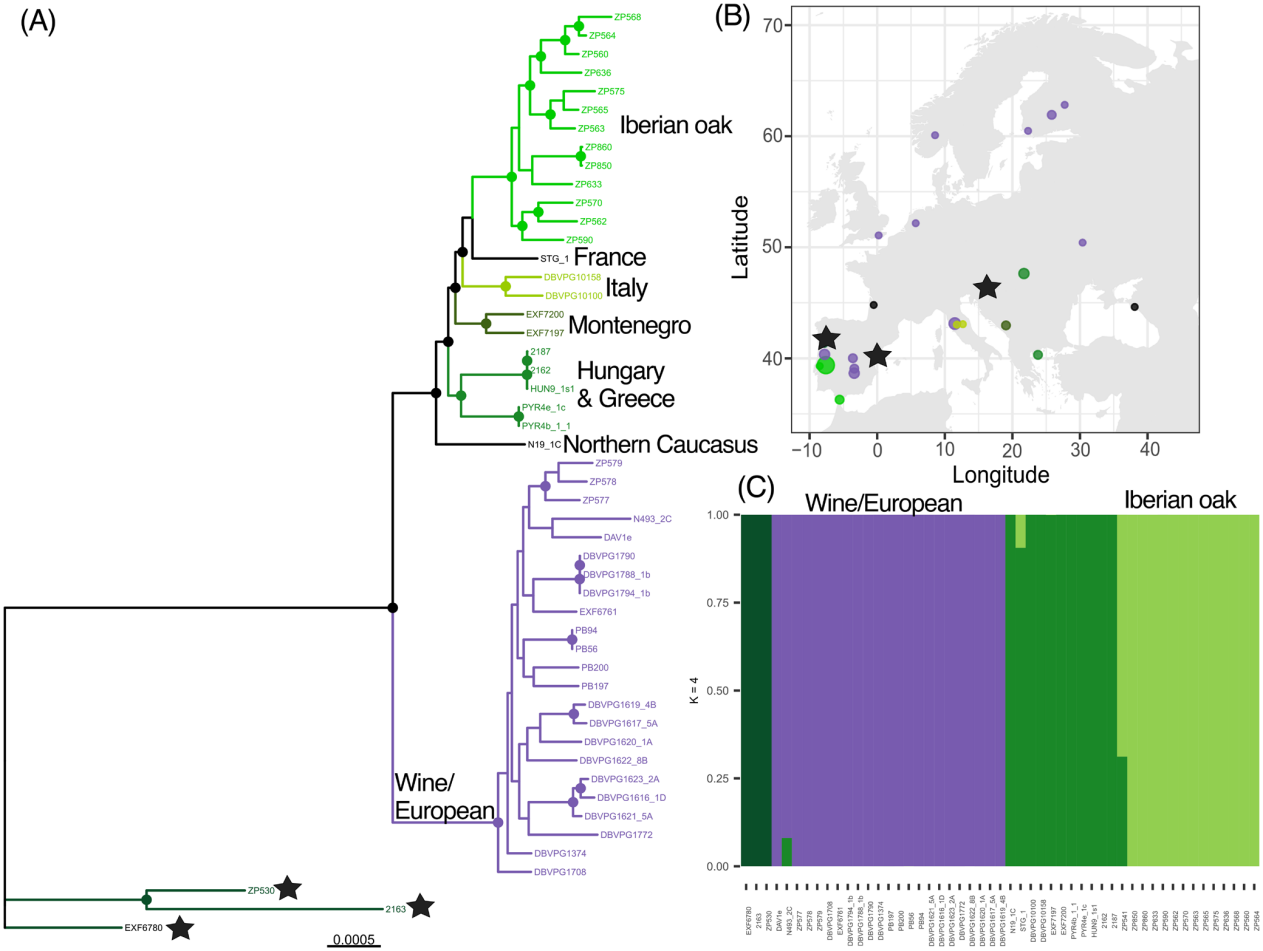
Fine‐scale population structure of wild *S. cerevisiae* from Europe. (A) Whole‐genome maximum likelihood phylogenetic tree of 50 strains after excluding one admixed strain, ZP541, from (C). Black circles at nodes indicate bootstrap support > 95%. Branches are colour‐coded by geography or by ecology. Black stars at tree tips denote strains that genetically cluster with North American strains in Figure 1. (B) Map of Europe showing the geographic source of strains; circles are sized by the square‐root transformed sample sizes and colour‐coded by branch colours in the phylogenetic tree. Singleton strains from France and Northern Caucasus are coloured in black. Black stars denote strains that genetically cluster with North American strains in Figure 1. (C) ADMIXTURE plot when *K* = 4 to examine percent ancestry per individual strain.

European yeast from trees form two genetically distinct lineages: ‘Wine/European’ and ‘European oak’ (Figure 1 and Figure S5) that were previously known (Almeida et al. 2015; Tilakaratna and Bensasson 2017). Although the Wine/European lineage is usually recovered from wine fermentations, it sometimes occurs on trees, especially in vineyards (Gayevskiy, Lee, and Goddard 2016; Hyma and Fay 2013; Robinson, Pinharanda, and Bensasson 2016). Initial analyses suggest population substructure within the European oak lineage (Figure 1 and Figure S4). To better describe this substructure, we therefore ran separate ADMIXTURE and phylogenomic analyses for all strains that were isolated from European trees (*N* = 51; Table S3 and Figure 2). These analyses showed population substructure within the European oak lineage that correlates with geography (Figure 2, Figures S6 and S7). Specifically, there is evidence for five sub‐lineages from: (i) Portugal and Spain, which we are coining ‘Iberian oak’ with support from both phylogenetic and ADMIXTURE analyses (Figure 2); and phylogenetic analyses suggest other distinct populations in (ii) Italy, (iii) Montenegro, (iv) Greece and Hungary, and (v) the North Caucasus (Figure 2). Additionally, several oak trees harbour strains from the ‘Wine/European’ winemaking lineage (Figure 2).

Although there is fine‐scale population structure within the European oak lineage, even the divergence between the European oak and Wine/European lineages is smaller than the divergences seen in Taiwan (Lee et al. 2022), China, Japan, and North America (Figure 1A). The lack of deep divergence among European tree strains (*N* = 53) does not seem due to a lack of sampling because the number of good‐quality genomes is not substantially higher for North America (*N* = 66), and in other continents, deeply diverged lineages can occur in close proximity (Lee et al. 2022; Figure 1C). Instead, the shallow divergences within Europe and within the European oak lineage suggest that *S. cerevisiae* colonised Europe and European forests more recently than in other regions.

### 3.4 Out‐Of‐Asia Migration of Forest Yeast Since the Last Glacial Maximum

**FIGURE 3:**
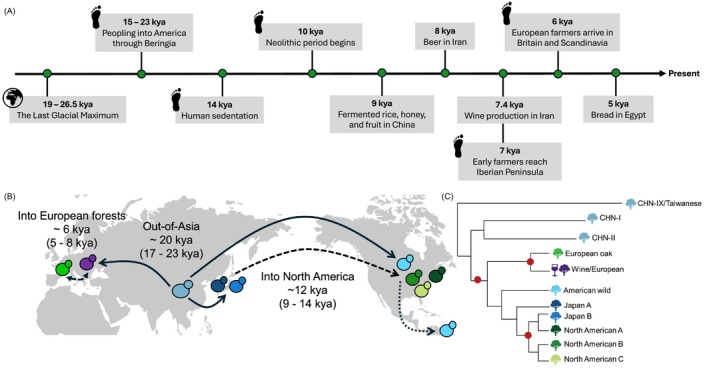
Out‐of‐Asia migration of forest yeast since the Last Ice Age. (A) Timeline showing early archaeological evidence of fermentation and human migration (dates from Clark et al. 2009; Marsit et al. 2017; Nielsen et al. 2017). (B) Map showing forest yeast migration events depicted with a red dot at nodes in (C) (i) out of Asia, (ii) into North America, and (iii) into European forests. Lineages are colour‐coded by the lineages in the cladogram in (C). Dashed arrows indicate secondary migration events. (C) Cladogram showing phylogenetic relationships of lineages of interest for date estimation.

We used a relative rate approach (Tamura, Tao, and Kumar 2018) to estimate the timing of lineage divergences that likely correspond to (i) migration events out of Asia, (ii) into North America, and (iii) the origin of a Wine lineage distinct from European forest lineages. After excluding admixture, phylogenetic analysis of individual loci (30–60 kb) from each chromosome reproduced most genetically distinct clades defined in this study (Table S6 and Figure S12). Using these phylogenetic trees and assuming a mutation rate of 1.67 × 10^−10^ point substitutions per site per generation (Zhu et al. 2014) and 4 generations per day (see Section 2), we generated rough estimates of divergence times. East Asia is the probable origin for *S. cerevisiae* (Duan et al. 2018; Han et al. 2021; Wang et al. 2012), and we estimate that non‐Asian lineages diverged from those only found in Asia approximately 20 thousand years ago (mean = 19.8 kya, 95% CI 16.9–22.7 kya; Figure 3). Out of 16 individual loci, 14 loci showed a clade for European oak (Table S7 and Figure S12). Time divergence estimation for these loci suggests European oak and the domesticated Wine/European lineage diverged around 6 kya (mean = 6.1 kya, 95% CI 4.5–7.6 kya; Figure 3). Out of 16 loci, 9 loci clustered the 3 North American clades (A–C), sometimes with Japan B (5 loci) or Japan A lineages (2 loci). This mostly North American clade appears to diverge from others (Table S7 and Figure S12) around 12 kya (mean = 11.6 kya, 95% CI 9.4–13.7 kya; Figure 3). These estimates suggest global *S. cerevisiae* migrations occurred since the last glacial maximum (Figure 3).

### 3.5 Occasional Strains in Europe Resemble Present‐Day North American Lineages

**FIGURE 4:**
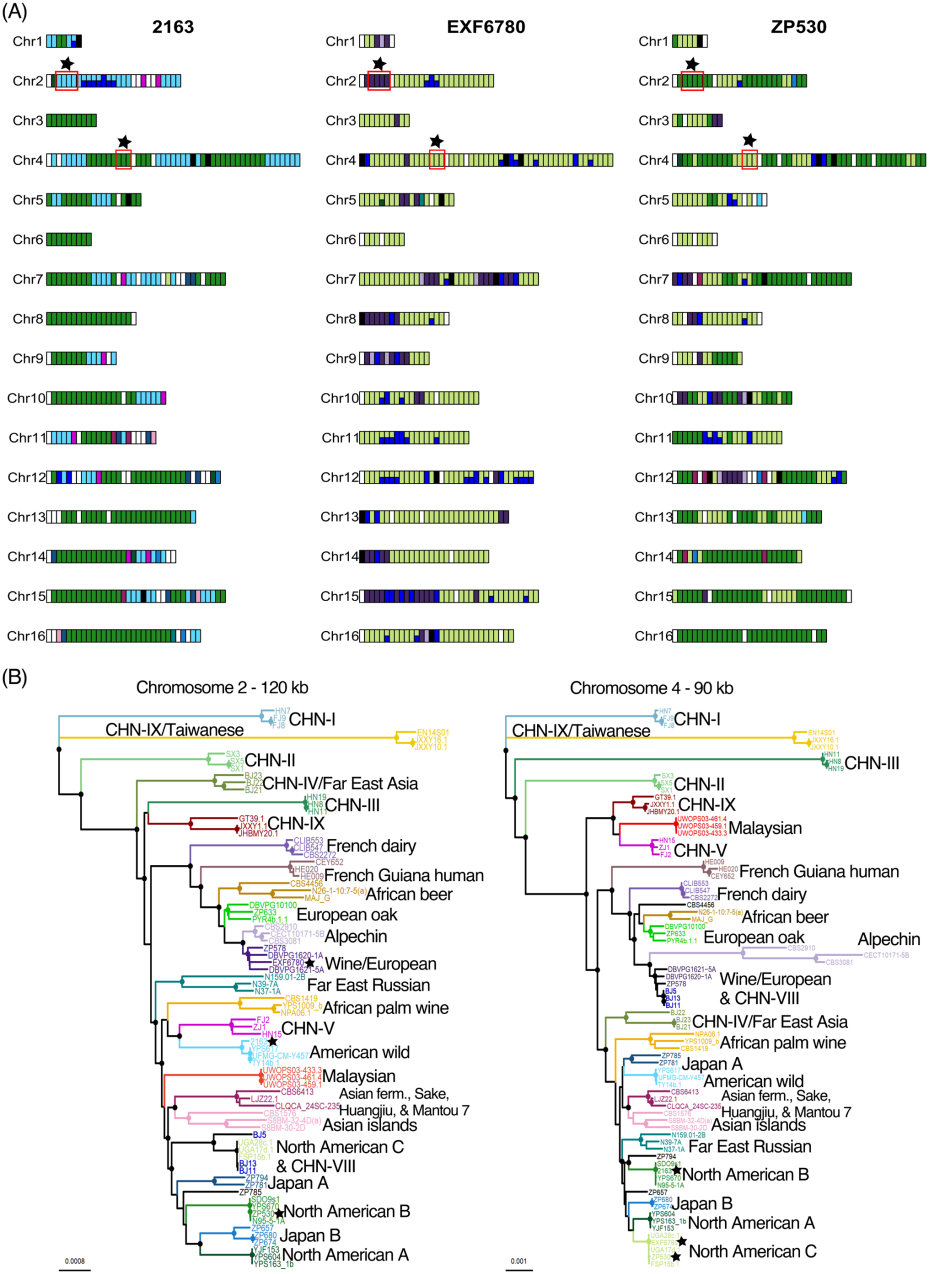
Occasional strains in Europe resemble present‐day North American lineages. (A) Painted chromosomes of 2163, EXF6780, and ZP530 show admixture between multiple lineages. Genomic regions were “painted” based on the clade assignment of the most similar strain in 30 kb non‐overlapping windows. Diverged regions were not coloured (white) and were defined as regions that differed by 0.003 from all other strains in the backbone phylogeny. Black‐coloured regions indicate low coverage. Colours are as in (B): North American B is forest green, American wild is light blue, North American C is light green, Wine/European is dark purple, and CHN‐VIII is blue. Genomic regions (90–120 kb) were selected for phylogenetic analysis (red boxes with black stars). (B) Neighbour‐joining phylogenetic trees for two loci. Solid circles at nodes indicate bootstrap support > 95%. Branches are colour‐coded by clade. Phylogenetic analyses show that in the absence of admixture, 2163, EXF6780, and ZP530 (black stars) are very similar to strains from American wild, Wine/European, North American B, and C.

There are three strains from European trees (ZP530, 2163, and EXF6780) that resemble North American strains (black stars in Figure 1) and differ from all other European lineages (Figure 2). These genomes are from two different investigations where (i) ZP530 was isolated from chestnut ( *Castanea sativa* ) from Marão, Campeã, Portugal (Almeida et al. 2015), (ii) EXF6780 was isolated from sessile oak ( *Quercus petraea* ) from Velike Lašče, Kobila hill, Slovenia (Almeida et al. 2015), and (iii) 2163 was isolated from Portuguese oak (*Quercus faginea*) from Castellon, Spain (Peter et al. 2018). In the phylogenetic analysis, these strains differ from their most closely related clades (North American B and C in Figure 1A) and appear to show some genetic admixture (Figure 1B). Did these strains arrive on European trees as a result of ancient migration, or could their genetic distance from other North American strains be explained by recent admixture? To find out, we “painted” their chromosomes according to the clade of the most closely related strain (Figure 4A). EXF6780, ZP530, and 2163 were compared to our backbone phylogeny (Table S5), which revealed the strains are a mix of three lineages found in North America and that two strains have admixture from the lineage used to ferment grape wine (Figure 4).

To assess genetic distances from modern North American lineages while accounting for admixture, we selected genomic regions from chromosomes 2 and 4 for phylogenetic analysis that did not show admixture from multiple clades (Figure 4A). Phylogenies with the effects of admixture removed in this way showed that strains 2163, EXF6780, and ZP530 seem almost identical to modern North American lineages across (Figure 4B). It therefore seems most likely that these ‘American European’ strains arrived very recently in Europe and that admixture among North American and Wine/European lineages (Figure 4) explains their genetic distance from modern strains in the whole‐genome phylogeny (Figure 1A).

Intriguingly, all three of these American European strains show some regions with close sequence similarity to CHN‐VIII (blue windows in Figure 4A). One potential explanation is that the CHN‐VIII lineage also has some recent domesticated Wine/European ancestry because all reference Wine/European strains resemble CHN‐VIII in many genomic regions (Figure S8). Chromosome painting shows that one of the 3 strains representing CHN‐VIII is very similar to reference strains from North American C, American Wild, and Wine/European lineages (> 10%, Figure S8). Locus‐by‐locus phylogenetic analysis (Figure S11) suggests that the remaining two CHN‐VIII strains are diverged from the European oak lineage, yet indistinguishable in large genomic regions from the Wine/European lineage (chromosomes 4–7 and 11) and North American C (chromosomes 2 and 12). CHN‐VIII genomes are also similar to the American wild lineage (chromosomes 3 and 15) and show regions diverged from any other lineages (chromosomes 1, 8, 9, and 16). The degree of similarity between modern Wine/European and North American C strains suggests very recent gene flow from Europe. Consistent with this proposal, CHN‐VIII occurs in apple orchards and secondary forests near Beijing, and Wine/European strains have also been isolated from orchards in China (Duan et al. 2018; Wang et al. 2012).

## 4 Discussion

### 4.1 Genetic Isolation in Forest Niches

Before molecular methods showed that *S. cerevisiae* lives on trees (Naumov et al. 1992; Sniegowski, Dombrowski, and Fingerman 2002), people thought it lived only with humans and not in natural environments (Vaughan‐Martini and Martini 1995). This resembles thinking for *Candida* pathogenic species before their recent discovery on trees and other plants (Bensasson et al. 2019; Opulente et al. 2019). Yet our results suggest that *S. cerevisiae* recently colonised woodlands many times and lived there in relative isolation from humans. Phylogeographic analysis of forest *S. cerevisiae* populations shows that strains are recognisably from Iberian, French, or Italian trees, with further lineages occurring in Eastern Europe (Figures 1 and 2). There are at least four forest lineages in North America with regional differences; for example, North American C occurs in the southern USA, and North American A in Pennsylvania. The higher divergence seen among North American lineages (A‐C) suggests that their most recent common ancestor must considerably predate the common ancestor of European *S. cerevisiae* and therefore an earlier arrival (see below). The genetic similarity of North American and Japanese lineages (A and B) suggests that the migration into North America, after the earlier arrival of American Wild, came from a different Asian source, perhaps close to Japan. There is also genetic (mtDNA) and archaeological evidence suggesting shared ancestry among the people of China, Japan, and the Americas, suggesting a human migration route from northern coastal China into the Americas (Li et al. 2023).

Past analyses also show high population structure in *S. cerevisiae* from the primaeval forests of East Asia (Wang et al. 2012; Lee et al. 2022) and in its sister species, *S. paradoxus* (Hénault et al. 2019; Leducq et al. 2014). *Saccharomyces* yeast are probably not usually air‐dispersed (Mortimer 2000); therefore, it is not surprising that they show more population structure than other fungal microbes. Our observations for the tree niche contrast with the broader distribution of domesticated and fruit‐associated lineages (Almeida et al. 2015; Duan et al. 2018; Gallone et al. 2016; Gonçalves et al. 2016; Lee et al. 2022; Legras et al. 2007, 2018; Peter et al. 2018) and are consistent with the proposal that animal‐assisted long‐distance migration is relatively rare in forests (Magwene et al. 2011; Tilakaratna and Bensasson 2017).

The evidence for isolated forest populations includes phylogenetic analyses using whole genomes, single chromosomes, and single locus data (Figures 1A, 2A, 4B, Figures S5 and S12) in addition to analyses of allele frequencies (Figures 1B and 2C, Figure S7). Monophyletic tree‐associated clades were reproducible across most chromosomes at the tips of phylogenetic trees, suggesting the fixation of many alleles for each lineage (Figures 1A, 2A, 4B, Figures S5 and S12). Why might *S. cerevisiae* show many phylogenetically distinct lineages within continents? According to past estimates, *S. cerevisiae* reproduces sexually only once in hundreds or thousands of generations (Magwene et al. 2011; Lee et al. 2022), and the same is true for *S. paradoxus* (Tsai et al. 2008). Even when meiosis does occur, *S. paradoxus* are almost always selfing (99% of sexual cycles, Tsai et al. 2008). Asexual reproduction of *S. cerevisiae* might lead to population bottlenecks and the local fixation of alleles by genetic drift.

### 4.2 Global Spread of *S. cerevisiae* Forest Populations Since the Last Glacial Maximum

Mutation rate estimates for *S. cerevisiae* applied to phylogenetic analyses suggest that the expansion of forest *S. cerevisiae* out of Asia likely occurred in the last 20,000 years (Figure 3 and Figure S12). Divergence among the forest lineages of Europe and America is less deep than among the lineages of Asia (Figure 1A), so our analyses support an East Asian species origin (Wang et al. 2012; Lee et al. 2022) with the caveat that better sampling in other regions such as Central Asia or Central Africa could also reveal high genetic diversity. Averages estimated from phylogenies of 16 loci suggest that *S. cerevisiae* lineages migrated out of Asia around 17–23 kya (Figure 3 and Figure S12), which was when climate started warming after the last glacial maximum (Clark et al. 2009). The forest lineages that first diverged from East Asian populations include those occurring in South America (American wild, French Guiana human; Figure S12). The fine‐scale population structure within North American forests arose more recently; since the divergence of North American lineages (A‐C) from most Asian lineages around 9–14 kya. The population structure occurring on European trees arose since these lineages diverged from the Wine lineage approximately 5–8 kya.

The timing of these yeast migrations seems to roughly coincide with human migration into America (15–23 kya), sedentarization (~14 kya), widespread settlements in the Americas (12.6–13 kya), the origins of agriculture (~10 kya), fermentation practices in Asia (~9 kya), European agriculture (6–7 kya), and European winemaking (~4 kya; Figure 3A; Marsit et al. 2017; McGovern 2003; Nielsen et al. 2017;). It is therefore possible that humans or their commensals carried yeast with their food as they moved around the world. The alternative, that yeast migrated naturally across the globe as the climate warmed, seems less likely for multiple reasons: (i) *S. cerevisiae* are only rarely isolated from trees in northern Europe, and upon further genetic investigation, northern populations appear feral or domesticated (the Wine/European lineage or admixed; Robinson, Pinharanda, and Bensasson 2016). Tree‐associated genetic lineages of *S. cerevisiae* have not been reported from northern cool temperate regions such as Canada or eastern Russia, despite documentation of other *Saccharomyces* species (Charron, Leducq, and Landry 2014; Naumov et al. 2000). Indeed, tree‐associated genetic lineages may have a mostly subtropical or tropical distribution (Robinson, Pinharanda, and Bensasson 2016). A natural expansion through the cold climate of the Bering land bridge therefore seems unlikely. (ii) Such an expansion seems more likely for *S. paradoxus,* which inhabits northern forests in Canada, northern Europe, and Siberia, yet that species shows much greater genetic isolation between America, Asia, and Europe (Liti et al. 2009), perhaps because it does not live in association with humans. (iii) *S. cerevisiae* differs from *S. paradoxus* in that it is common on fruit. Humans might have inadvertently carried *S. cerevisiae* with their food, vessels, or the insects that travelled with them. (iv) Human‐associated migration can explain a relatively recent origin of European lineages that is roughly coincident with the expansion of agriculture into Europe (5–8 kya), the much later spread to New Zealand in the last 1000 years, and their concentration near New Zealand vineyards (Gayevskiy, Lee, and Goddard 2016). A less likely possibility is that *S. cerevisiae* was naturally dispersed by insects capable of migrating from warm regions in Asia to North America. Monarch butterflies have dispersed from America to Australia via Pacific islands since the last glacial maximum (Zhan et al. 2014). Such long‐distance dispersal is rare, however, even for known long‐distance dispersers like locusts, which do not have cosmopolitan distributions (Lovejoy et al. 2005) like those of humans or other human commensal organisms.

Our time estimates are based on the well‐studied mutation rate of *S. cerevisiae* . The estimate we use (1.67 × 10^−10^ per base per generation) is the most accurate, from hundreds of point mutations (867) observed in 145 genome sequences from diploid mutation accumulation lines (Zhu et al. 2014). Earlier mutation rate estimates did not differ greatly: 2.9 × 10^−10^ from mutations accumulated in a different diploid background (Nishant et al. 2010), 3.3 × 10^−10^ in haploids (Lynch et al. 2008), or 1.84 × 10^−10^ from reporter assays in haploid strains at individual loci (Drake 1991; Fay and Benavides 2005). The number of generations occurring in natural forest environments is more difficult to measure (Mozzachiodi et al. 2022). As in past analysis of wine yeast by Fay and Benavides (ca. 2920 generations per year; 2005), we assume a lower growth rate than in the laboratory and only 12 h of growth per day. The rate we use for trees (ca. 1460 generations per year) assumes slower growth because of fewer nutrients on trees and no growth for 6 months of the year because of low temperatures (see Methods). For some parts of the species range, such as Florida and Georgia, generation times could be underestimated because some of the maximum temperatures in the coldest 6 months (18°C–29°C) and minimum nighttime temperatures in the hottest months (16°C–23°C) are also high enough for yeast growth (Table S8; Sweeney, Kuehne, and Sniegowski 2004). While the fossil record is excellent for estimating divergences among yeast families or genera (Douzery et al. 2004; Marcet‐Houben and Gabaldón 2015; Shen et al. 2018), the most recent fossil for ascomycotans dates to 417 million years ago (Douzery et al. 2004) and therefore may not be accurate for intraspecies divergences. Our timings suggest an older divergence (17—23kya) than that of Fay and Benavides (2005) for the split of wine and East Asian sake strains (11.9 kya), and are consistent with past estimates for divergence among wine strains (3.7 kya; Fay and Benavides 2005), the split between European oak and wine 10.3–1.3 kya (Almeida et al. 2015), and the arrival of *S. cerevisiae* in New Zealand less than 1 kya (Gayevskiy, Lee, and Goddard 2016).

### 4.3 Ongoing Migration Between Human and Tree Environments

Not all *S. cerevisiae* strains on trees are from tree‐associated lineages. Strains from the European grape wine lineage and other human‐associated lineages also live on trees (Gayevskiy, Lee, and Goddard 2016; Hyma and Fay 2013; Robinson, Pinharanda, and Bensasson 2016). Indeed, there were at least 10 migration events from Europe to New Zealand trees that happened in the last 1000 years since humans arrived in New Zealand (Gayevskiy, Lee, and Goddard 2016). Here we observe strains on trees from clades connected with grape and African wines, Asian fermentations, brewing, baking, and clinical strains (Table S1, Figure S2), which suggests transmission between humans and trees is ongoing.

### 4.4 Footprints of Human Activity in the Genomes of European Tree Strains

Three strains from trees in Portugal, Spain, and Slovenia had genome sequences that were predominantly from North American forest clades. Chromosome painting of their genomes shows these strains must be descended from three different transatlantic migrants (Figure 4A). These migrations from North America must have been recent because analysis of large (90 and 120 kb) loci shows genomic tracts that look typical of current North American B, C, and American wild lineages (Figure 4B). None of these American European strains resemble strains from the North American A lineage, which only occurred in Pennsylvania (Table S1). Interestingly, strains from Portugal and Slovenia (ZP530 and EXF6780) resemble the North American C lineage (Figure 4), which we only observe in the southern USA (Figure 1C).

A potential explanation for the presence of North American tree‐associated lineages in Europe is the human response to the Great French Wine Blight. In the 1850s, humans accidentally introduced an insect pest, *Phylloxera*, from North America to Europe that destroyed most European vineyards. Native American vines are naturally resistant to *Phylloxera*. The European wine industry was rescued by the mass import of vines from the southern USA to Europe, and from the late 1800s to the present day, most European vines are grafted onto resistant North American grapevine rootstock (Campbell 2004). The imported American grapevines could have harboured North American yeast. In support of an explanation, Portuguese and Slovenian American strains show admixture from the Wine/European lineage, suggesting recent association with vineyards, whereas European oak and North American forest lineages rarely show admixture from the wine lineage (Figure 4). The puzzling occurrence of long stretches of Wine/European and North American DNA in yeast strains from orchards and secondary forests near Beijing in China could then be explained by very recent human‐assisted migration from Europe.

## 5 Conclusion

In summary, our analyses show forests harbour many isolated *S. cerevisiae* populations that are distinct from human‐associated lineages. The phylogeographic structure of tree‐associated lineages implies that migrants from humans rarely establish in forest niches. Yet even rare events can shape the distribution of a species. The postglacial spread of forest *S. cerevisiae* out of Asia and into North America and Europe suggests that this substantial impact was driven by people. Consistent with this, we also observe footprints of ongoing human‐assisted movement of forest yeast. Fungal microbes introduced into forests can transform landscapes when they are mutualists or parasites (Hoeksema et al. 2020; Averill et al. 2022). For intimate human commensals and occasional pathogens, such as *S. cerevisiae* and *Candida sp*., it seems important to consider their evolution in non‐human environments—especially since environmental fungal microbes may adapt to fungicide use or rising temperatures (Garcia‐Solache and Casadevall 2010; Leducq et al. 2014; Kang et al. 2022; Lockhart, Chowdhary, and Gold 2023).

## Author Contributions

The research was conceptualised and designed by J.J.P. and D.B. A.K.W., J.J.P., and E.F.C.S. performed DNA extractions for genome sequencing. J.J.P. and E.F.C.S. developed bioinformatic pipelines and obtained and curated the data. J.J.P. performed analyses and data visualisation. J.J.P. and D.B. wrote the paper with input from E.F.C.S. and A.K.W.

## 6 Conflicts of Interest

The authors declare no conflicts of interest.

## Supporting information


Figure S1

Table S1

## Data Availability

Short‐read genome data are available in the NCBI‐SRA under project number PRJNA1090965. Consensus genome sequences generated by mapping to the sacCer3 reference, alignments, and PLINK files used in ADMIXTURE analyses are available on Dryad under https://doi.org/10.5061/dryad.pnvx0k6zq. Benefits from this research include this sharing of our data and results on public databases and the sharing of the yeast strains isolated for this work, which are available from our lab or public yeast collections.
