# Supplementary material for: Footprints of Human Migration in the Population Structure of Wild Baker's Yeast
Source: Mol Ecol. 2025 Feb 4;34(20):e17669. doi: 10.1111/mec.17669 (PMC12530295; doi:10.1111/mec.17669)
Supplement: Supplementary file 1 — Figure S1 [file MEC-34-e17669-s002.pdf]

MOLECULAR ECOLOGY

Supplemental Information for:

Footprints of human migration in the population structure of wild baker's yeast

Jacqueline J. Peña, Eduardo FC Scopel, Audrey K. Ward and Douda Bensasson

Table of Contents:

**Supplemental figure 1**	Page 2
**Supplemental figure 2**	Page 3
**Supplemental figure 3**	Page 4
**Supplemental figure 4**	Page 5
**Supplemental figure 5**	Page 7
**Supplemental figure 6**	Page 8
**Supplemental figure 7**	Page 9
**Supplemental figure 8**	Page 10
**Supplemental figure 9**	Page 19
**Supplemental figure 10**	Page 20
**Supplemental figure 11**	Page 21
**Supplemental figure 12**	Page 28

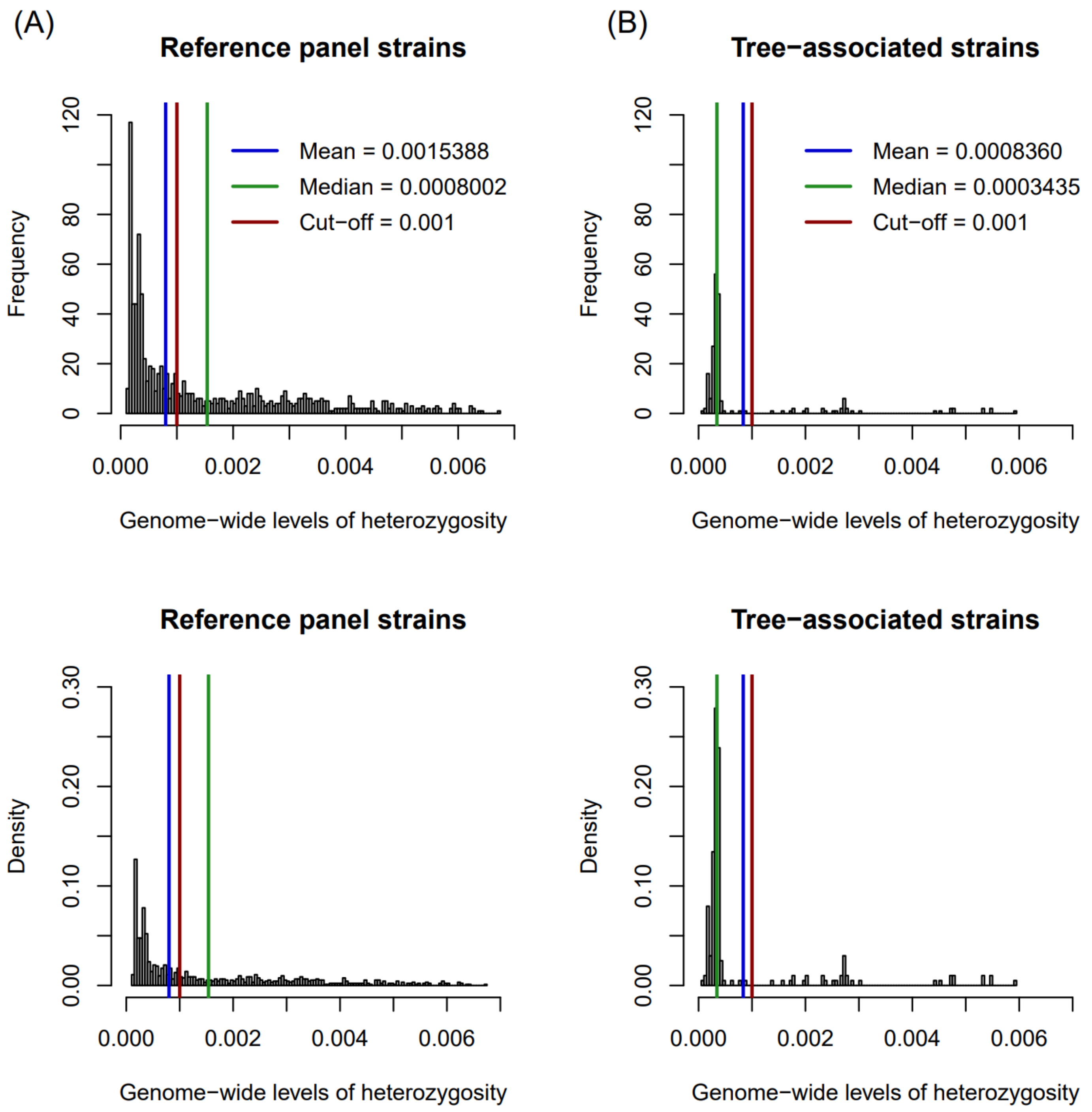

Supplemental Figure 1 Genome-wide levels of heterozygosity of Saccharomyces cerevisiae partitioned by tree-associated (N = 172) (A) and non-tree-associated strains (N = 881) (B) after excluding monosporic derivatives. For downstream population structure analyses, we applied a 0.001 heterozygosity cutoff (red vertical line) to exclude strains that are likely to be inter-clade hybrids, which would obscure phylogenetic relationships.

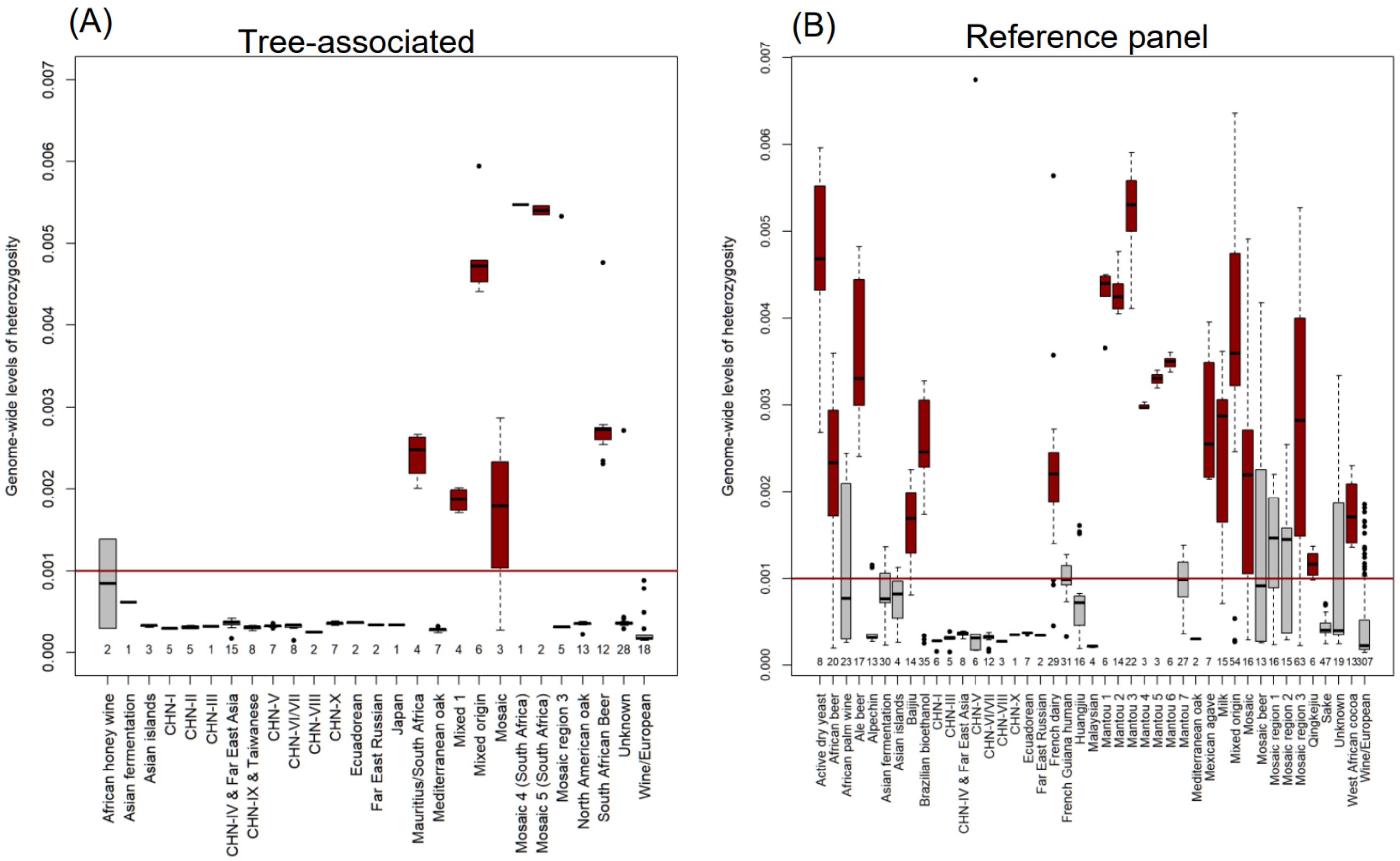

Supplemental Figure 2 Genome-wide levels of heterozygosity for Saccharomyces cerevisiae of tree-associated (N = 172) (A) and non-tree-associated strains (N = 881) (B) partition by published clades after excluding monosporic derivatives. Heterozygous clades are highlighted in red where 90% or more of the strains within that clade have a genome-wide level of heterozygosity greater than 0.001 (horizontal red line). Numbers on the plots indicate the number of strains per clade. Heterozygous clades are prevalent in non-tree-associated clades (N = 37) compared to tree-associated clades (N = 21) (Fisher's exact test, P = 0.04). For statistical analyses, we excluded published clades that were defined as 'mosaic clades' (i.e. Mosaic region 1) because these lineages represent multiple source populations (N = 5 tree-associated clades excluded and N = 3 non-tree-associated clades) and strains that have an unknown lineage. A reference panel of non-tree-associated strains from published clades was created by randomly selecting three strains per homozygous clade (highlighted in gray) to see how tree-associated strains genetically cluster with non-tree associated strains. For example, all the strains within the Active dry yeast lineage are heterozygous and thus this lineage is not represented in the reference panel because heterozygous strains are likely intra-clade hybrids, which would obscure phylogenetic relationships.

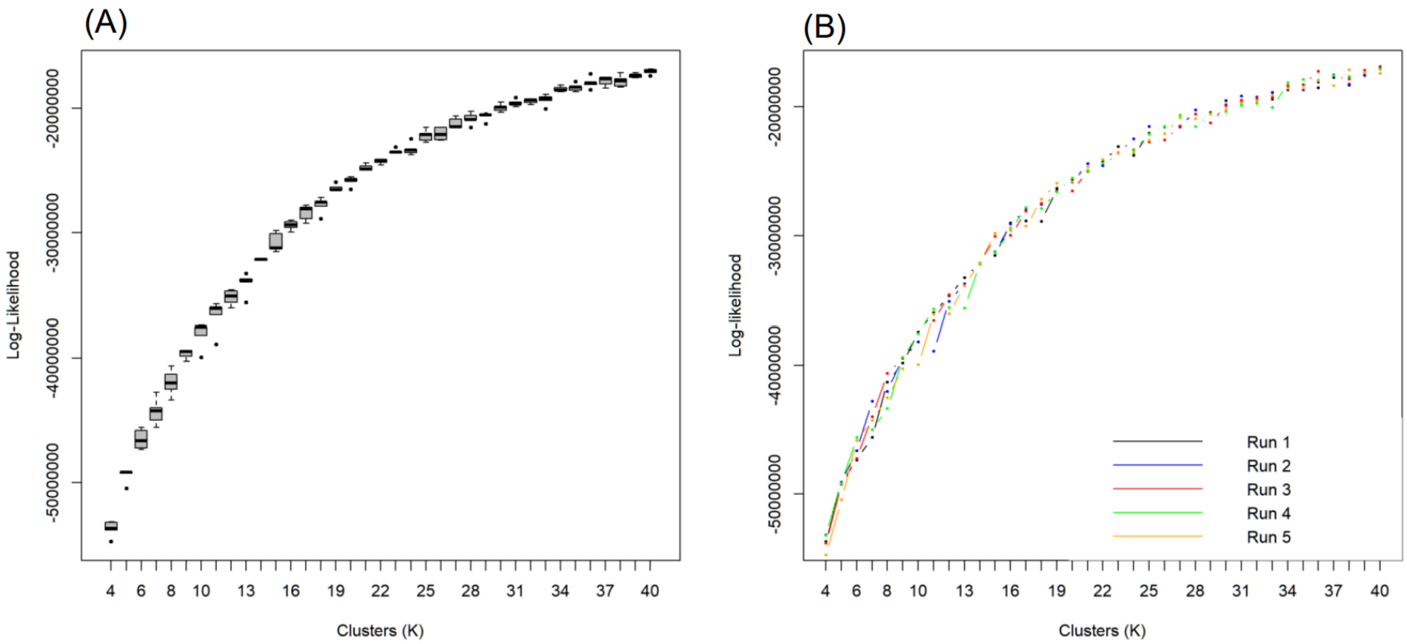

Supplemental Figure 3 Loglikelihood values from ADMIXTURE analyses for each cluster (K) across five replicate runs among 313 S. cerevisiae tree-associated and reference panel strains (non-tree-associated) strains. (A) The loglikelihood values as a function of five replicate runs. (B) The loglikelihood values for each replicate run across Ks.

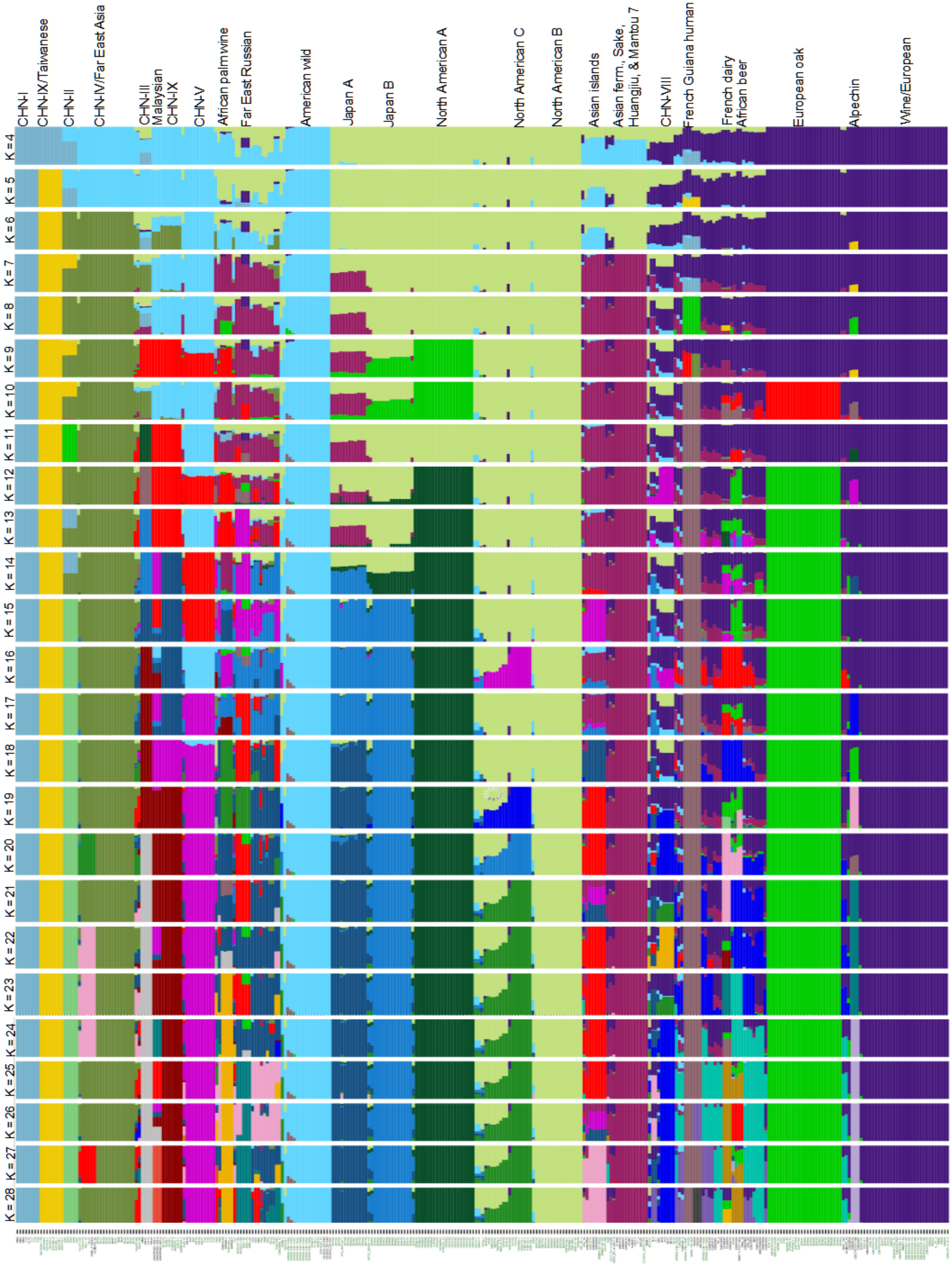
CHN-I
CHN-IX/Taiwanese
CHN-II
CHN-IV/Far East Asia
CHN-III
Malaysian
CHN-IX
CHN-V
African palm wine
Far East Russian
American wild
Japan A
Japan B
North American A
North American C
North American B
Asian islands
Asian ferm., Sake,
Huangjiu, & Mantou 7
CHN-VIII
French Guiana human
French dairy
African beer
European oak
Alpechin
Wine/European
K = 4
K = 5
K = 6
K = 7
K = 8
K = 9
K = 10
K = 11
K = 12
K = 13
K = 14
K = 15
K = 16
K = 17
K = 18
K = 19
K = 20
K = 21
K = 22
K = 23
K = 24
K = 25
K = 26
K = 27
K = 28

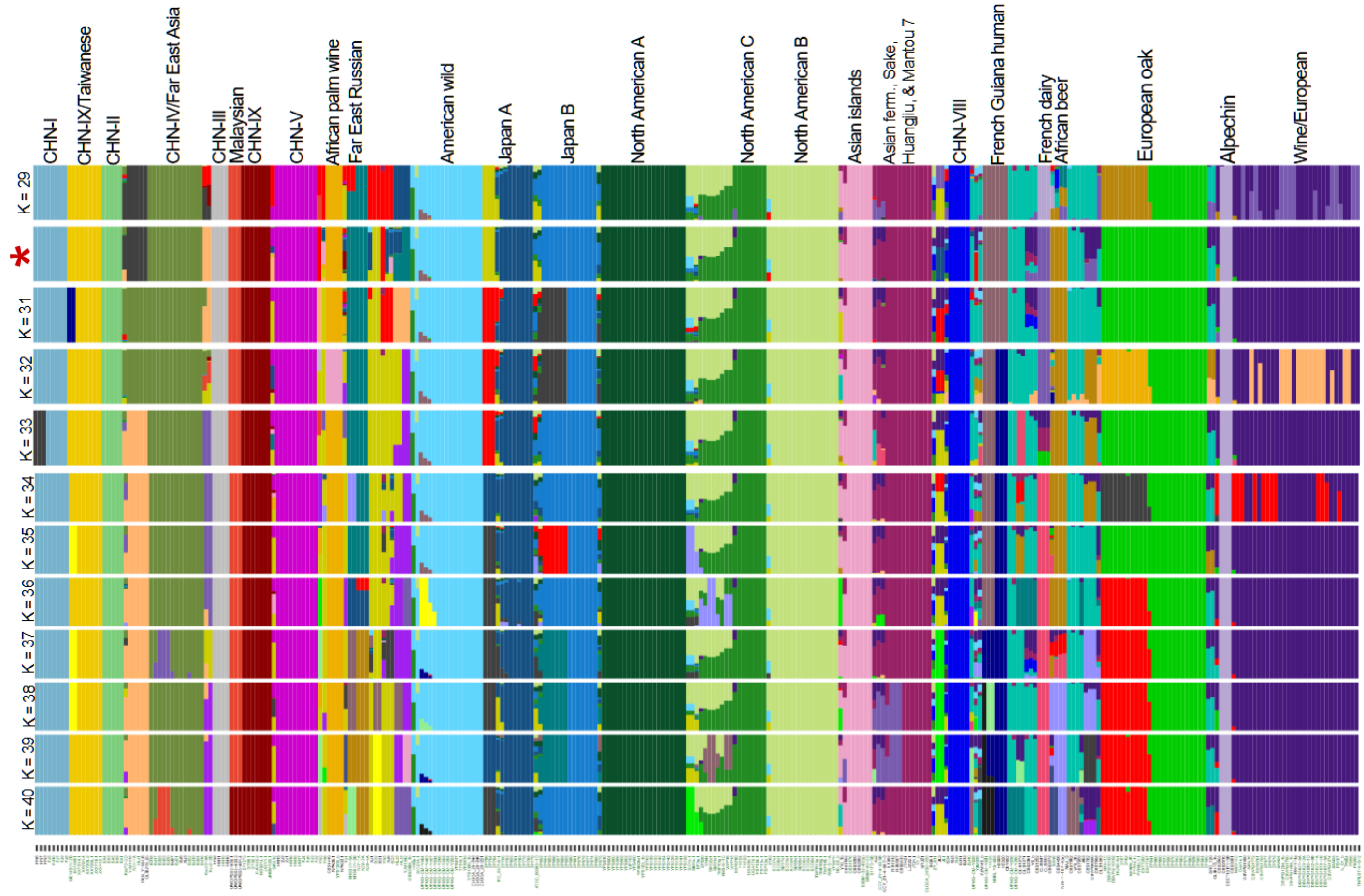

Supplemental Figure 4 Population structure and admixture of *Saccharomyces cerevisiae* tree-sampled strains and reference panel strains after excluding strains with heterozygosity > 0.001. Lineages were estimated using ADMIXTURE from varying cluster (K) 4-40 with five replicate runs per each K. We selected the run that had the highest loglikelihood value from each K and strains are ordered by their position in the neighbor-joining tree. Lineages that only have tree-sampled strains are highlighted in green text. The ADMIXTURE plot highlighted with a red asterisk (K = 30) is the model that had distinct genetic clusters that matched monophyletic clades in the neighbor-joining tree (>95% bootstrap support).

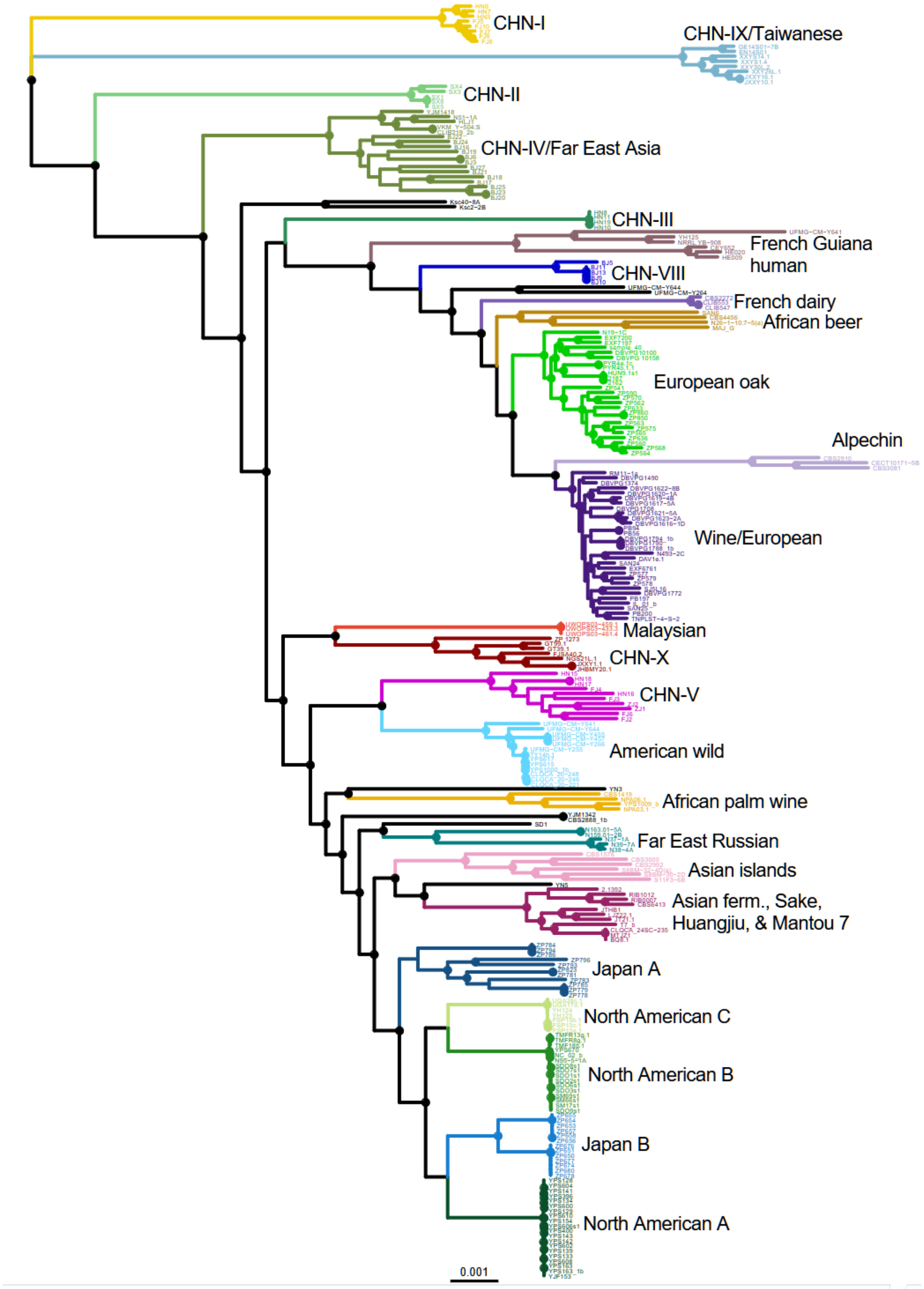

Supplemental Figure 5 Phylogenetic relationships of Saccharomyces cerevisiae. Whole-genome phylogeny of tree-sampled and reference panel strains after excluding admixed strains (percent ancestry to a single lineage is <90% when K = 30; Figure 1B). The phylogeny was constructed using maximum likelihood estimation using IQtree ultrafast bootstrapping (1000 bootstraps) using a general-time reversible model with a gamma distribution. Filled circles at nodes show monophyletic clades with >99% bootstrap support.

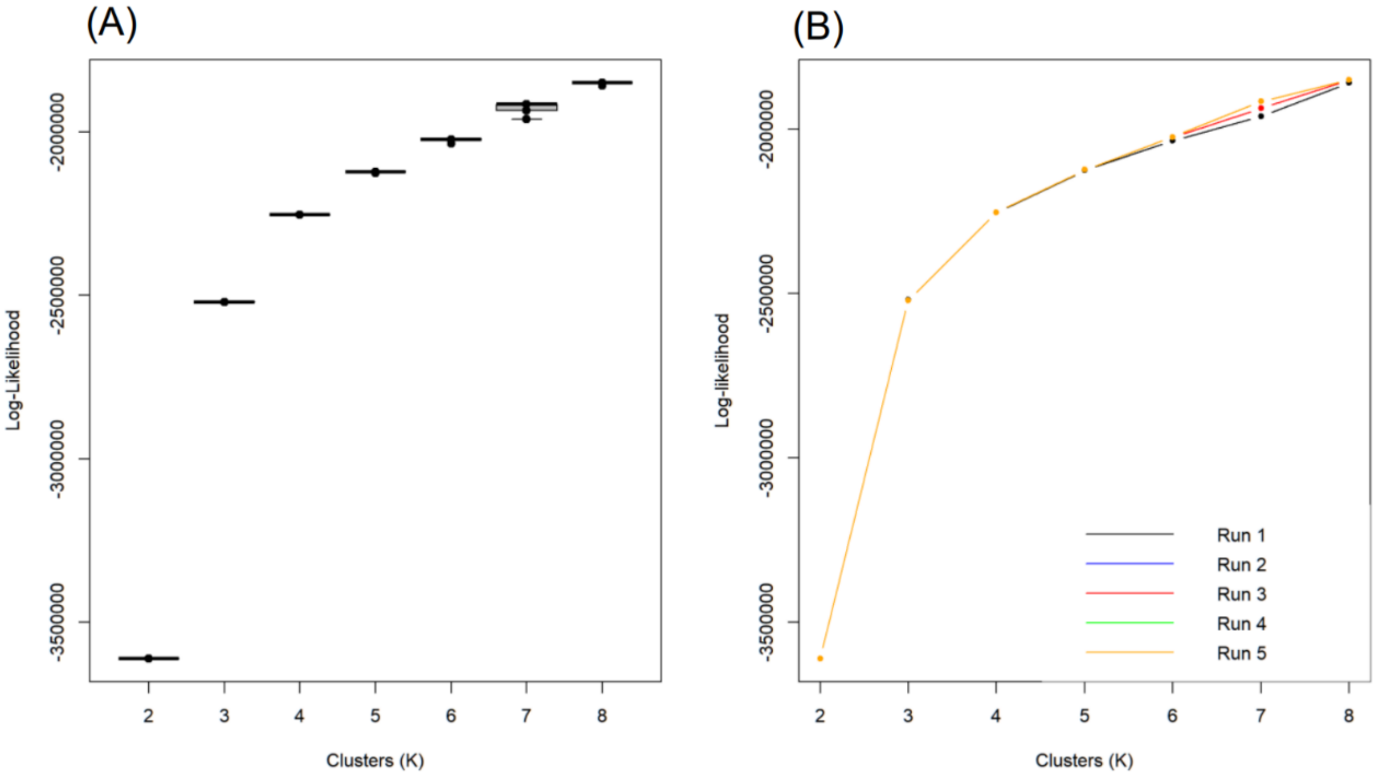

Supplemental Figure 6 Loglikelihood values from ADMIXTURE analyses for each cluster (K) across five replicate runs among 51 wild S. cerevisiae from European woodlands. (A) The loglikelihood values as a function of five replicate runs. (B) The loglikelihood values for each replicate run across Ks.

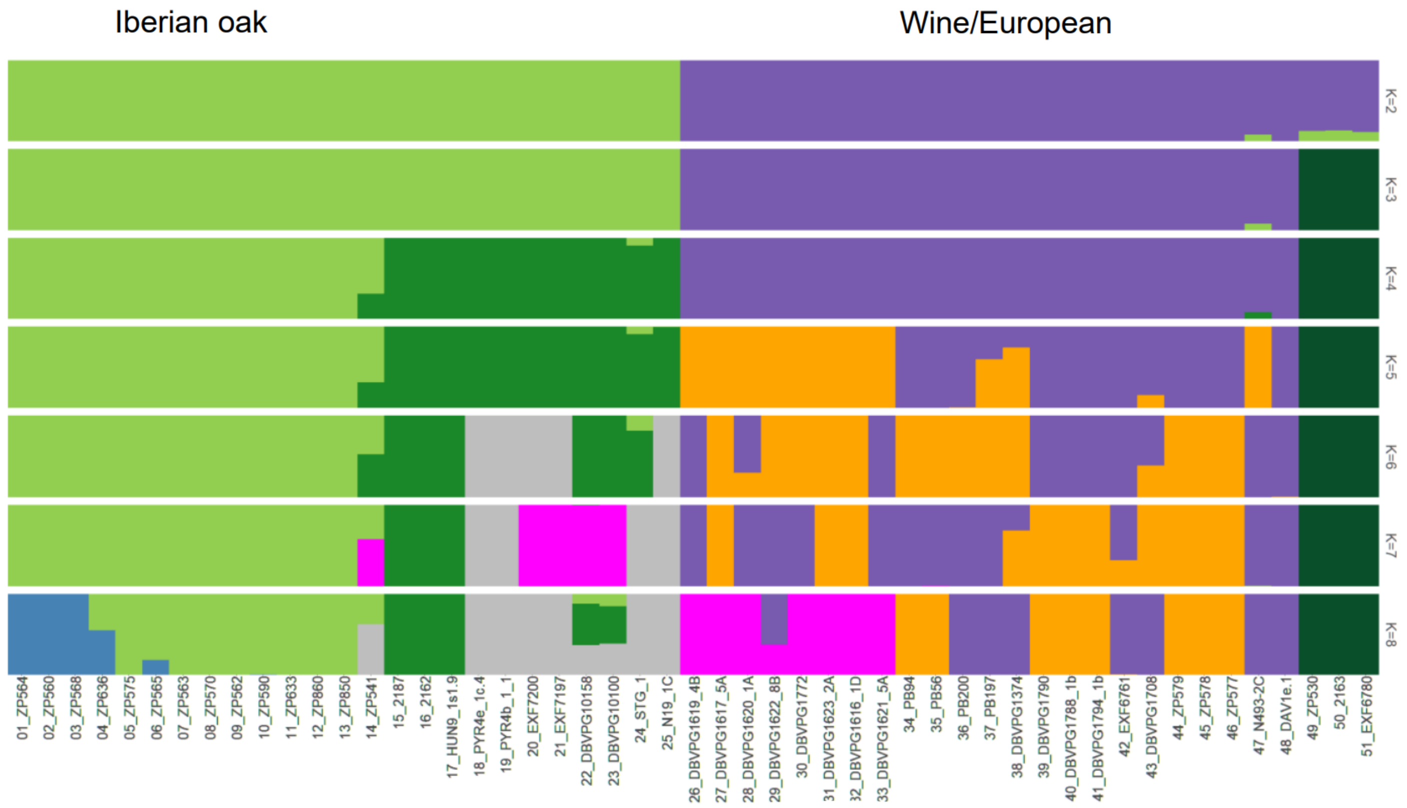

Supplemental Figure 7 The population structure and admixture of wild *Saccharomyces cerevisiae* from Europe. Populations were estimated using ADMIXTURE from varying cluster (K) 2-8 with five replicate runs for each K. We selected the run that had the highest loglikelihood value from each K and strains are ordered by their position in the neighbor-joining tree.

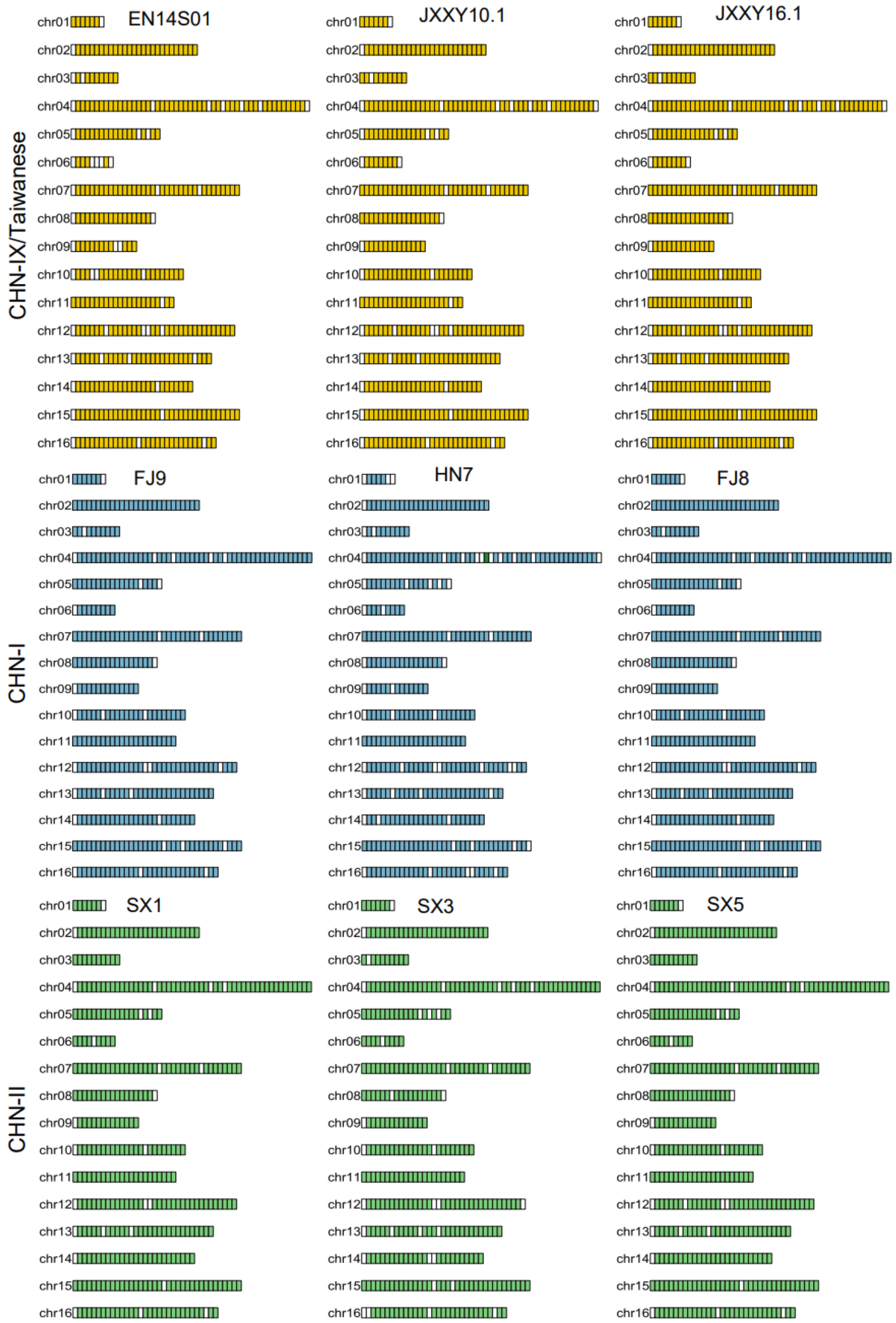
CHN-IX/Taiwanese
EN14S01
JXXY10.1
JXXY16.1
CHN-I
FJ9
HN7
FJ8
CHN-II
SX1
SX3
SX5
chr01
chr02
chr03
chr04
chr05
chr06
chr07
chr08
chr09
chr10
chr11
chr12
chr13
chr14
chr15
chr16

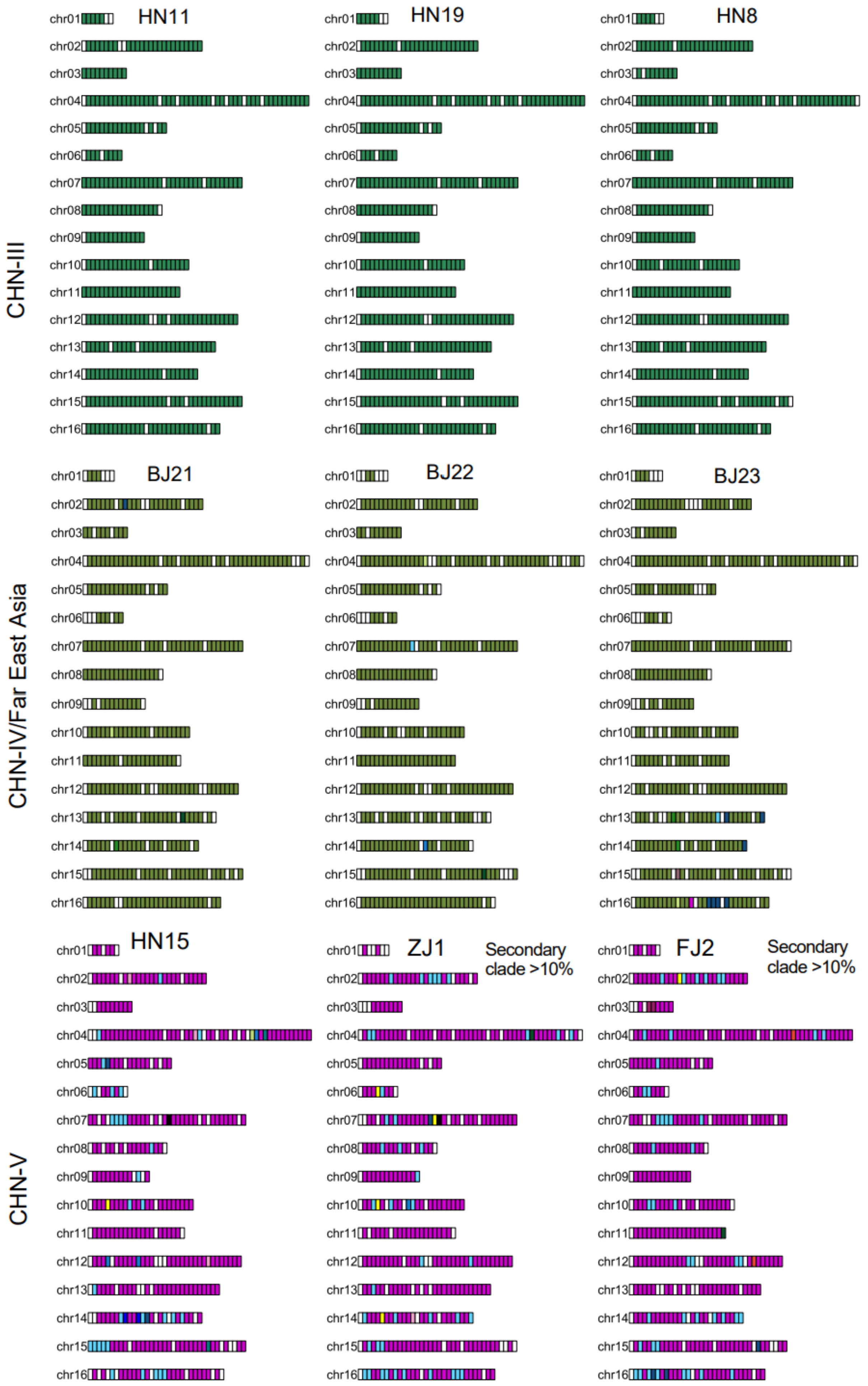
CHN-III
HN11
HN19
HN8
CHN-IV/Far East Asia
BJ21
BJ22
BJ23
CHN-V
HN15
ZJ1
Secondary clade >10%
FJ2
Secondary clade >10%
chr01
chr02
chr03
chr04
chr05
chr06
chr07
chr08
chr09
chr10
chr11
chr12
chr13
chr14
chr15
chr16

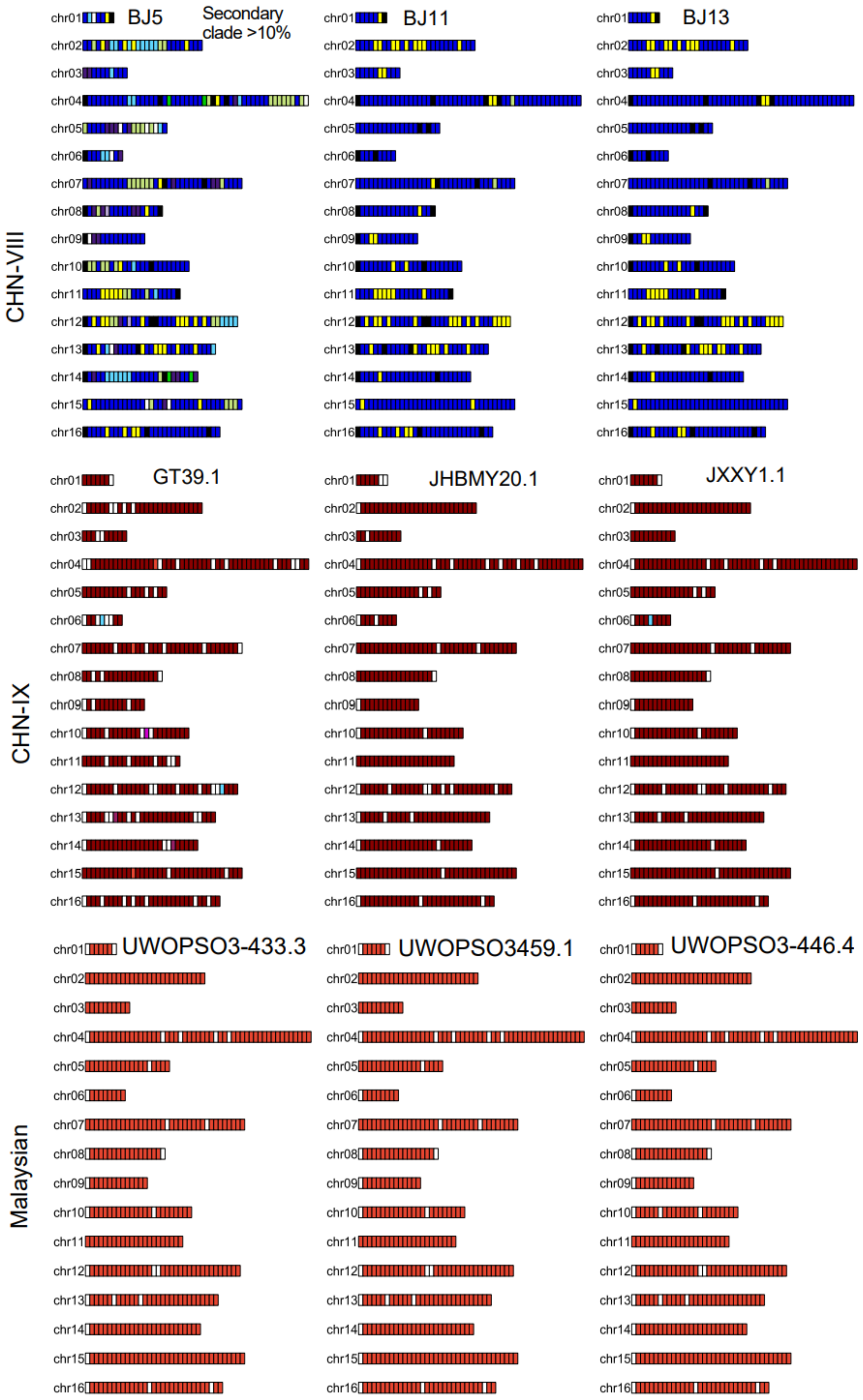
BJ5
Secondary clade >10%
BJ11
BJ13
CHN-VIII
GT39.1
JHBMY20.1
JXXY1.1
CHN-IX
UWOPSO3-433.3
UWOPSO3459.1
UWOPSO3-446.4
Malaysian
chr01
chr02
chr03
chr04
chr05
chr06
chr07
chr08
chr09
chr10
chr11
chr12
chr13
chr14
chr15
chr16

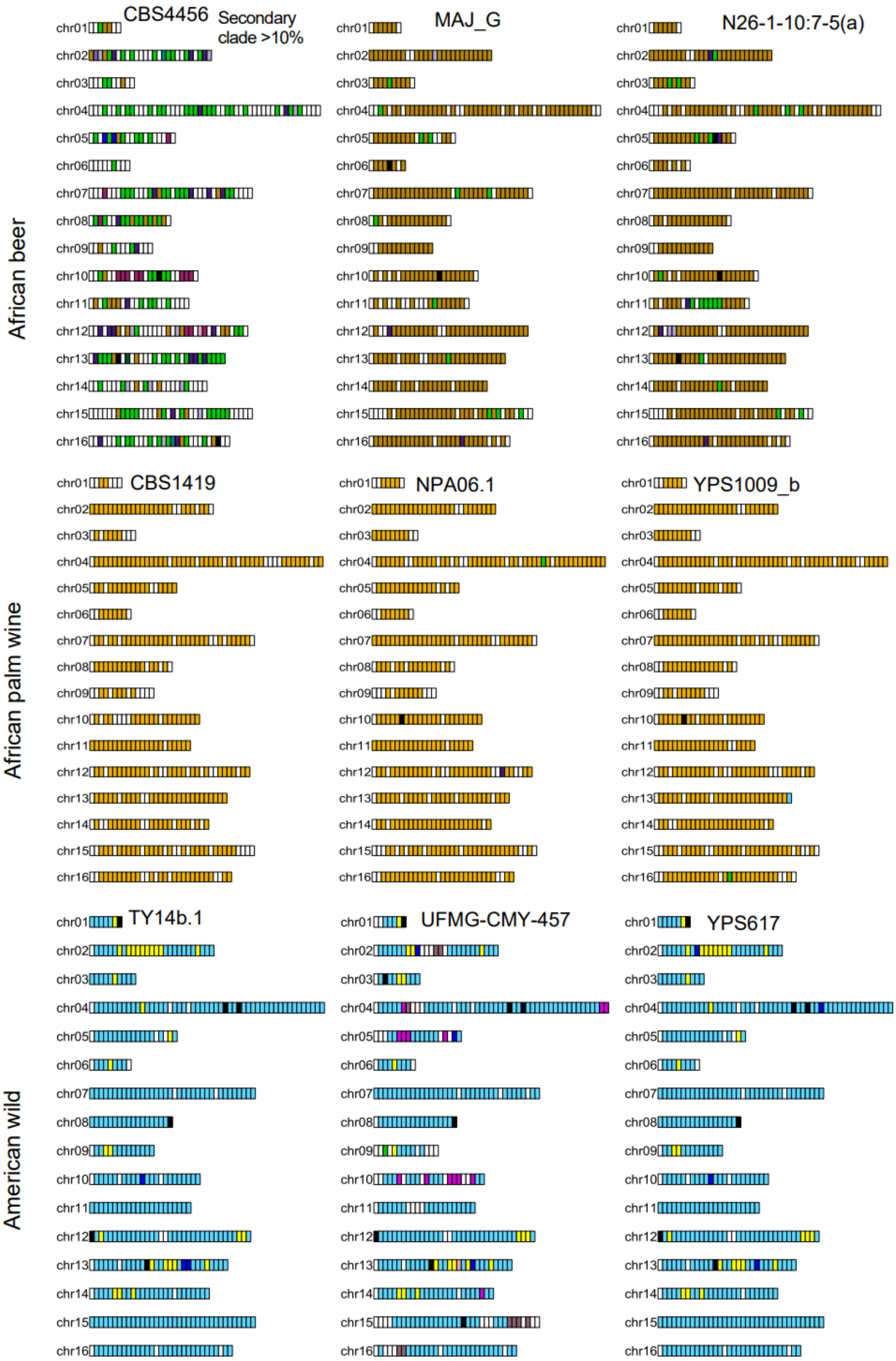
African beer
CBS4456
Secondary clade >10%
MAJ_G
N26-1-10:7-5(a)
African palm wine
CBS1419
NPA06.1
YPS1009_b
American wild
TY14b.1
UFMG-CMY-457
YPS617
chr01
chr02
chr03
chr04
chr05
chr06
chr07
chr08
chr09
chr10
chr11
chr12
chr13
chr14
chr15
chr16

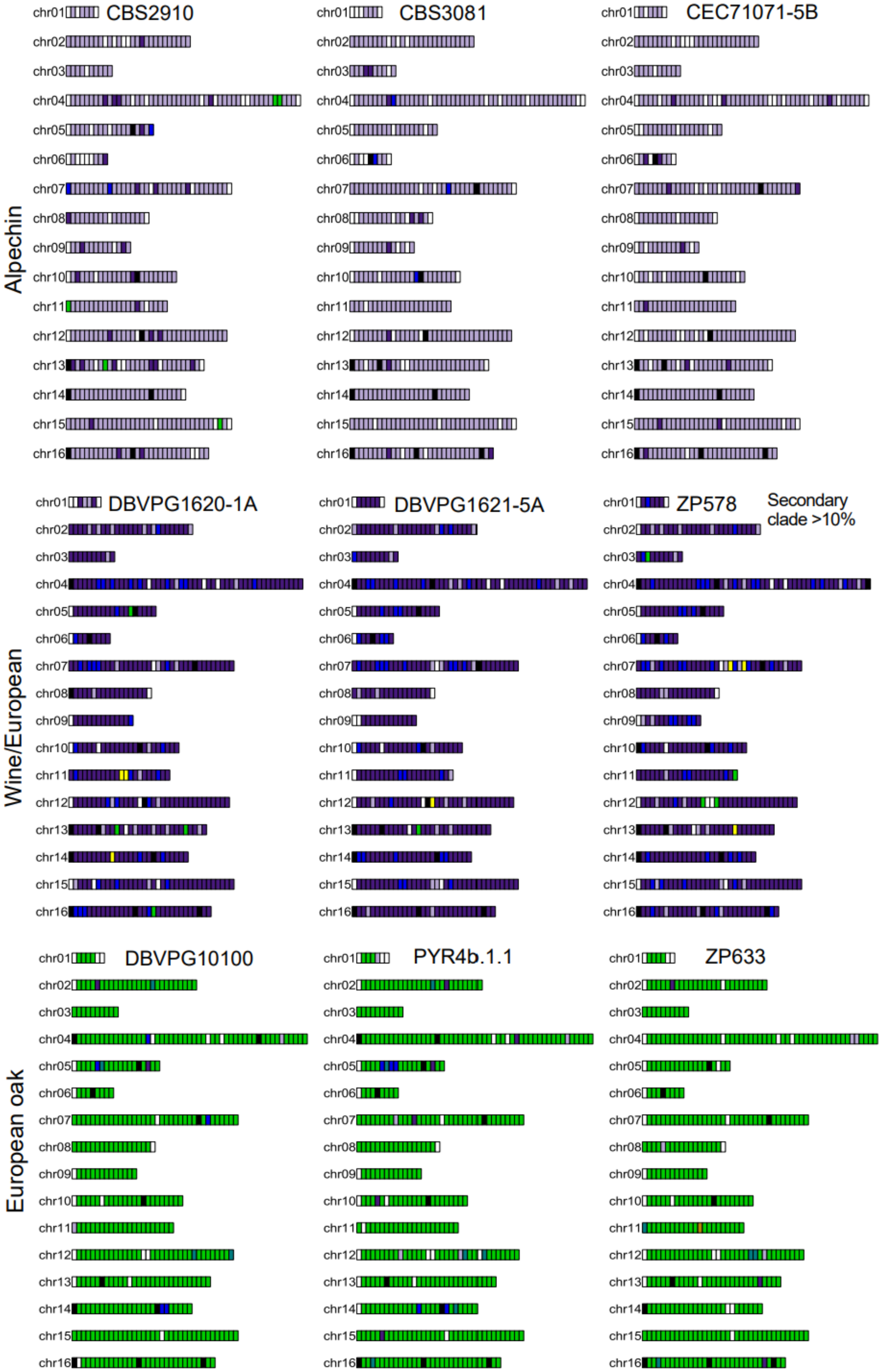
Alpechin
CBS2910
CBS3081
CEC71071-5B
Wine/European
DBVPG1620-1A
DBVPG1621-5A
ZP578
Secondary clade >10%
European oak
DBVPG10100
PYR4b.1.1
ZP633

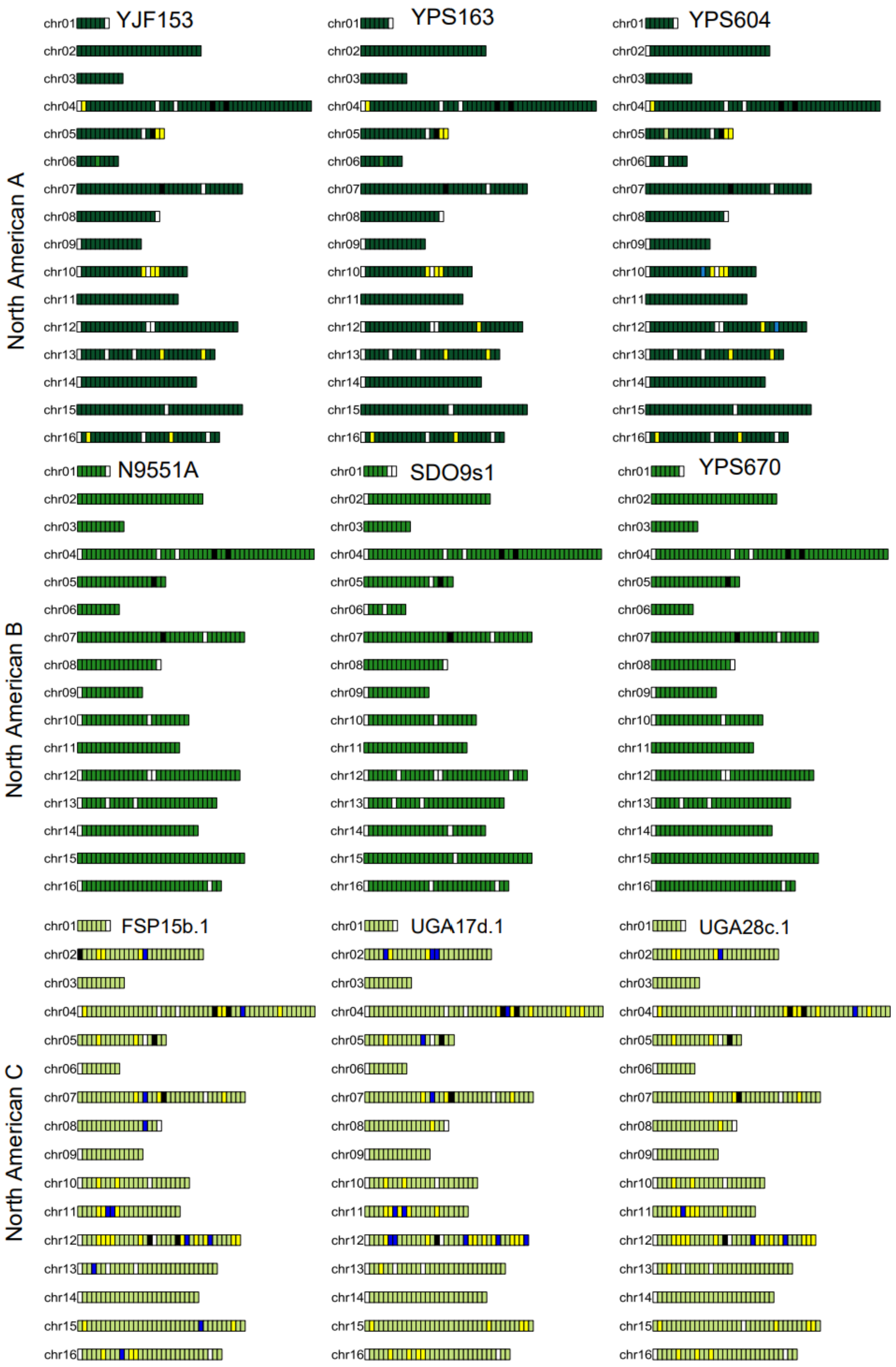
North American A
YJF153
YPS163
YPS604
North American B
N9551A
SDO9s1
YPS670
North American C
FSP15b.1
UGA17d.1
UGA28c.1
chr01
chr02
chr03
chr04
chr05
chr06
chr07
chr08
chr09
chr10
chr11
chr12
chr13
chr14
chr15
chr16

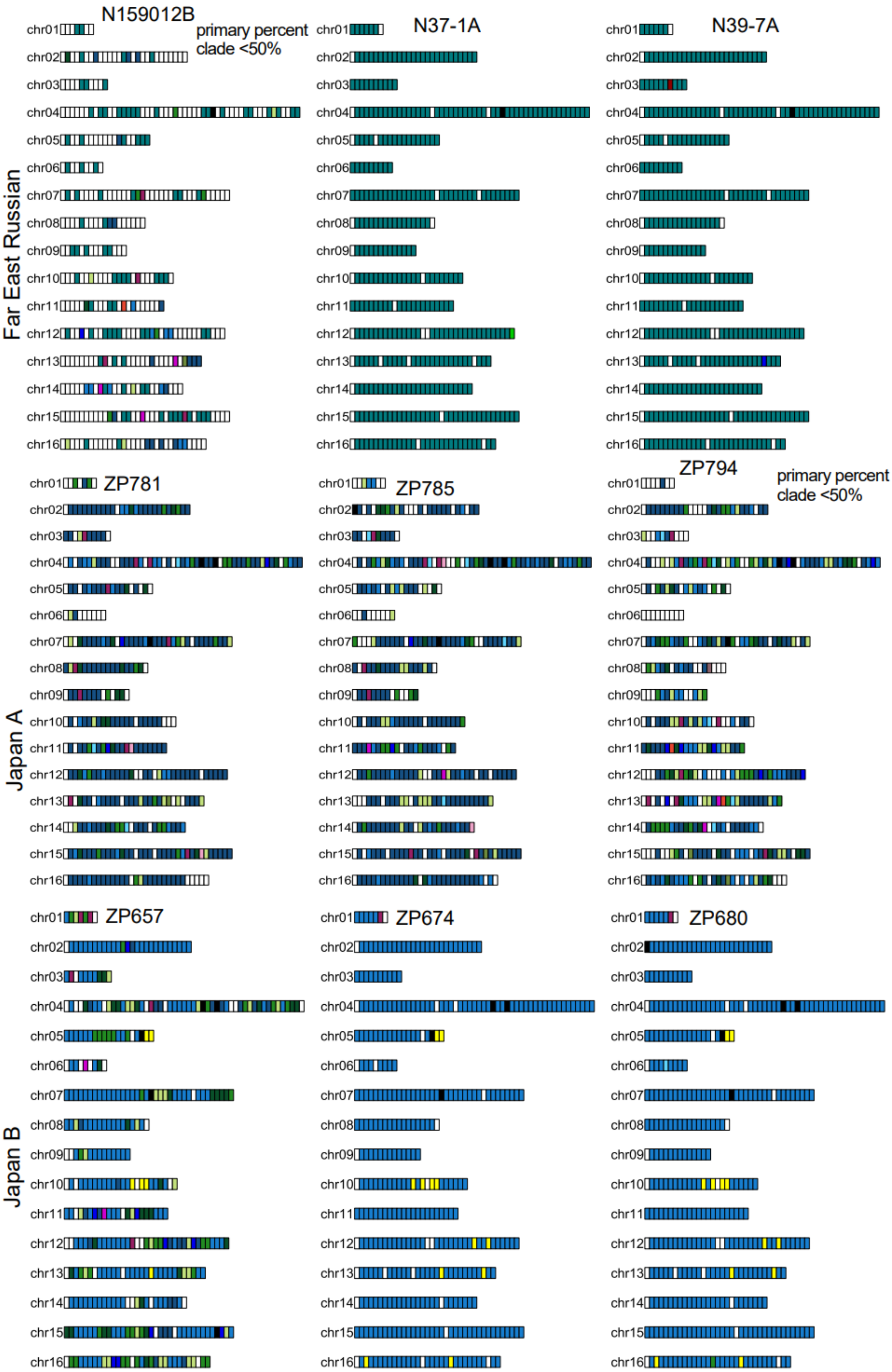
Far East Russian
N159012B
primary percent clade <50%
N37-1A
N39-7A
Japan A
ZP781
ZP785
ZP794
primary percent clade <50%
Japan B
ZP657
ZP674
ZP680

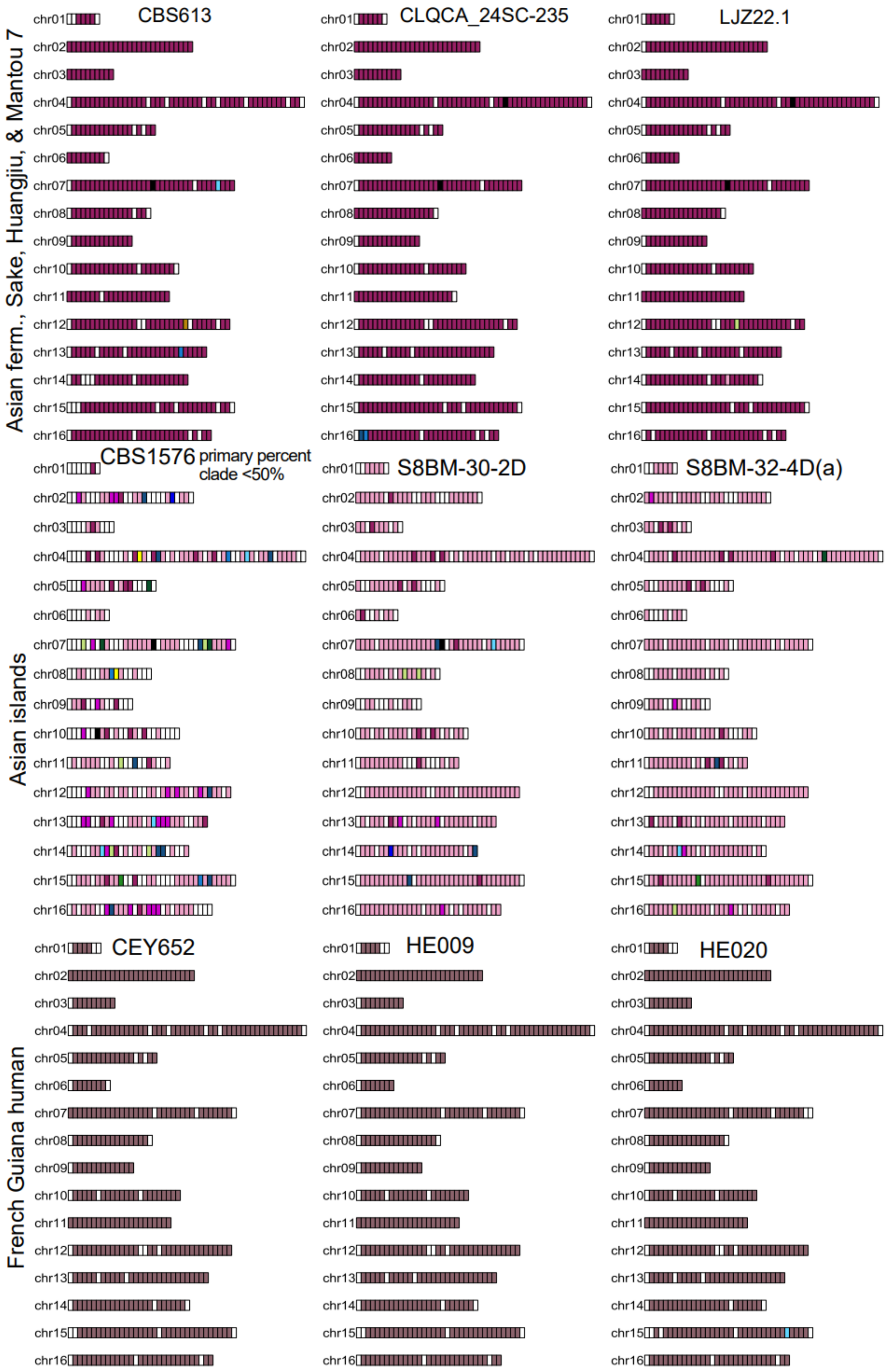
Asian ferm., Sake, Huangjiu, & Mantou 7
CBS613
CLQCA_24SC-235
LJZ22.1
Asian islands
CBS1576
primary percent clade <50%
S8BM-30-2D
S8BM-32-4D(a)
French Guiana human
CEY652
HE009
HE020
chr01
chr02
chr03
chr04
chr05
chr06
chr07
chr08
chr09
chr10
chr11
chr12
chr13
chr14
chr15
chr16

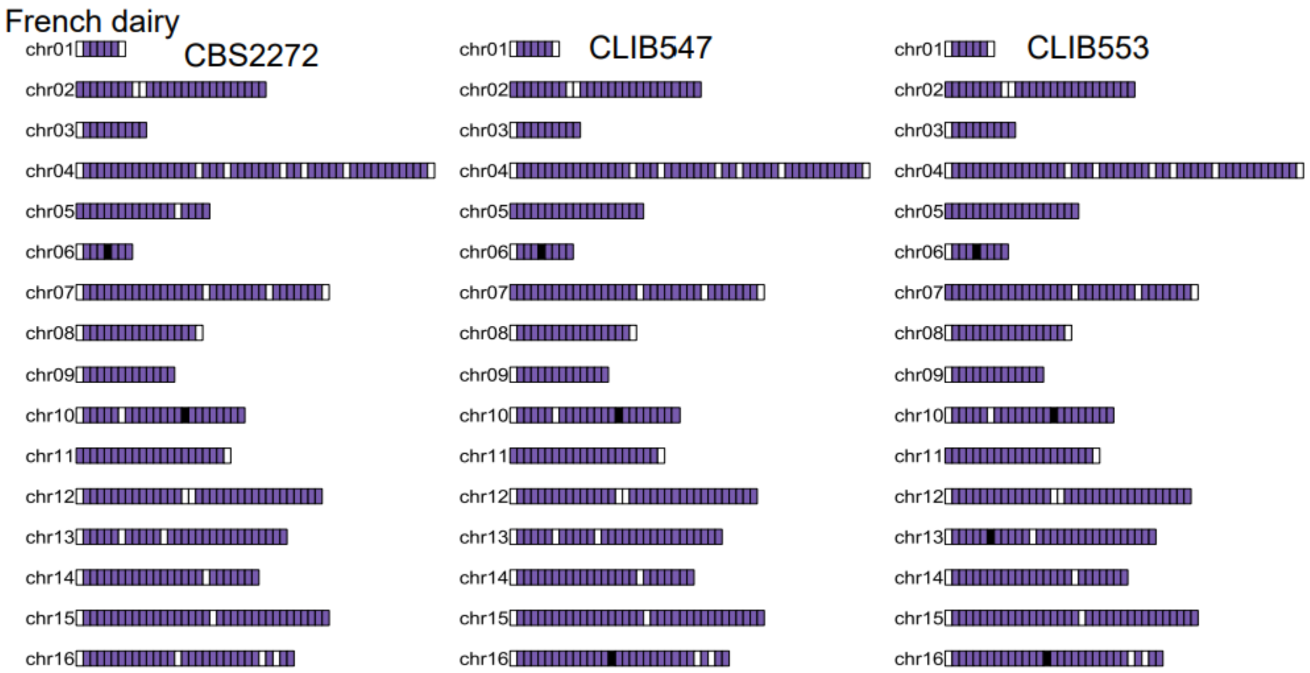

Supplemental Figure 8 Chromosome painting for 77 strains used as a backbone phylogeny (Table S3). Genomic regions were "painted" based on the clade assignment of the most similar strain in 30 kb non-overlapping windows. Diverged regions were not colored (white) and were defined as regions that had a maximum proportion of sites that differed by 0.003 from all other strains in the backbone phylogeny. Black colored regions indicate low coverage, and yellow windows show equal similarity to multiple lineages.

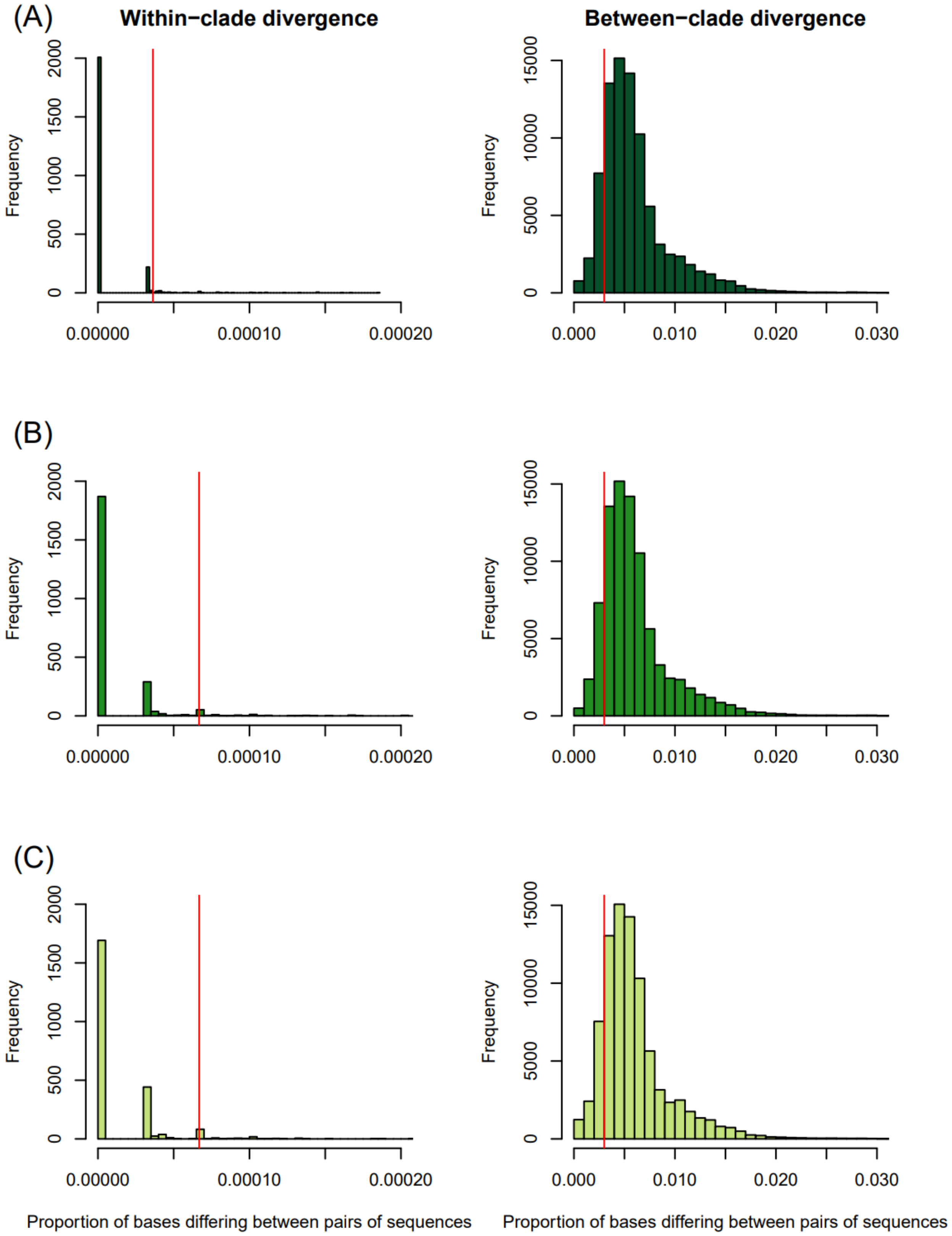

Supplemental Figure 9 Histograms of within-clade and between-clade comparisons for North American A-C (A-C). Within-clade divergences are below the 95th quantile (vertical red line), while between-clade divergences are above 0.003 (vertical red line).

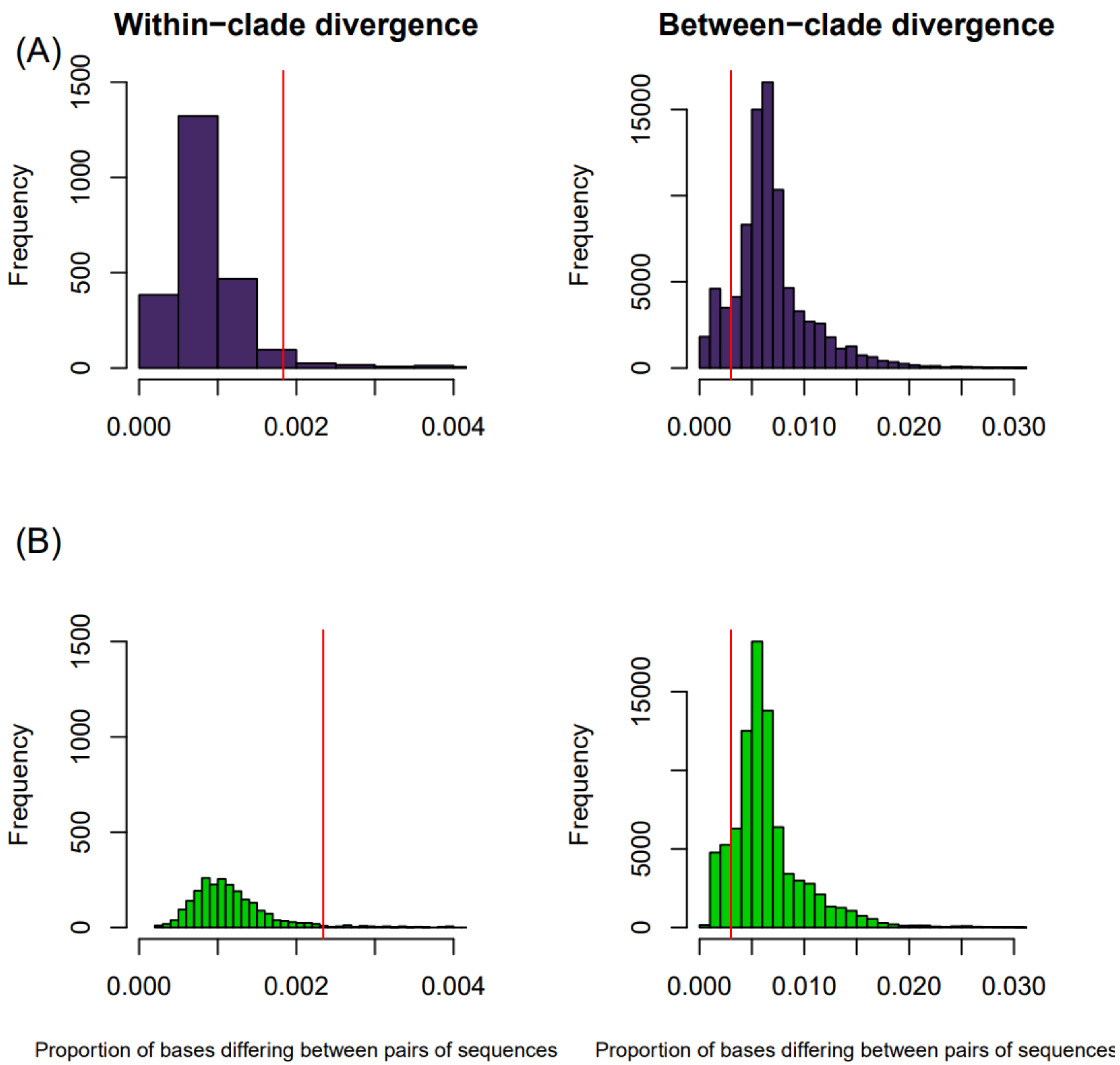

Supplemental Figure 10 Histograms of within-clade and between-clade comparisons for Wine/European (A) and European oak (B). Within-clade divergences are below the 95th quantile (vertical red line), while most between-clade divergences are above 0.003 (vertical red line).

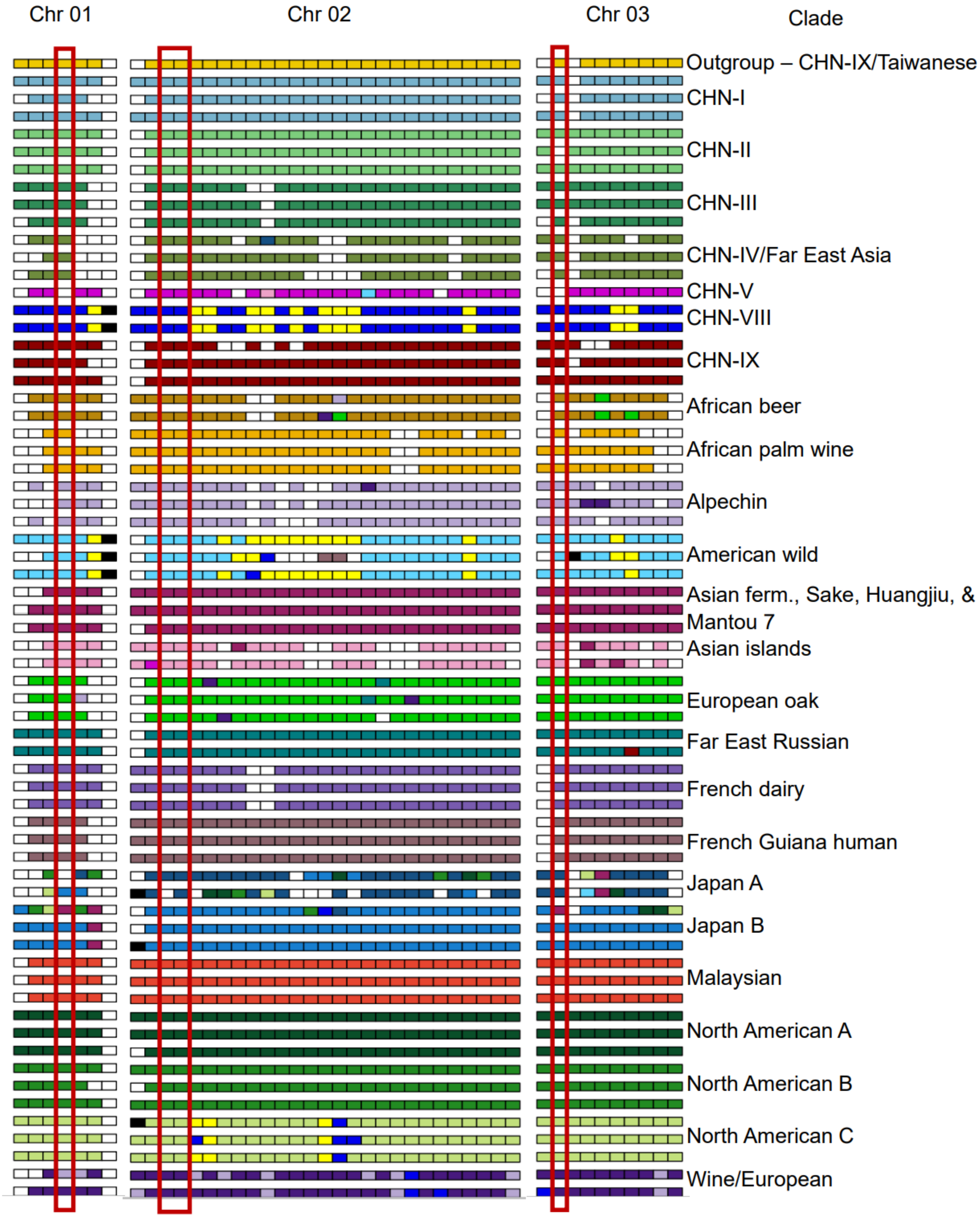
Chr 01
Chr 02
Chr 03
Clade
Outgroup – CHN-IX/Taiwanese
CHN-I
CHN-II
CHN-III
CHN-IV/Far East Asia
CHN-V
CHN-VIII
CHN-IX
African beer
African palm wine
Alpechin
American wild
Asian ferm., Sake, Huangjiu, &
Mantou 7
Asian islands
European oak
Far East Russian
French dairy
French Guiana human
Japan A
Japan B
Malaysian
North American A
North American B
North American C
Wine/European

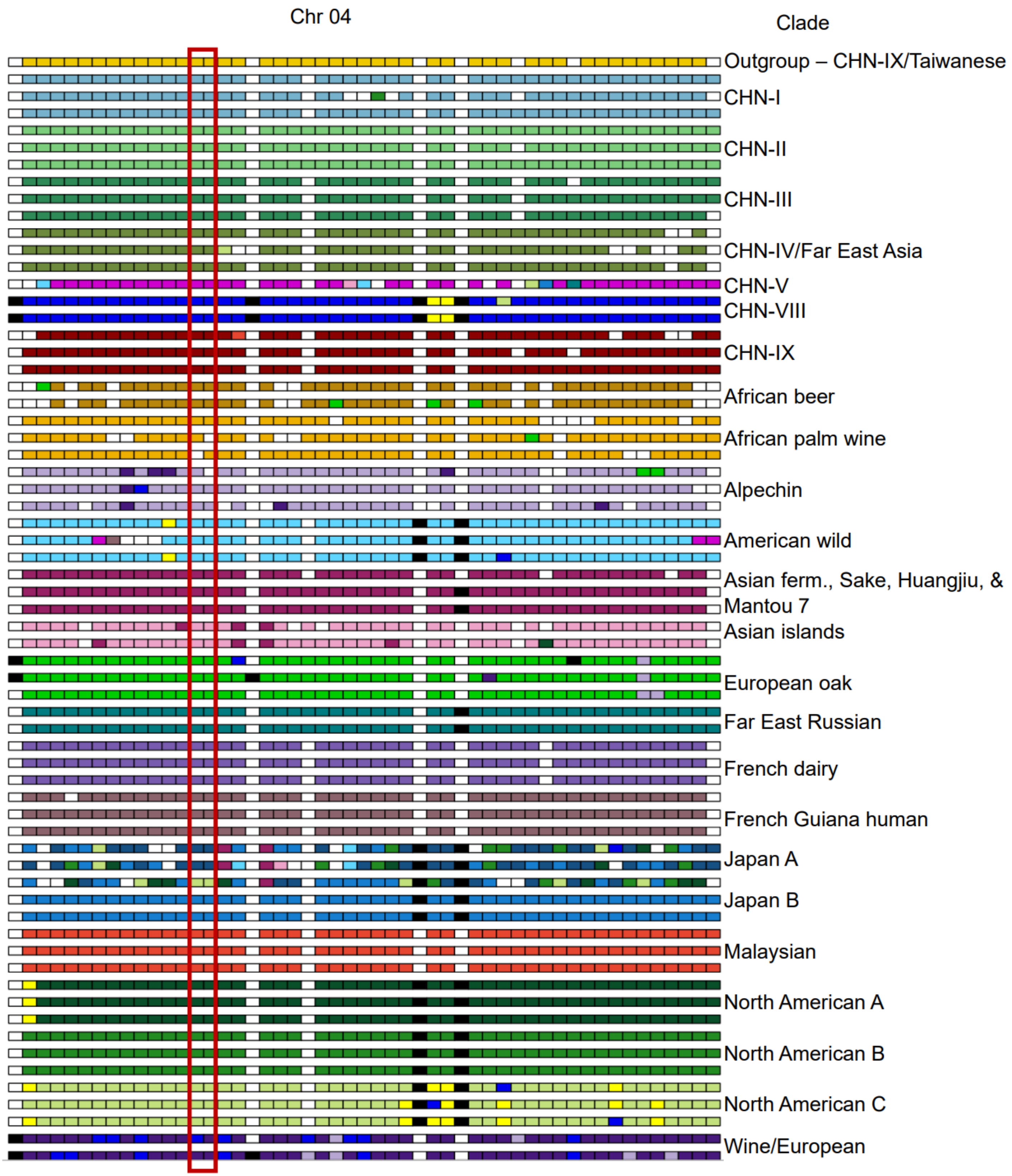
Chr 04
Clade
Outgroup – CHN-IX/Taiwanese
CHN-I
CHN-II
CHN-III
CHN-IV/Far East Asia
CHN-V
CHN-VIII
CHN-IX
African beer
African palm wine
Alpechin
American wild
Asian ferm., Sake, Huangjiu, &
Mantou 7
Asian islands
European oak
Far East Russian
French dairy
French Guiana human
Japan A
Japan B
Malaysian
North American A
North American B
North American C
Wine/European

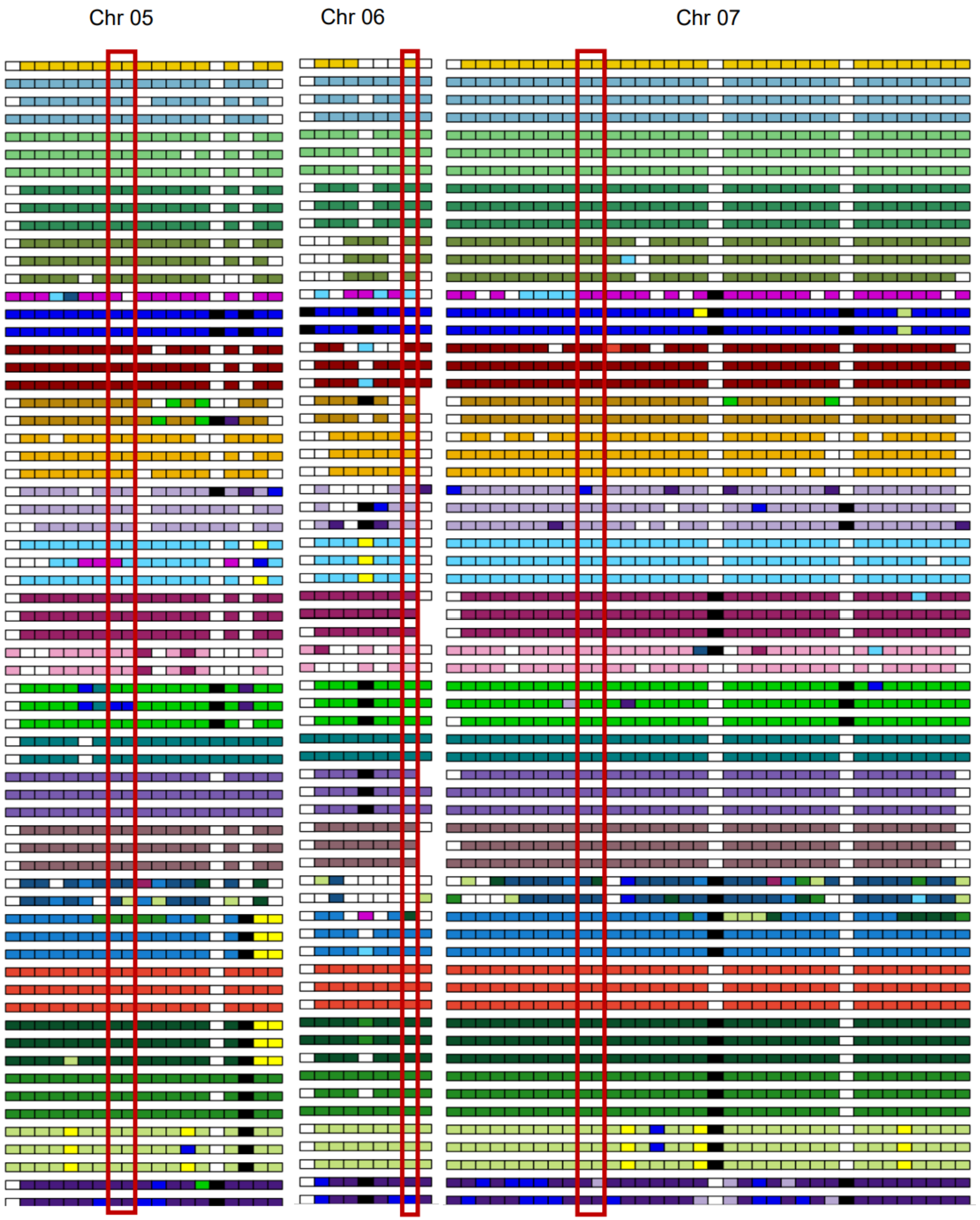
Chr 05
Chr 06
Chr 07

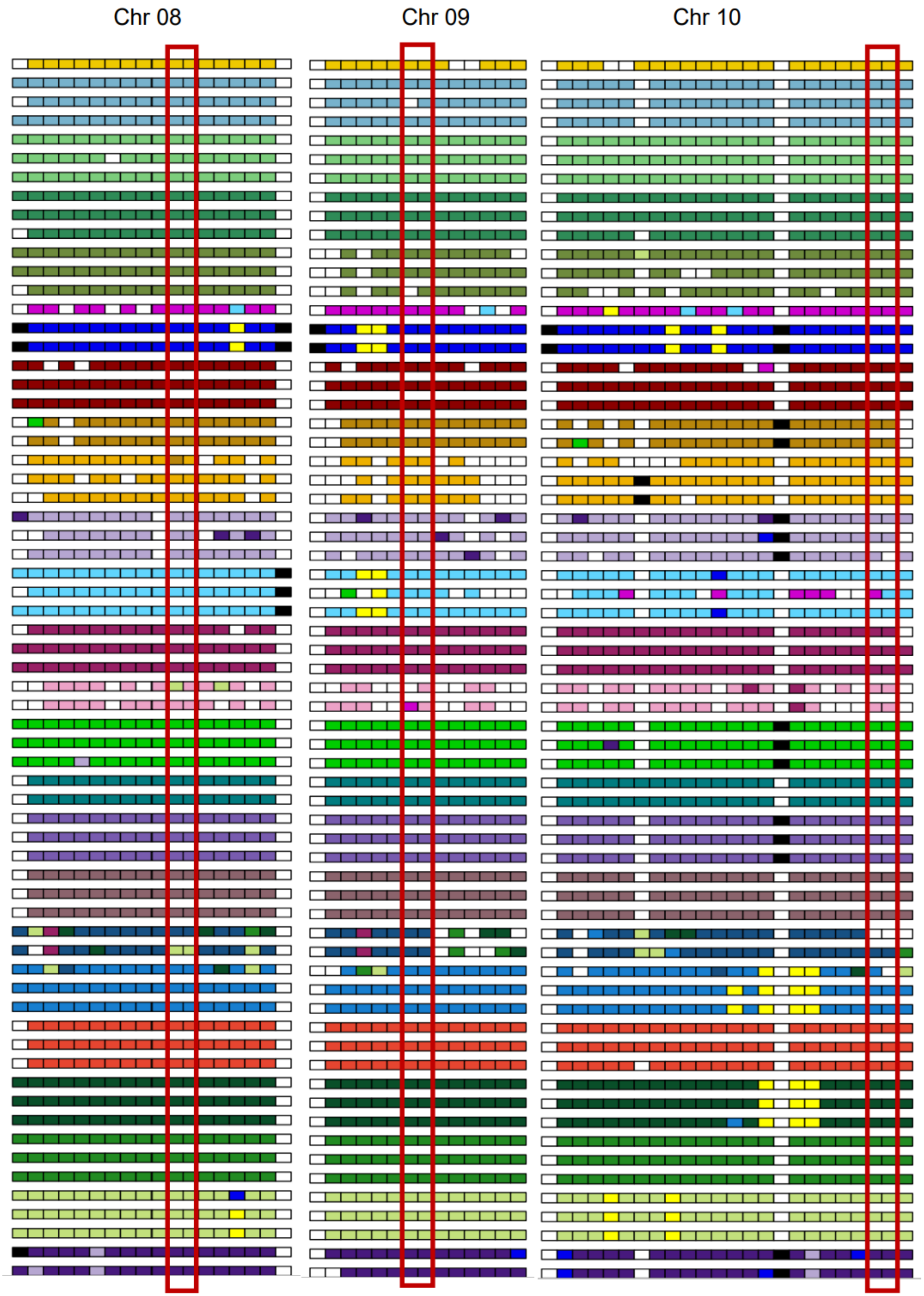
Chr 08
Chr 09
Chr 10

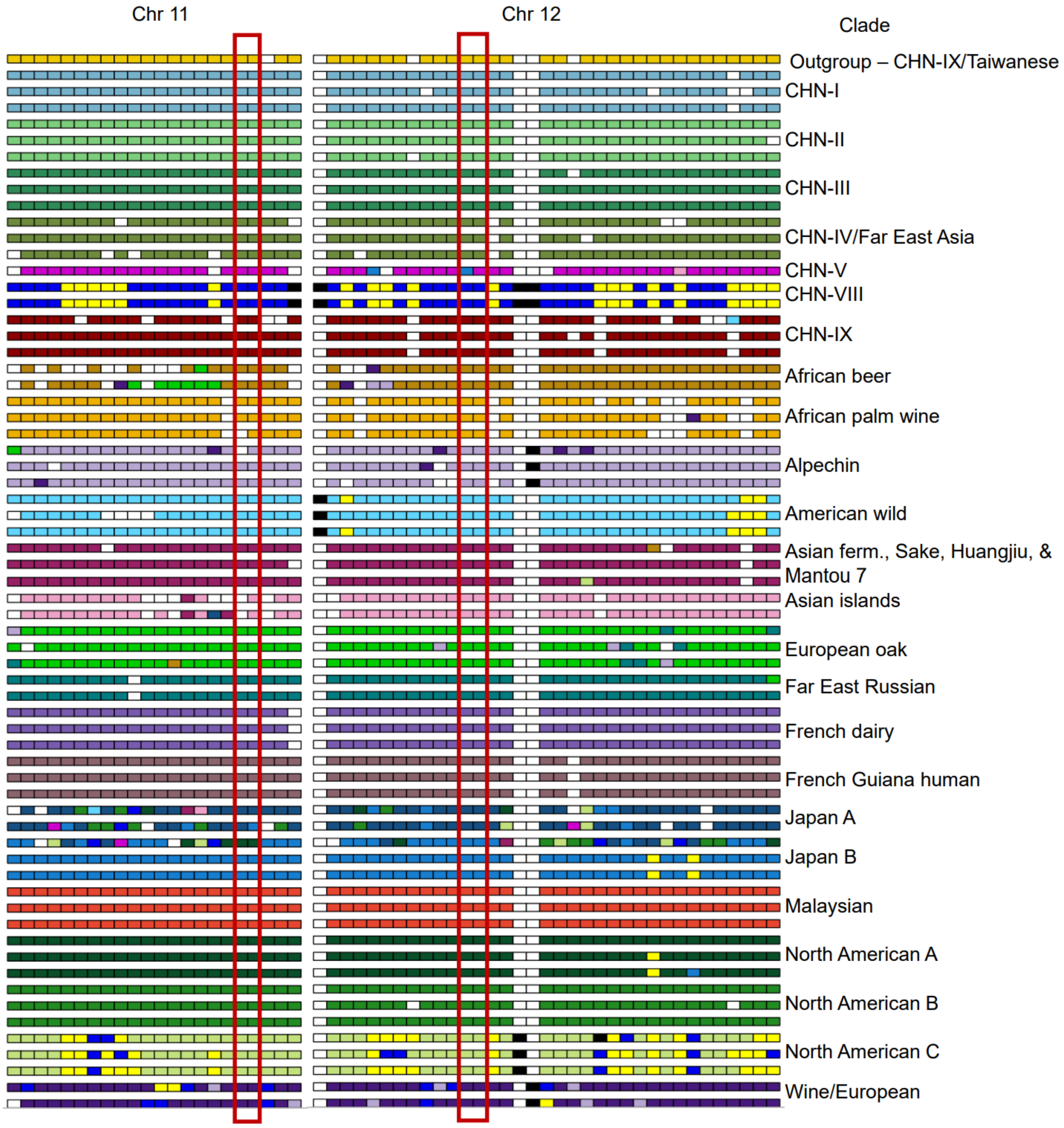
Chr 11
Chr 12
Clade
Outgroup – CHN-IX/Taiwanese
CHN-I
CHN-II
CHN-III
CHN-IV/Far East Asia
CHN-V
CHN-VIII
CHN-IX
African beer
African palm wine
Alpechin
American wild
Asian ferm., Sake, Huangjiu, & Mantou 7
Asian islands
European oak
Far East Russian
French dairy
French Guiana human
Japan A
Japan B
Malaysian
North American A
North American B
North American C
Wine/European

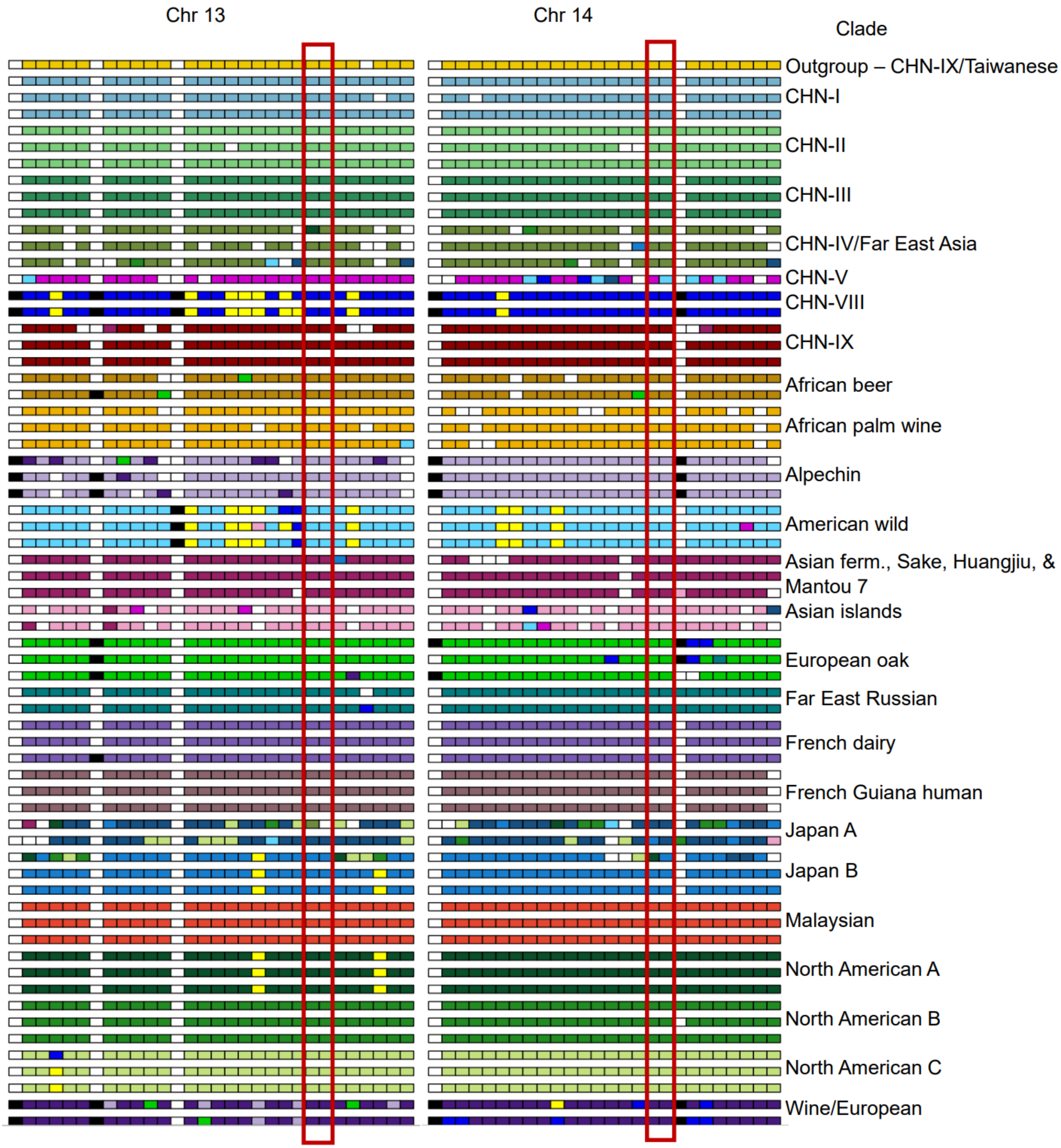
Chr 13
Chr 14
Clade
Outgroup – CHN-IX/Taiwanese
CHN-I
CHN-II
CHN-III
CHN-IV/Far East Asia
CHN-V
CHN-VIII
CHN-IX
African beer
African palm wine
Alpechin
American wild
Asian ferm., Sake, Huangjiu, & Mantou 7
Asian islands
European oak
Far East Russian
French dairy
French Guiana human
Japan A
Japan B
Malaysian
North American A
North American B
North American C
Wine/European

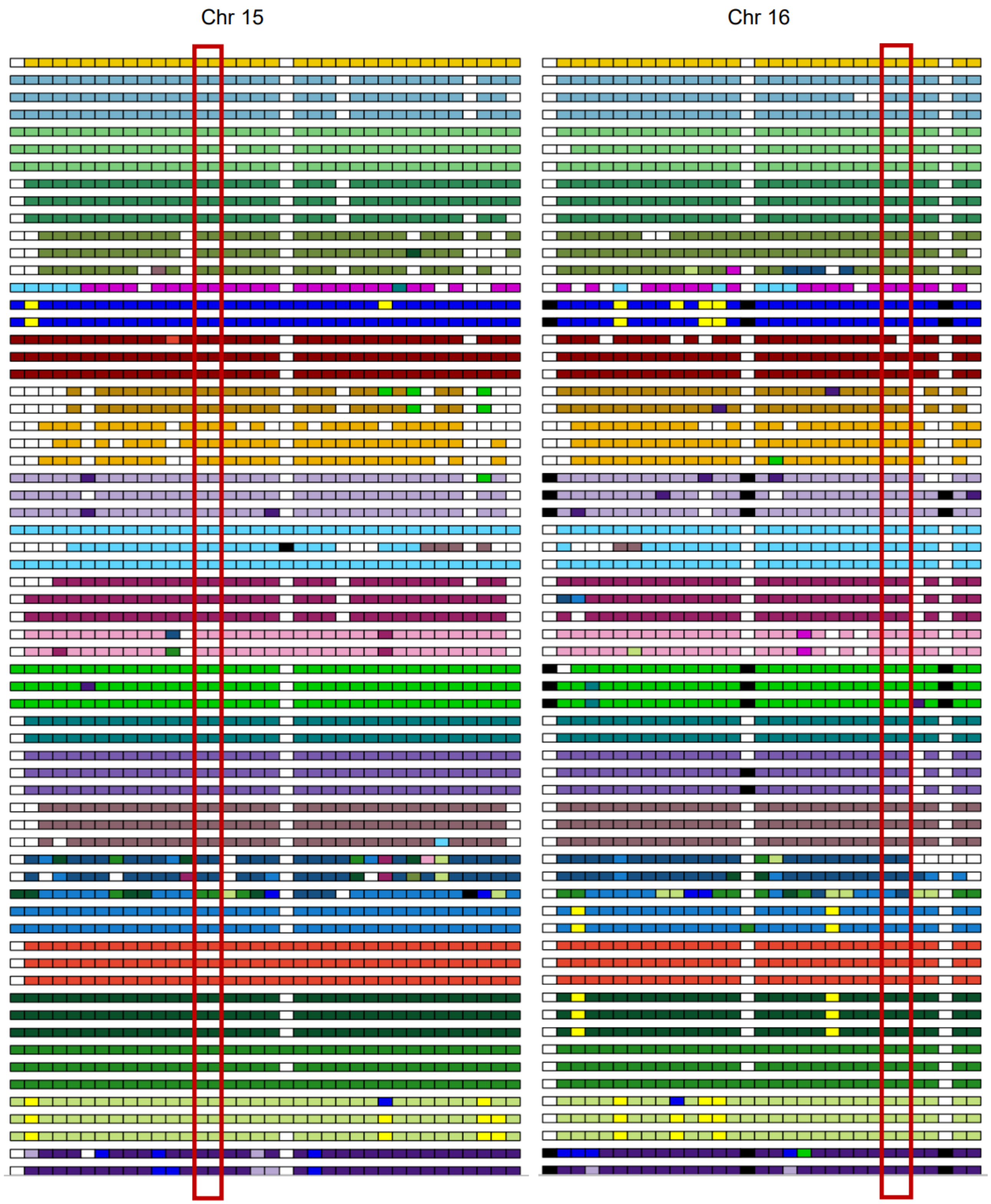

Supplemental Figure 11 in silico chromosome painting results of backbone phylogeny strains grouped by chromosome of strains that made it past quality filters for time divergence analysis (Table S5). Red boxes indicate 30-60 kb genomic regions selected for time divergence analysis when strains are assigned to their primary clade (>50%). Diverged regions were not colored (white) and were defined as regions that had a maximum proportion of sites that differed by 0.003 from all other strains in the backbone phylogeny. Black colored regions indicate low coverage. Genomic regions that show equal genetic similarity to strains from multiple clades are colored yellow.

Chromosome 01

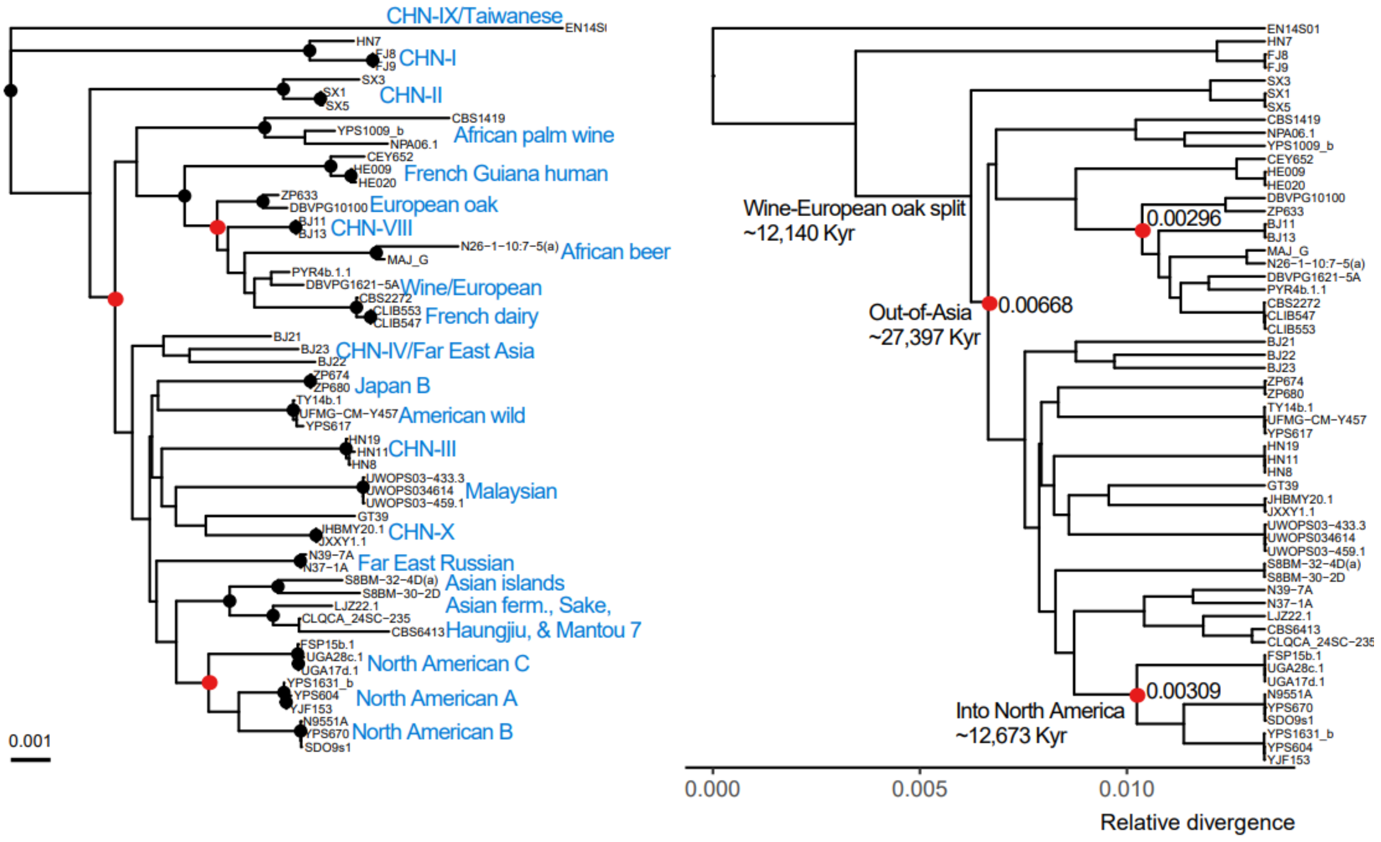

Chromosome 02

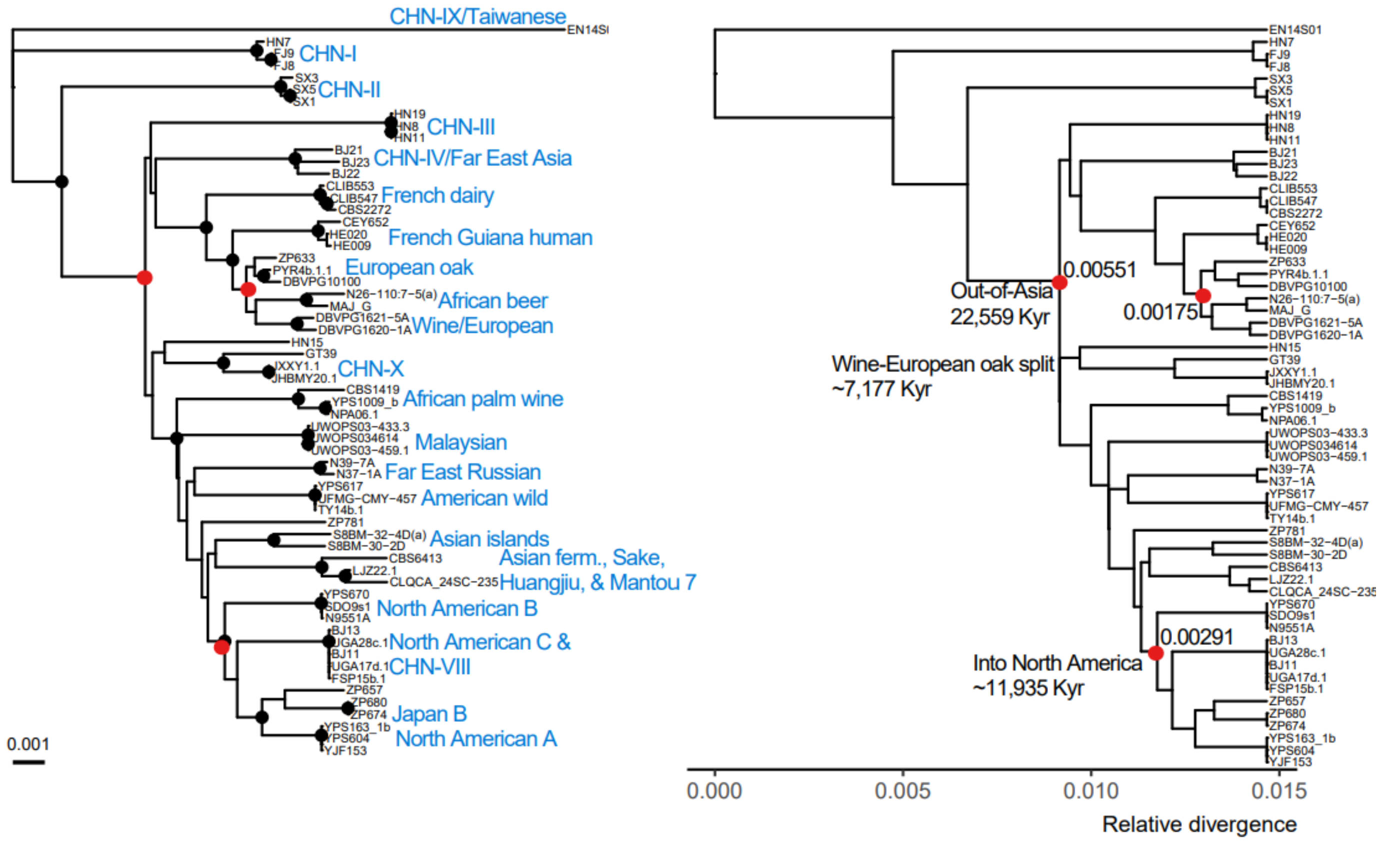

Chromosome 03

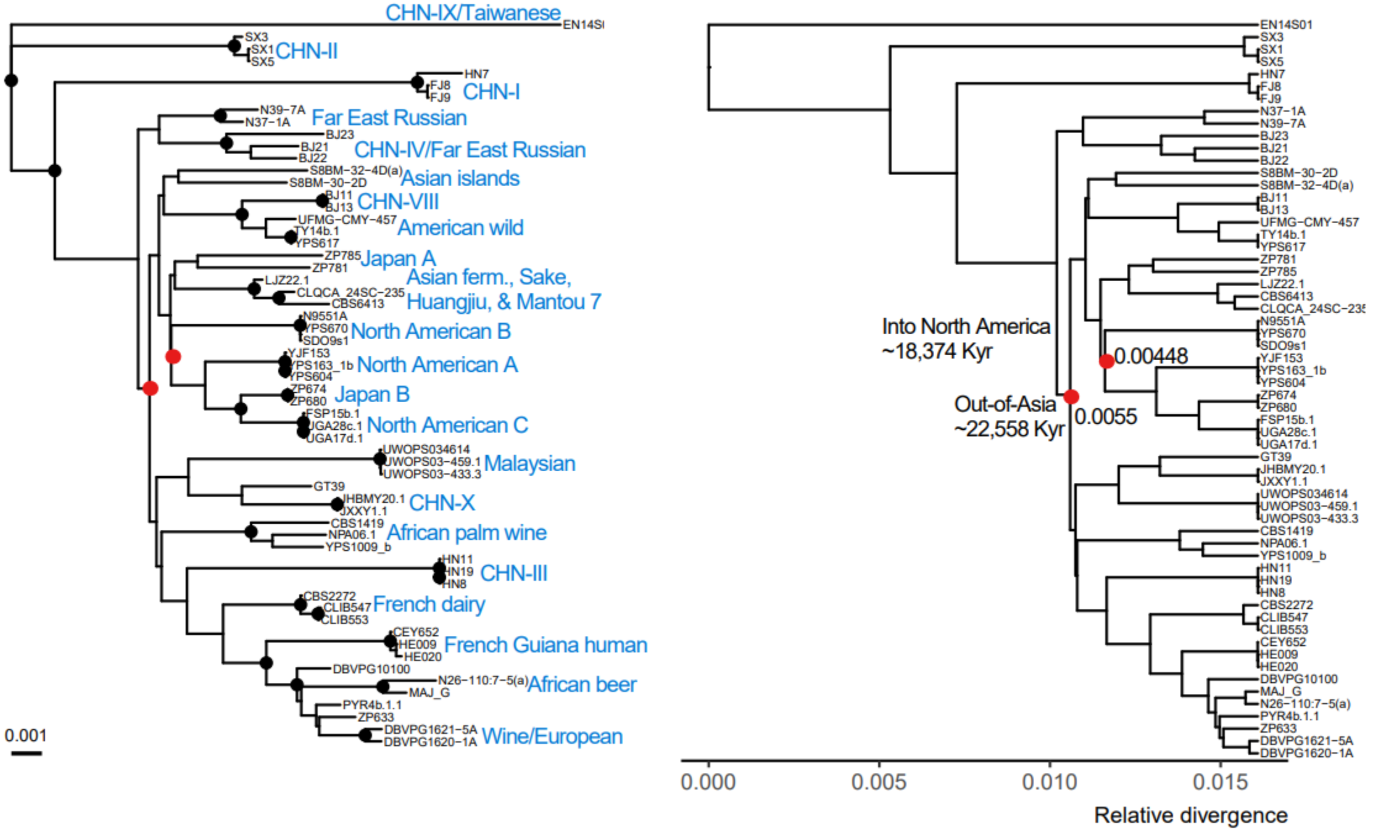

Chromosome 04

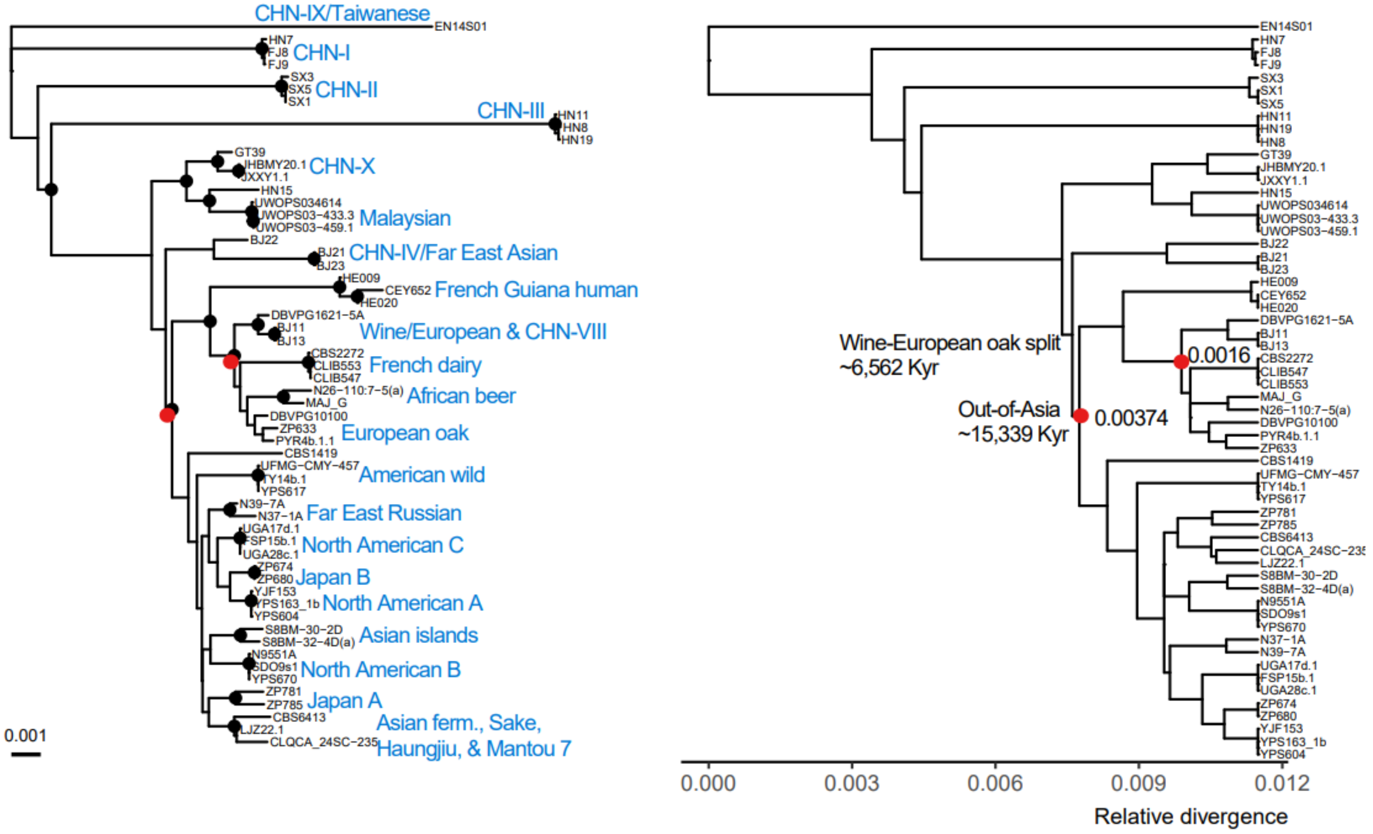

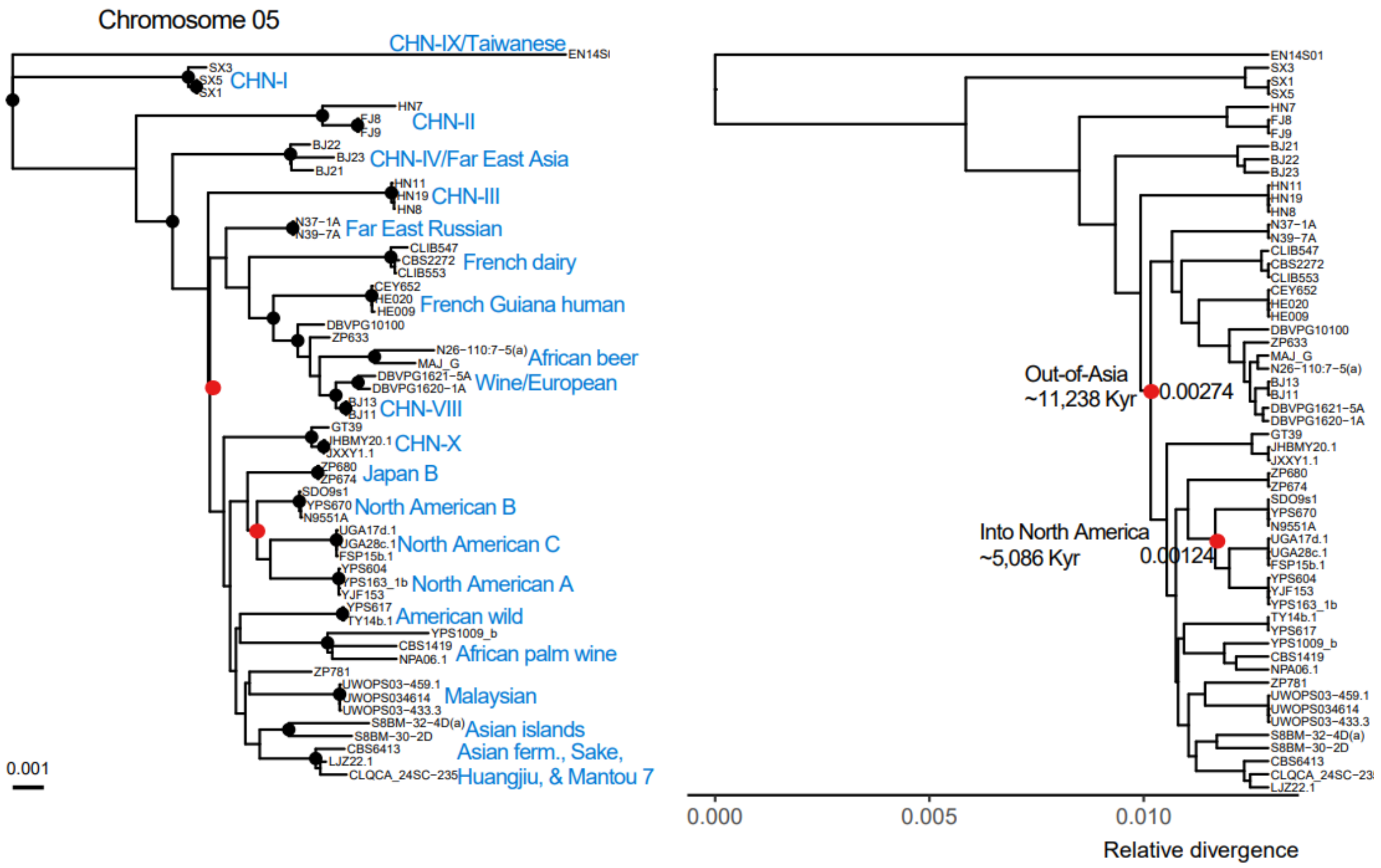
Chromosome 05
CHN-IX/Taiwanese
CHN-I
CHN-II
CHN-IV/Far East Asia
CHN-III
Far East Russian
French dairy
French Guiana human
African beer
Wine/European
CHN-VIII
CHN-X
Japan B
North American B
North American C
North American A
American wild
African palm wine
Malaysian
Asian islands
Asian ferm., Sake,
Huangjiu, & Mantou 7
0.001
Out-of-Asia
~11,238 Kyr
0.00274
Into North America
~5,086 Kyr
0.00124
0.000
0.005
0.010
Relative divergence

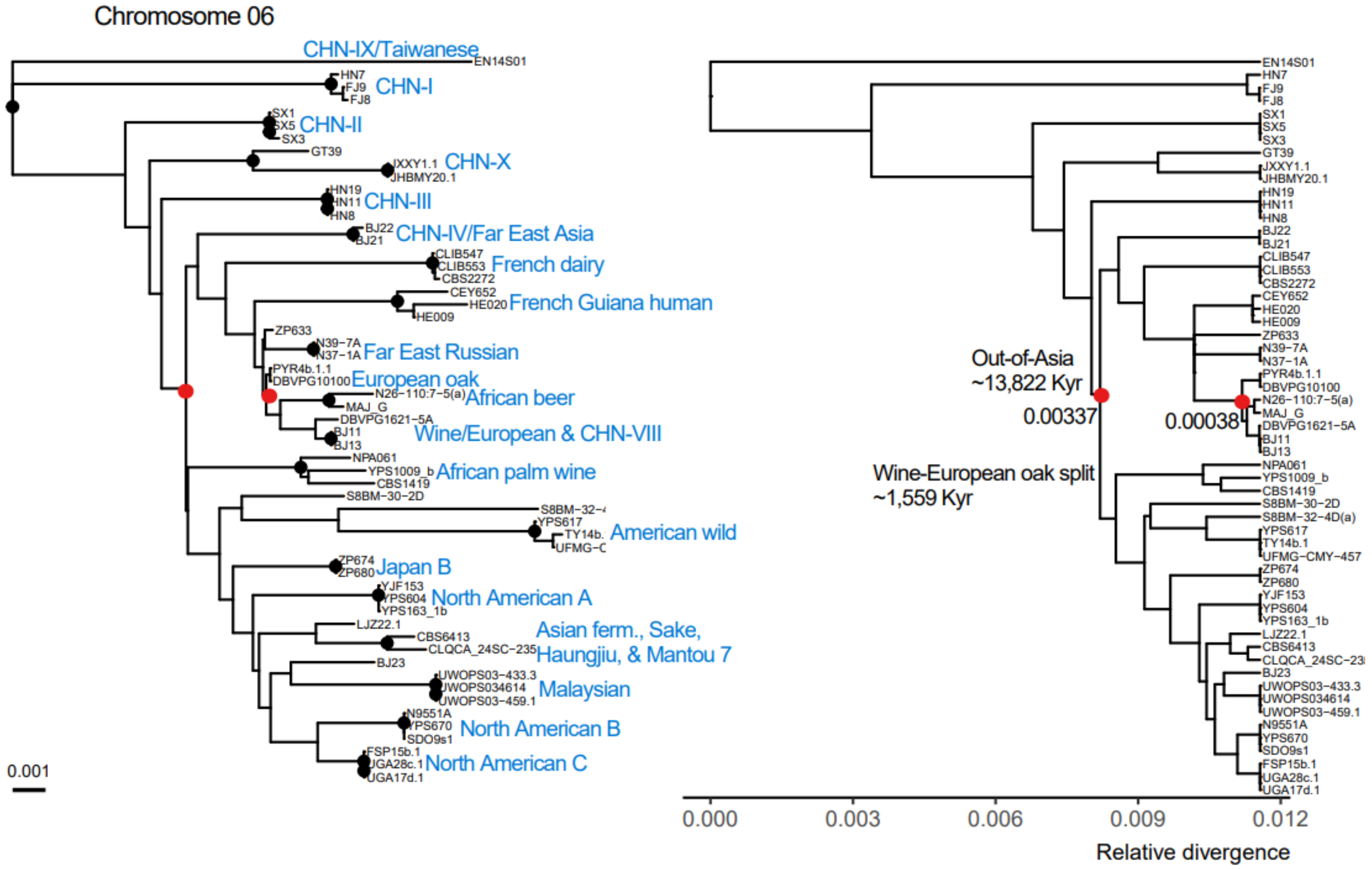
Chromosome 06
CHN-IX/Taiwanese
CHN-I
CHN-II
CHN-X
CHN-III
CHN-IV/Far East Asia
French dairy
French Guiana human
Far East Russian
European oak
African beer
Wine/European & CHN-VIII
African palm wine
American wild
Japan B
North American A
Asian ferm., Sake,
Haungjiu, & Mantou 7
Malaysian
North American B
North American C
0.001
Out-of-Asia
~13,822 Kyr
0.00337
0.00038
Wine-European oak split
~1,559 Kyr
0.000
0.003
0.006
0.009
0.012
Relative divergence

Chromosome 07

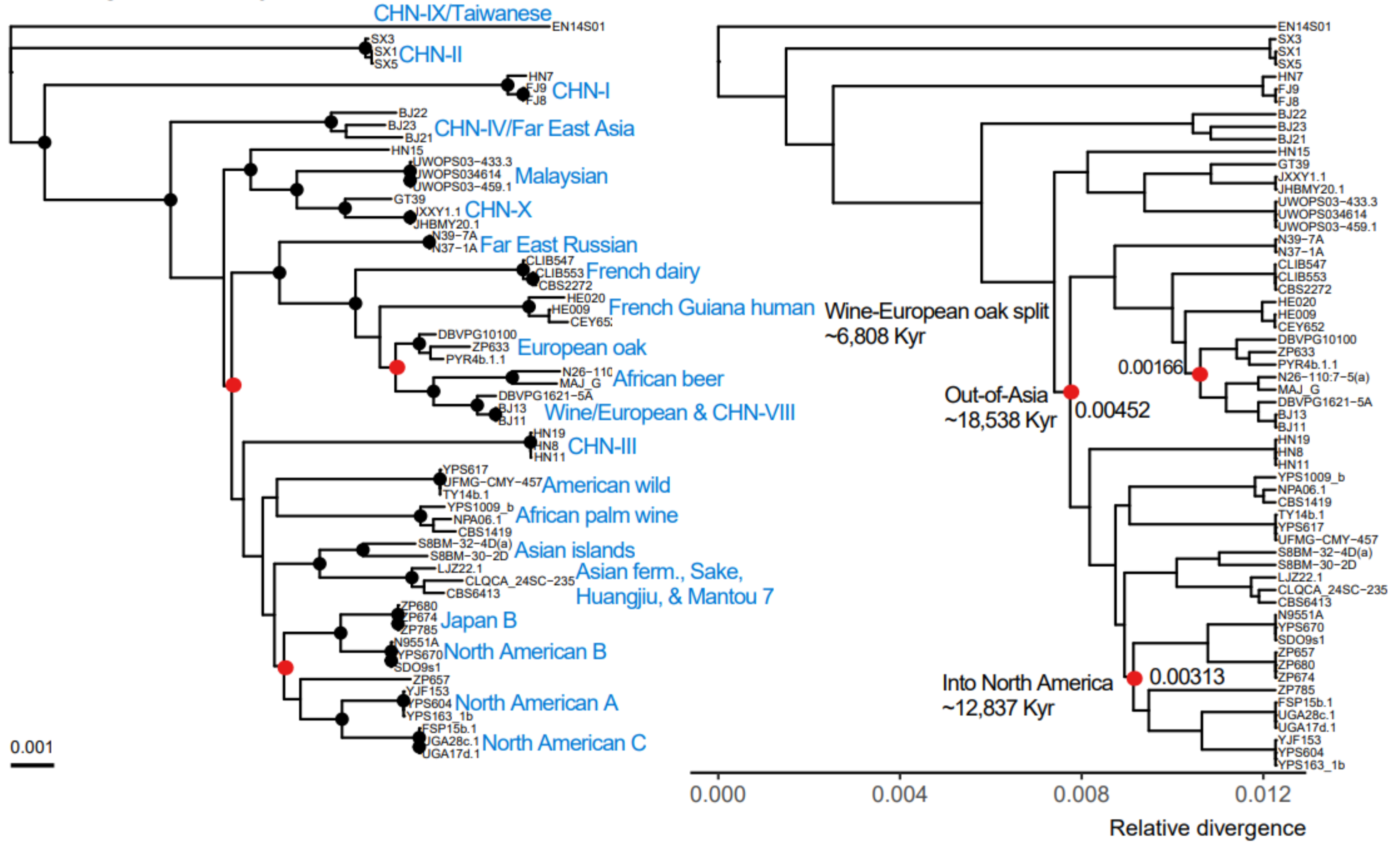

Chromosome 08

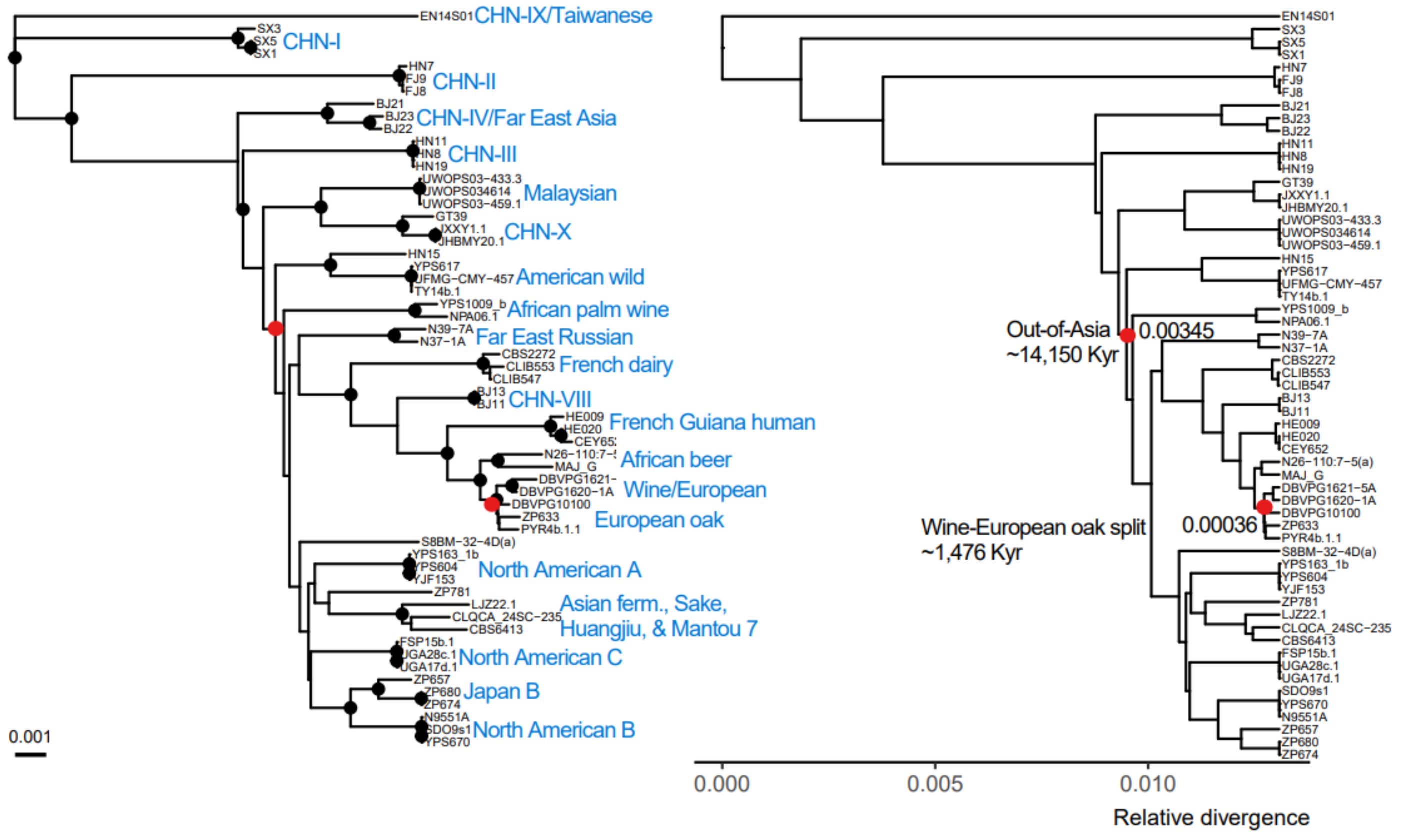

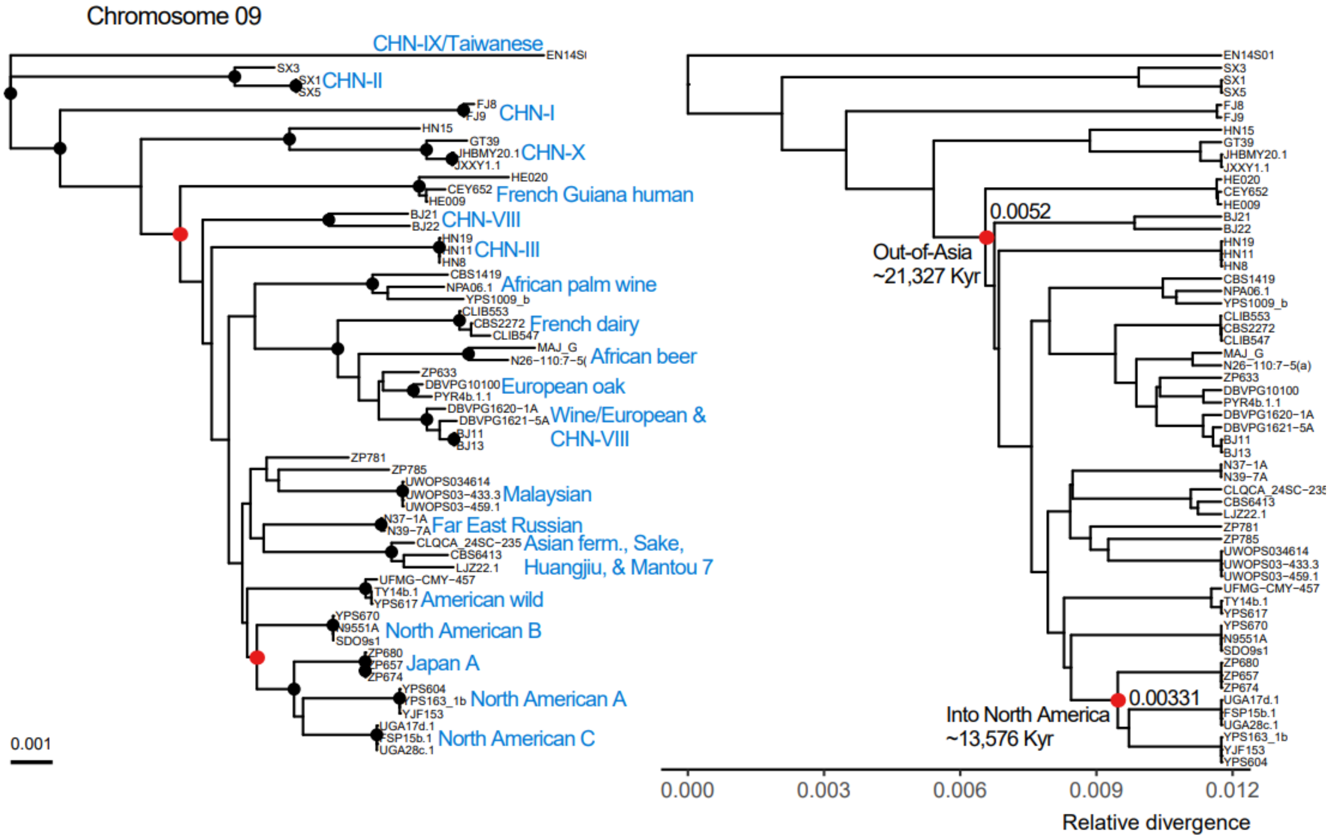
Chromosome 09
CHN-IX/Taiwanese
CHN-II
CHN-I
CHN-X
French Guiana human
CHN-VIII
CHN-III
African palm wine
French dairy
African beer
European oak
Wine/European &
CHN-VIII
Malaysian
Far East Russian
Asian ferm., Sake,
Huangjiu, & Mantou 7
American wild
North American B
Japan A
North American A
North American C
0.001
0.0052
Out-of-Asia
~21,327 Kyr
0.00331
Into North America
~13,576 Kyr
0.000
0.003
0.006
0.009
0.012
Relative divergence

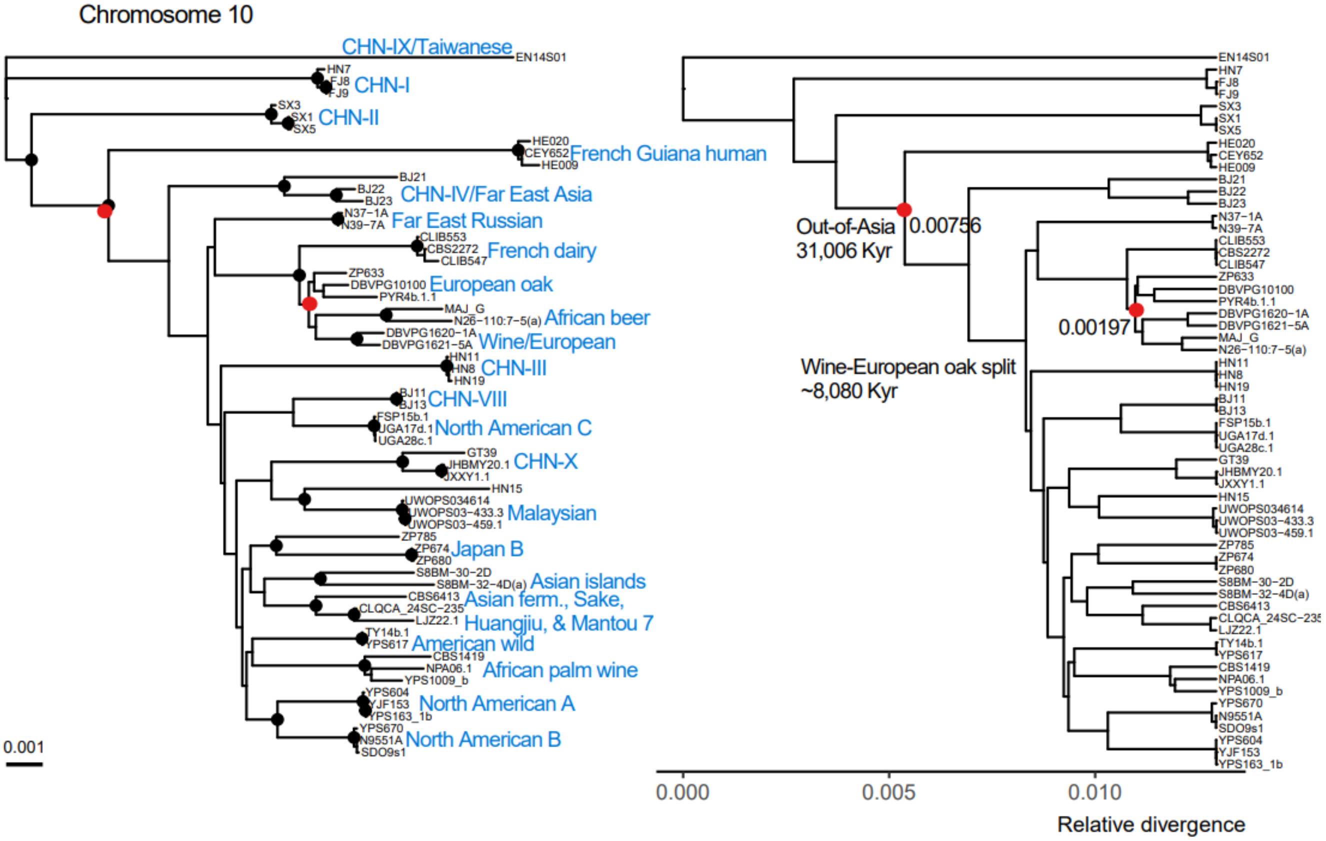
Chromosome 10
CHN-IX/Taiwanese
CHN-I
CHN-II
French Guiana human
CHN-IV/Far East Asia
Far East Russian
French dairy
European oak
African beer
Wine/European
CHN-III
CHN-VIII
North American C
CHN-X
Malaysian
Japan B
Asian islands
Asian ferm., Sake,
Huangjiu, & Mantou 7
American wild
African palm wine
North American A
North American B
0.001
Out-of-Asia
31,006 Kyr
0.00756
0.00197
Wine-European oak split
~8,080 Kyr
0.000
0.005
0.010
Relative divergence

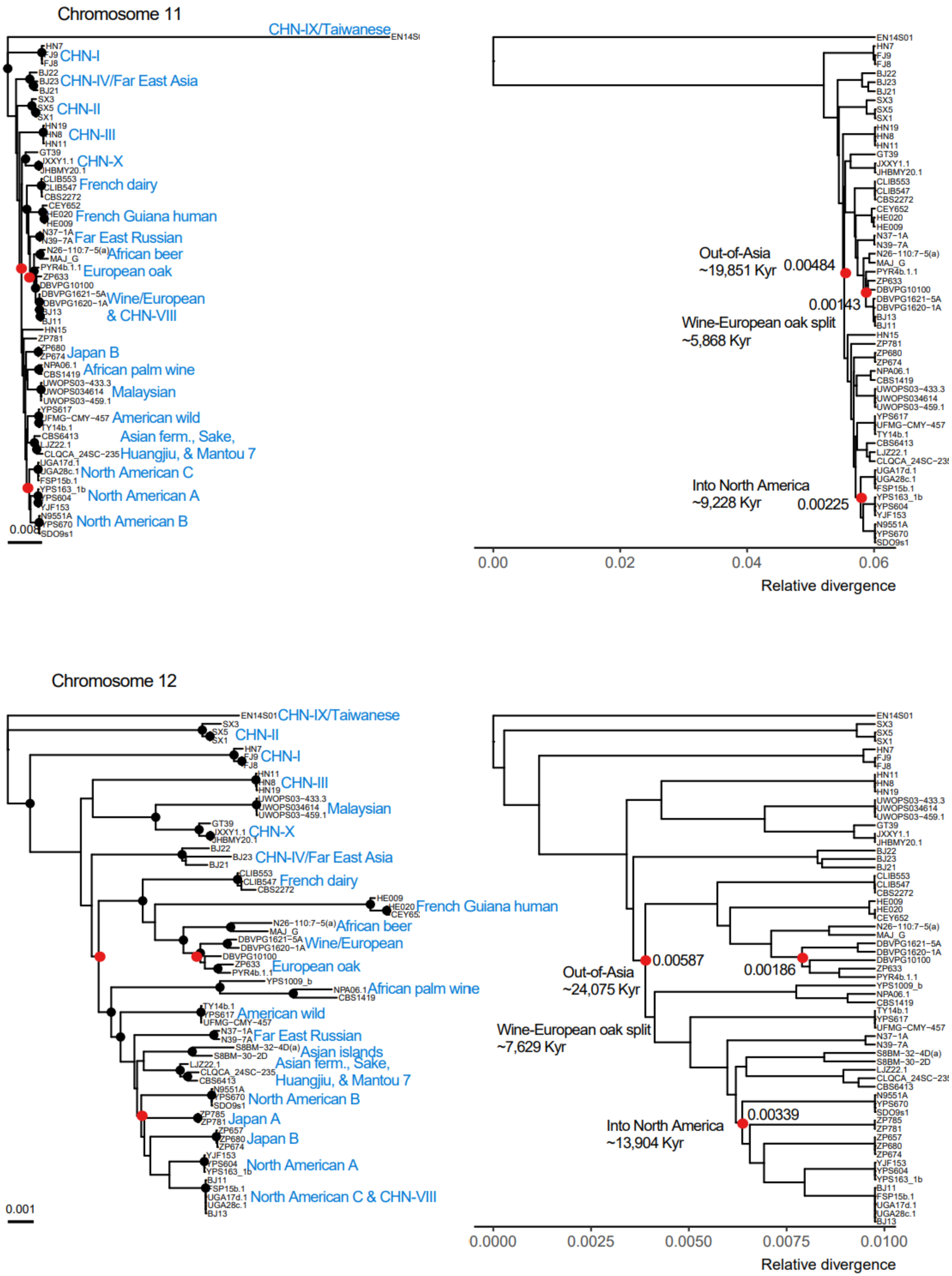
Chromosome 11
CHN-IX/Taiwanese
CHN-I
CHN-IV/Far East Asia
CHN-II
CHN-III
CHN-X
French dairy
French Guiana human
Far East Russian
African beer
European oak
Wine/European & CHN-VIII
Japan B
African palm wine
Malaysian
American wild
Asian ferm., Sake, Huangjiu, & Mantou 7
North American C
North American A
North American B
0.008
Out-of-Asia ~19,851 Kyr
0.00484
0.00143
Wine-European oak split ~5,868 Kyr
Into North America ~9,228 Kyr
0.00225
0.00
0.02
0.04
0.06
Relative divergence
Chromosome 12
CHN-IX/Taiwanese
CHN-II
CHN-I
CHN-III
Malaysian
CHN-X
CHN-IV/Far East Asia
French dairy
French Guiana human
African beer
Wine/European
European oak
African palm wine
American wild
Far East Russian
Asian islands
Asian ferm., Sake, Huangjiu, & Mantou 7
North American B
Japan A
Japan B
North American A
North American C & CHN-VIII
0.001
0.00587
Out-of-Asia ~24,075 Kyr
0.00186
Wine-European oak split ~7,629 Kyr
0.00339
Into North America ~13,904 Kyr
0.0000
0.0025
0.0050
0.0075
0.0100
Relative divergence

Chromosome 13

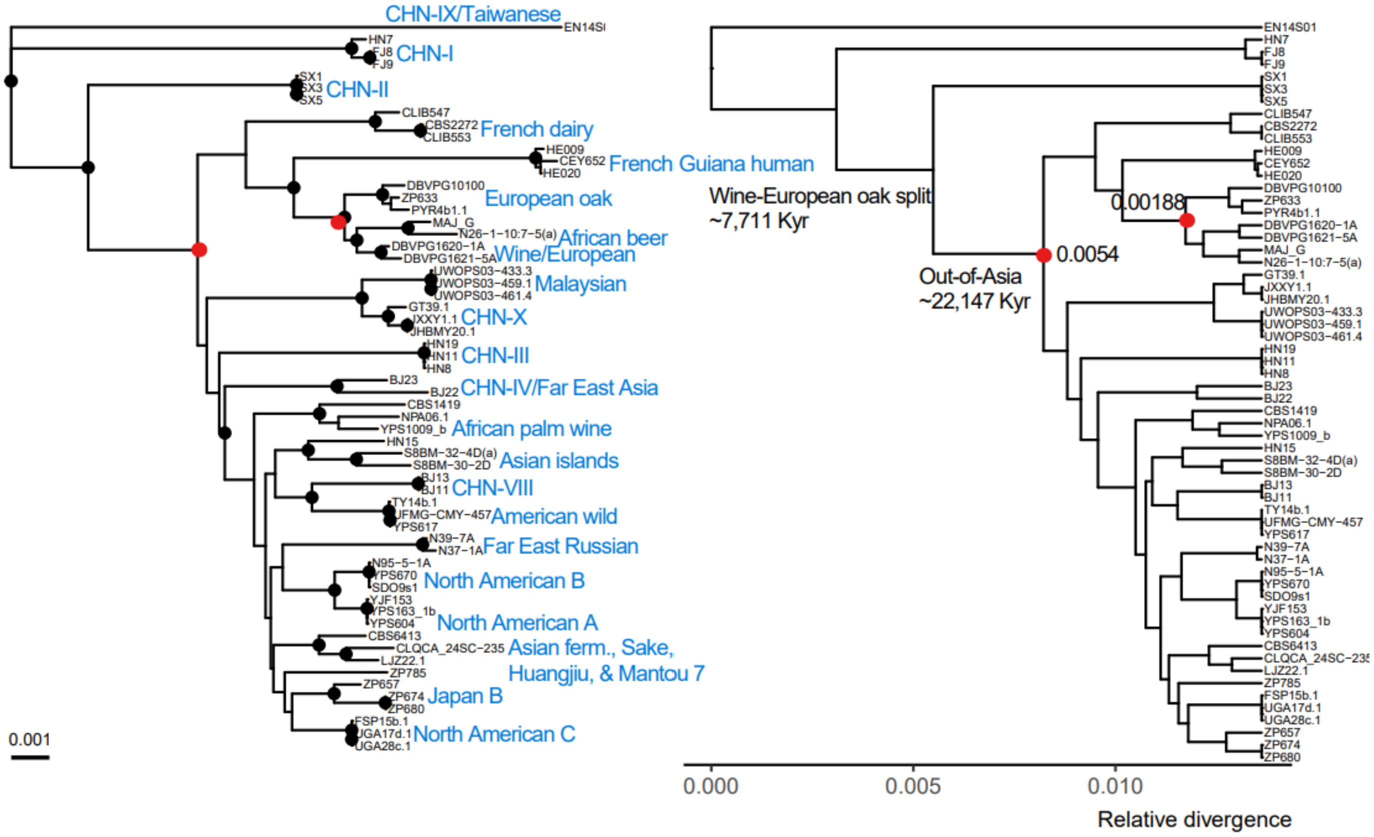

Chromosme 14

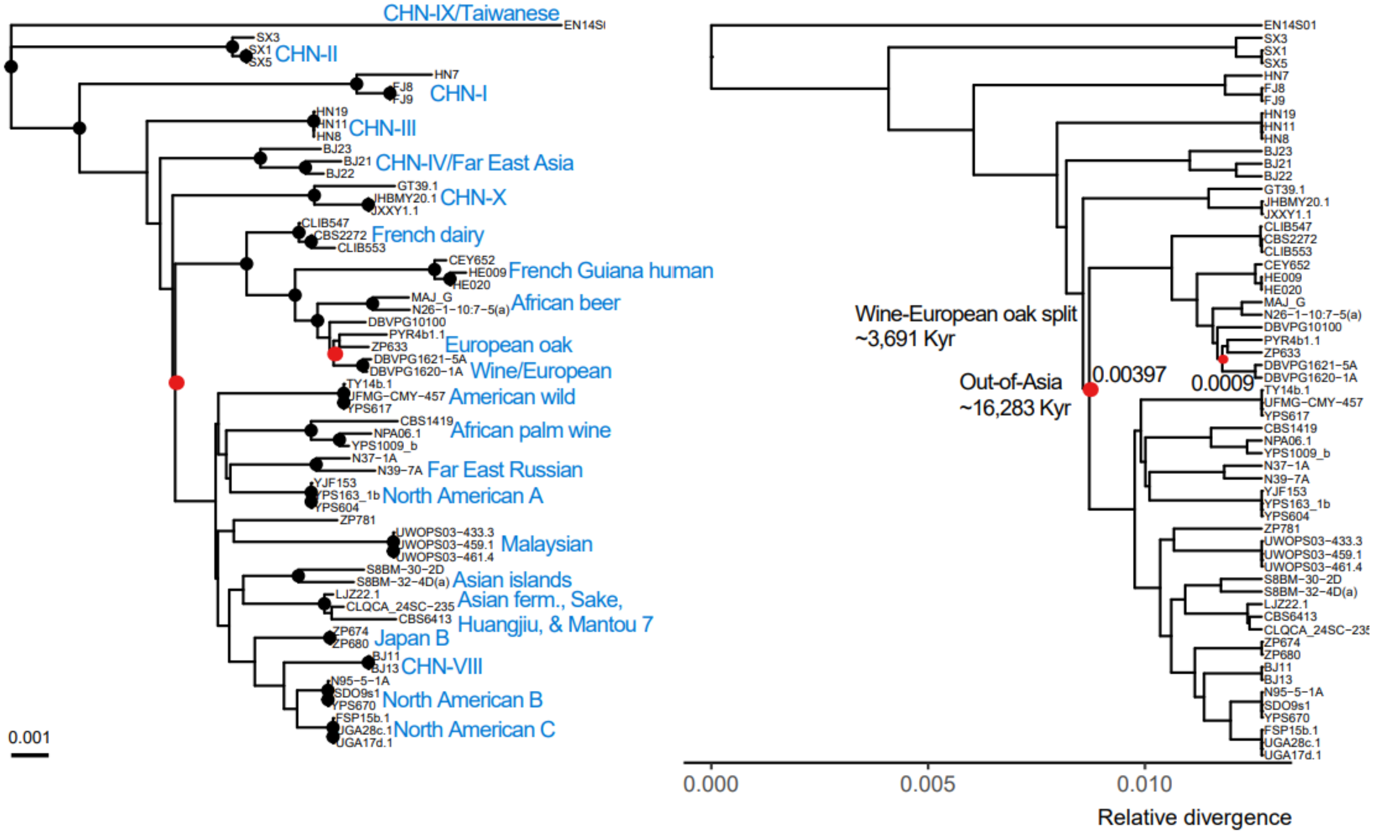

Chromosome 15

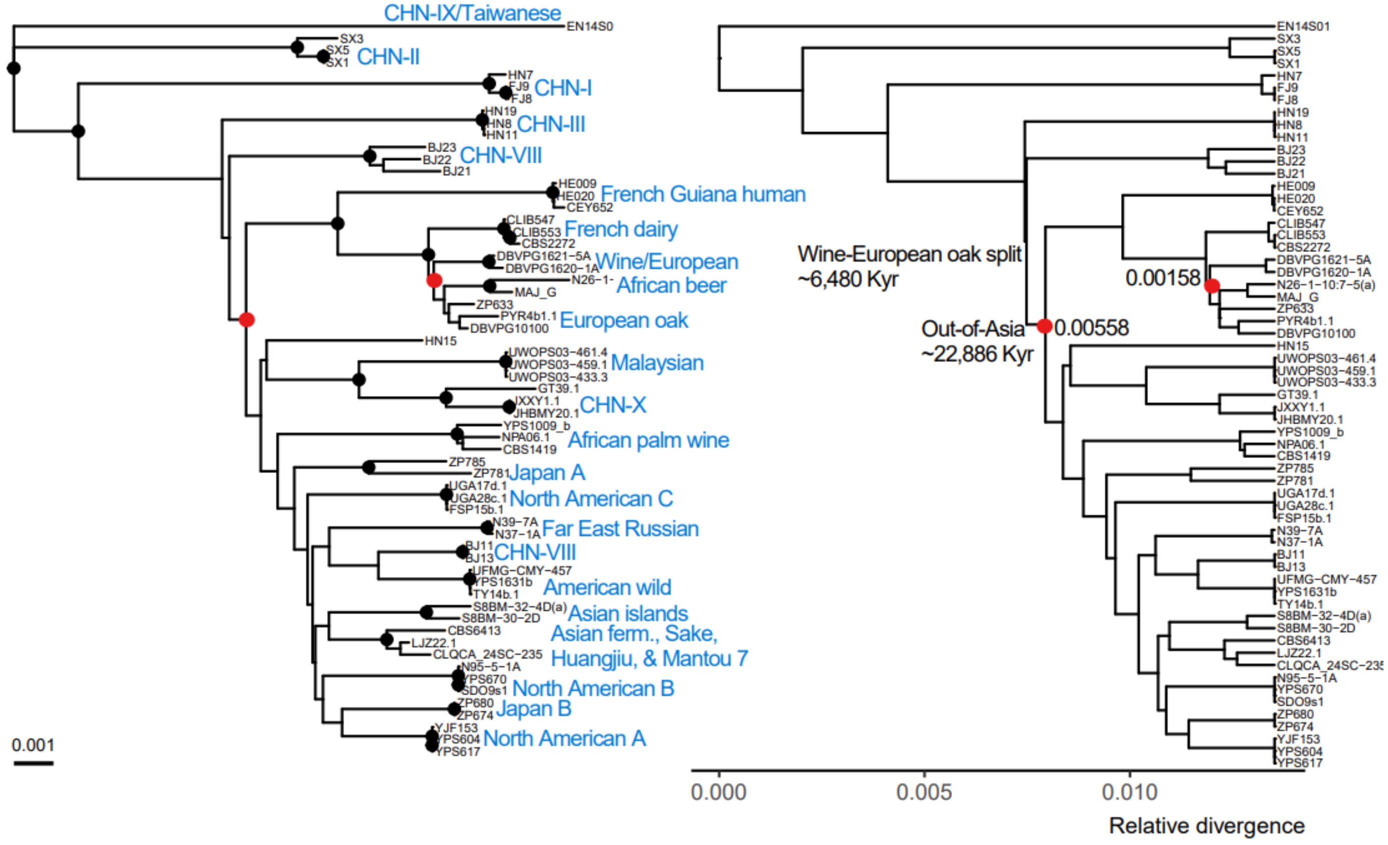

Chromosme 16

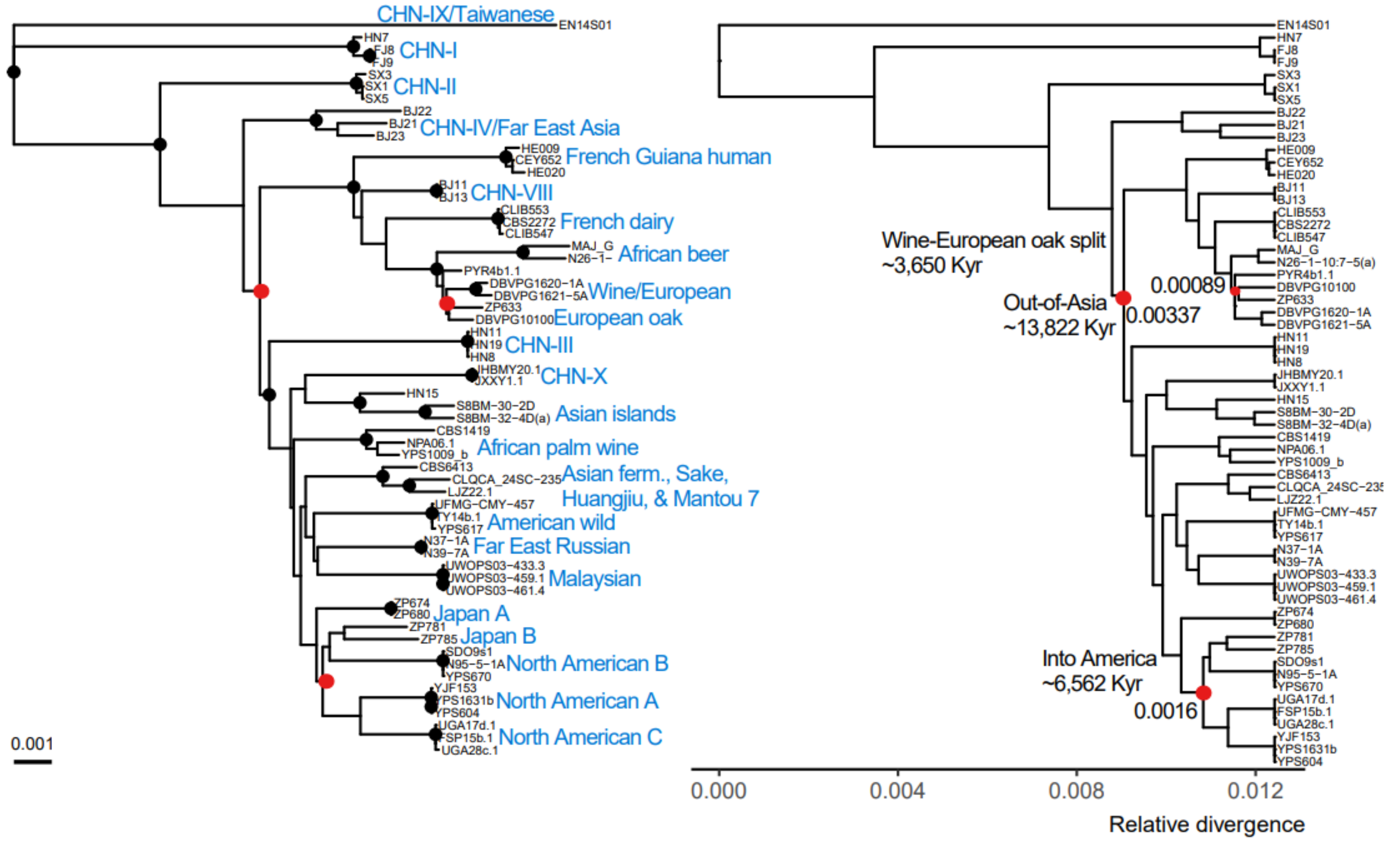

Supplemental Figure 12 Time divergence results of selected 60-90 kb genomic regions of backbone phylogeny strains for each chromosome. Phylogenetic trees on the left are neighborjoining trees of the selected genomic region. The tree was inferred using a Tamura-Nei substitution model (Tamura & Nei, 1993) with a gamma distribution using 100 bootstrap replicates. Black circles at nodes indicate bootstrap support >95%. Clades are labelled in blue text. Time calibrated trees are on the left and were estimated using the RelTime-ML option with default settings (Tamura et al., 2012). We used a CHN-IX/Taiwanese strain (EN14S01) as an outgroup to root the time tree. We calculated the time (T) since the most recent common ancestor (MRCA) in generations per year, TMRCA = k / μ / generations per year, where k is the genetic distance to the MRCA of strains in the clade for that node and μ is the point mutation rate per bp. We used the mutation rate 1.84 x 10-10 from (Fay & Benavides, 2005) where they accounted for 82% of mutations being single base substitutions (Kang et al., 1992) for a mutation rate of 2.25 x 10-10 (Drake, 1991). Time divergence events are indicated with a red circle at a node for the following events: Out-of-Asia, Wine-European oak split, and North America/Japan split. We calculated time estimates for each chromosome from TMRCA estimates (Table S7). Time estimates were not calculated for events when clades did not form monophyletic groups.
